# Design of protein-binding proteins from the target structure alone

**DOI:** 10.1038/s41586-022-04654-9

**Published:** 2022-03-24

**Authors:** Longxing Cao, Brian Coventry, Inna Goreshnik, Buwei Huang, William Sheffler, Joon Sung Park, Kevin M. Jude, Iva Marković, Rameshwar U. Kadam, Koen H. G. Verschueren, Kenneth Verstraete, Scott Thomas Russell Walsh, Nathaniel Bennett, Ashish Phal, Aerin Yang, Lisa Kozodoy, Michelle DeWitt, Lora Picton, Lauren Miller, Eva-Maria Strauch, Nicholas D. DeBouver, Allison Pires, Asim K. Bera, Samer Halabiya, Bradley Hammerson, Wei Yang, Steffen Bernard, Lance Stewart, Ian A. Wilson, Hannele Ruohola-Baker, Joseph Schlessinger, Sangwon Lee, Savvas N. Savvides, K. Christopher Garcia, David Baker

**Affiliations:** 1 Department of Biochemistry, University of Washington, Seattle, WA USA; 2 Institute for Protein Design, University of Washington, Seattle, WA USA; 3 Molecular Engineering Graduate Program, University of Washington, Seattle, WA USA; 4 Department of Bioengineering, University of Washington, Seattle, WA USA; 5 Department of Pharmacology, Yale University School of Medicine, New Haven, CT USA; 6 Howard Hughes Medical Institute, Stanford University School of Medicine, Stanford, CA USA; 7 Department of Structural Biology, Stanford University School of Medicine, Stanford, CA USA; 8 Department of Molecular and Cellular Physiology, Stanford University School of Medicine, Stanford, CA USA; 9 VIB-UGent Center for Inflammation Research, Ghent, Belgium; 10 Unit for Structural Biology, Department of Biochemistry and Microbiology, Ghent University, Ghent, Belgium; 11 Department of Integrative Structural and Computational Biology, The Scripps Research Institute, La Jolla, CA USA; 12 Chemical Biology Laboratory, National Cancer Institute, National Institutes of Health, Frederick, MD USA; 13 J.A.M.E.S. Farm, Clarksville, MD USA; 14 Institute for Stem Cell and Regenerative Medicine, University of Washington, Seattle, WA USA; 15 Deptartment of Pharmaceutical and Biomedical Sciences, University of Georgia, Athens, GA USA; 16 UCB Pharma, Bainbridge Island, WA USA; 17 Seattle Structural Genomics Center for Infectious Disease (SSGCID), Seattle, WA USA; 18 Seattle Children’s Center for Global Infectious Disease Research, Seattle, WA USA; 19 Department of Electrical and Computer Engineering, University of Washington, Seattle, WA USA; 20 The Skaggs Institute for Chemical Biology, The Scripps Research Institute, La Jolla, CA USA; 21 Howard Hughes Medical Institute, University of Washington, Seattle, WA USA

**Keywords:** Protein design, High-throughput screening

## Abstract

The design of proteins that bind to a specific site on the surface of a target protein using no information other than the three-dimensional structure of the target remains a challenge^1–5^. Here we describe a general solution to this problem that starts with a broad exploration of the vast space of possible binding modes to a selected region of a protein surface, and then intensifies the search in the vicinity of the most promising binding modes. We demonstrate the broad applicability of this approach through the de novo design of binding proteins to 12 diverse protein targets with different shapes and surface properties. Biophysical characterization shows that the binders, which are all smaller than 65 amino acids, are hyperstable and, following experimental optimization, bind their targets with nanomolar to picomolar affinities. We succeeded in solving crystal structures of five of the binder–target complexes, and all five closely match the corresponding computational design models. Experimental data on nearly half a million computational designs and hundreds of thousands of point mutants provide detailed feedback on the strengths and limitations of the method and of our current understanding of protein–protein interactions, and should guide improvements of both. Our approach enables the targeted design of binders to sites of interest on a wide variety of proteins for therapeutic and diagnostic applications.

## Abstract (web summary)

A design pipeline is presented whereby binding proteins can be designed de novo without the need for prior information on binding hotspots or fragments from structures of complexes with binding partners.

## Main

Protein interactions have crucial roles in biology, and general approaches to design proteins that disrupt or modulate these interactions would have great utility. Empirical selection approaches that start from large antibody, designed ankyrin repeat protein or other protein scaffold libraries can generate binders to protein targets. However, it is difficult at the outset to target a specific region on a target protein surface and to sample the entire space of possible binding modes. Computational methods can target specific target surface locations and provide a more principled and a potentially faster approach to generate binders than random library selection methods, as well as insight into the fundamental properties of protein interfaces (which must be understood for design to be successful). Most current computational methods used to design proteins that bind to a target surface utilize information derived from structures of the native complex on specific side-chain interactions or protein backbone placements optimal for binding^1–3^. Computational docking of antibody scaffolds with varied loop geometries has yielded binders, but the designed binding modes have rarely been validated with high-resolution structures^4^. Binders have been generated starting from several computationally identified hotspot residues, which were then used to guide the positioning of naturally occurring protein scaffolds^5^. However, for many target proteins, there are no obvious pockets or clefts on the protein surface into which a small number of privileged side chains can be placed, and guidance by a small number of hotspot residues limits the approach to a small fraction of possible interaction modes.

## Design method

**Fig. 1:**
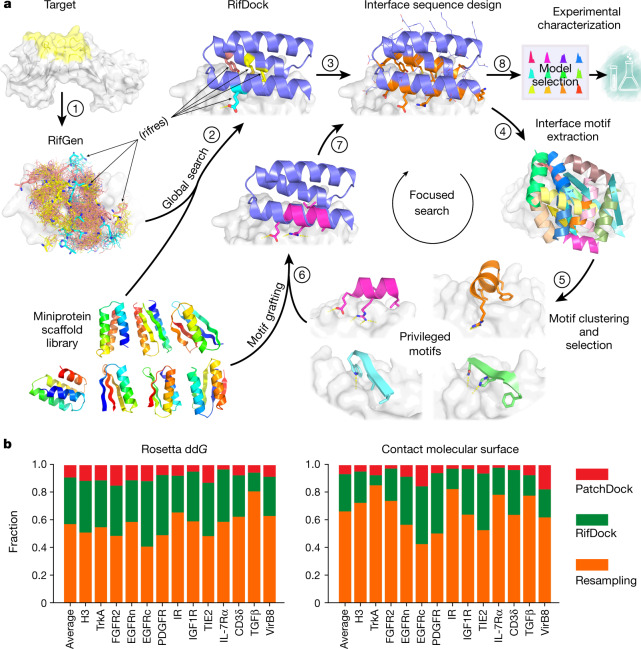
Overview of the de novo protein binder design pipeline. **a**, Schematic of our two-stage binder design approach. In the global search stage, billions of disembodied amino acids are docked onto the selected region of the target protein surface using RifGen, the favourable interacting amino acids are stored as rifres (step 1), and miniprotein scaffolds are then docked on the target guided by these favourable side-chain interactions (step 2). The interface sequences are then designed to maximize interactions with the target (step 3). In the focused search stage, interface structural motifs are extracted and clustered (steps 4 and 5). These privileged motifs are then used to guide another round of docking and design (steps 6 and 7). Designs are then selected for experimental characterization based on computational metrics (step 8). See Extended Data Fig. 1 for a more detailed flow chart of the de novo binder design pipeline. **b**, Comparison of the sampling efficiency of PatchDock, RifDock and resampling protocols. Bar graph shows the distribution over the three approaches of the top 1% of binders based on Rosetta dd*G* and contact molecular surface values after pooling equal-CPU-time dock-and-design trajectories for each of the 13 target sites and averaging per-target distributions (Methods).

**Extended Data Fig. 1:**
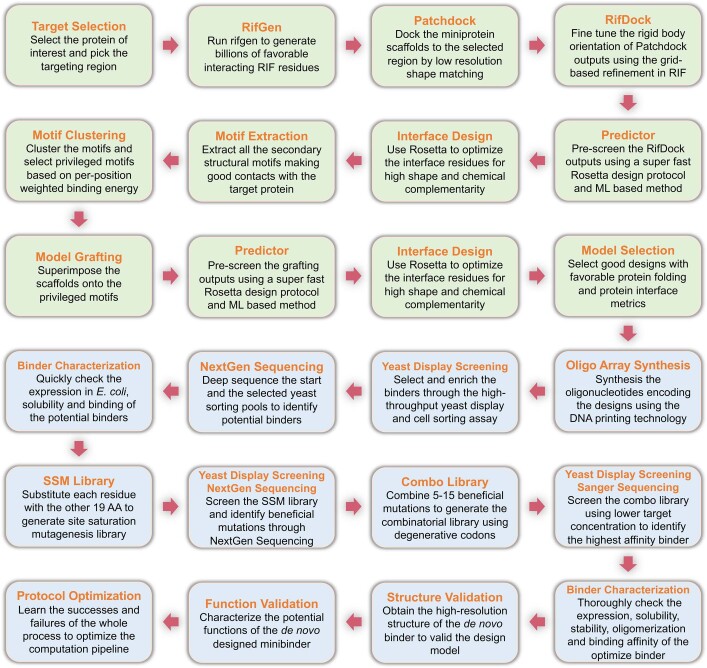
Detailed flow chart of the de novo miniprotein binder design pipeline. The computational design steps are colored as light green and experimental characterization and optimization steps are colored as light blue.

We sought to develop a general approach to the design of high-affinity binders to arbitrary protein targets that addresses two major challenges. First, there are generally no clear side-chain interactions or secondary structure packing arrangements that can mediate strong interactions with the target; instead there are vast numbers of individually very weak possible interactions. Second, the number of ways of choosing which of these numerous weak interactions to incorporate into a single binding protein is combinatorially large, and any given protein backbone is unlikely to be able to simultaneously present side chains that can encompass any preselected subset of these interactions. To illustrate our approach, consider the simple analogy of a difficult climbing wall with only a few suitable footholds or handholds distant from each other. Previous hotspot-based approaches correspond to focusing on routes that involve these footholds and handholds, but this greatly limits possibilities and there may be no way to connect them into a successful route. An alternative is to first identify all the possible handholds and footholds, no matter how poor; second, have thousands of climbers select subsets of these and try to climb the wall; third, identify those routes that showed the most promise, and fourth, have a second group of climbers explore them in detail. Following this analogy, we devised the following multistep approach to overcome the above two challenges: step (1), enumerate a large and comprehensive set of disembodied side-chain interactions with the target surface; step (2), identify from large in silico libraries of protein backbones those that can host many of these side chains without clashing with the target; step (3), identify recurrent backbone motifs in these structures; and step (4), generate and place against the target a second round of scaffolds that contain these interacting motifs (Fig. 1a and Extended Data Fig. 1). Steps (1) and (2) widely search the space, whereas steps (3) and (4) intensify the search in the regions that show the most promise. We describe each step in more detail below.

We began by docking disembodied amino acids against the target protein and storing the backbone coordinates and target binding energies of the typically billions of amino acids that make favourable hydrogen bonding or nonpolar interactions in a six-dimensional spatial hash table for rapid look-up (Fig. 1a and Methods). This rotamer interaction field (RIF) enables rapid approximation of the target interaction energy achievable by a protein scaffold docked against a target based on its backbone coordinates alone (with no need for time-consuming side-chain sampling). For each dock, the target interaction energies of each of the matching amino acids in the hash table are summed. A related approach was used for the design of small-molecule binders^6^; as protein targets are so much bigger and because nonpolar interactions are the primary driving force for protein–protein interactions, we focused the RIF generation process on nonpolar sites in specific surface regions of interest. For example, for the design of inhibitors, we focused on interaction sites with biological partners. The RIF approach improves on previous discrete interaction-sampling approaches^5^ by reducing the algorithmic complexity from O(*N*) or O(*N*^2^) to O(1) with respect to the number of side-chain–target interactions considered, thereby allowing for billions, rather than thousands, of potential interfaces to be considered.

For docking against the RIF, it is desirable to have a large set of protein scaffold options, as the chance that any one scaffold can house many interactions is small. The structure models of these scaffolds must be quite accurate so that the positioning is correct. Using fragment assembly^7^, piecewise fragment assembly^8^ and helical extension^9^, we designed a large set of miniproteins that ranged in length from 50 to 65 amino acids and contained larger hydrophobic cores than previous miniprotein scaffold libraries^1^. These properties make the protein more stable and more tolerant to the introduction of the designed binding surfaces. A total of 84,690 scaffolds spanning 5 different topologies with structural metrics predictive of folding were encoded in large oligonucleotide arrays, and 34,507 of these were found to be stable using a high-throughput proteolysis-based protein stability assay^10^.

**Extended Data Fig. 2:**
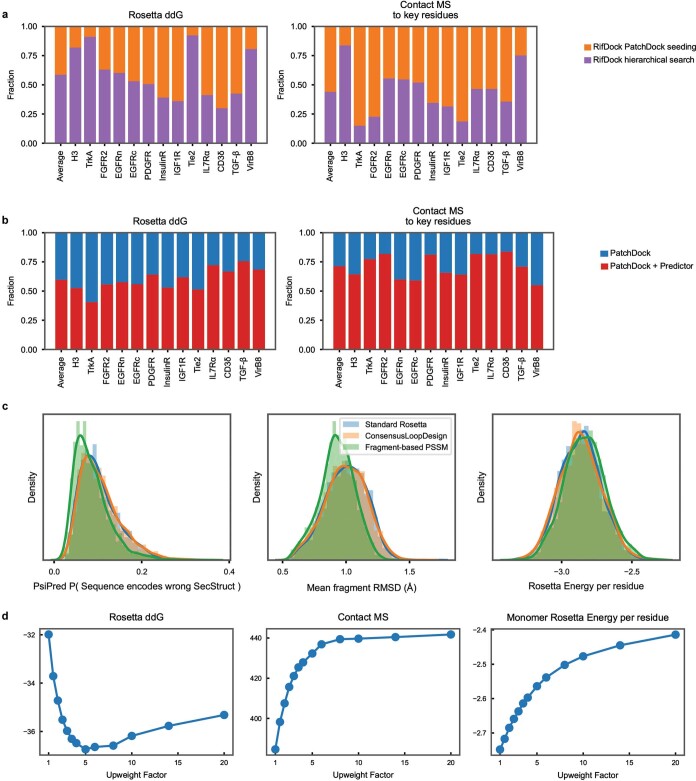
Analysis of the critical steps of the de novo binder design pipeline. **a**, Comparison of the two docking approaches based on Rosetta ddG and contact molecular surface. Average and per-target distribution of the top 1% of binders in two key metrics after pooling equal-CPU-time dock-and-design trajectories. RifDock seeded with PatchDock outputs generated 300 outputs per scaffold that were trimmed to a total of 19,500 docks with “The Predictor” and designed using combinatorial side-chain optimization (orange). RifDock using the Hierarchical docking search generated 300 outputs per scaffold that were trimmed to a total of 19,500 docks with “The Predictor” and subsequently designed (purple). Rosetta ddG refers to the predicted binding energy as calculated by Rosetta and Contact MS to key residues refers to the Contact Molecular Surface value (a distance weighted interfacial area calculation) to the key hydrophobic residues on the target that define this binding site. **b**, The rapid pre-screening method enriches docks with better Rosetta ddG and contact molecular surface. Average and per-target distribution of the top 1% of binders in two key metrics after pooling equal-CPU-time dock-and-design trajectories. The top 30 PatchDock outputs for the 1,000 helical scaffolds tested were designed using the RosettaScripts protocol (blue). The top 300 PatchDock outputs for the 1,000 helical scaffolds tested were trimmed to 21,000 with “The Predictor” and subsequently designed (red). **c**, The improved sequence design protocol yielded amino acid sequences more strongly predicted to fold to the monomer structure. The effect on fragment quality and Rosetta Score with different fragment-quality-guidance approaches. Rosetta using FastDesign with the standard LayerDesign settings was used to design 1,000 3-helical and 1,000 4-helical mini-protein scaffolds (blue). The same protocol was supplanted with the ConsensusLoopDesign TaskOperation (orange). The structure-based PSSM was used as an energy term in addition to the Standard Rosetta protocol (green). Two predictors of sequence-structure correspondence were found to improve without negatively affecting the computed Rosetta score of the binders. The probability that the designed sequence encoded for the wrong secondary structure was computed using PsiPred4^88^ (left), and for each 9aa fragment of the designed scaffold, the closest match to a fragment in the Protein Data Bank with the same sequence was computed and averaged over the entire structure^10^ (center). Details can be found in the Supplemental Information. **d**, The improved sequence design protocol yielded amino acid sequences more strongly bound to the target. 10,000 scaffolds docked against the N-terminal domain of EGFR were designed with the RosettaScripts protocol while varying only the weight of the ProteinProteinInterfaceUpweighter. This TaskOperation multiplies all energies across the interface by the listed value during packing-design calculations.

We experimented with several approaches for docking these stable scaffolds against the target structure RIF, balancing overall shape complementarity with maximizing specific rotamer interactions. The most robust results were obtained using direct low-resolution shape matching^11^ followed by grid-based refinement of the rigid body orientation in the RIF (RIFDock). This approach resulted in better Rosetta binding energy (dd*G*) values and packing (contact molecular surface, see below) after sequence design than shape matching alone with PatchDock (Fig. 1b, red and green), and more extensive nonpolar interactions with the target than hierarchical search without PatchDock shape matching (Extended Data Fig. 2a) ^6^.

Because of the loss in resolution in the hashing used to build the RIF, and the necessarily approximate accounting for interactions between side chains (Methods), we found that evaluation of the RIF solutions was considerably enhanced by full combinatorial optimization using the Rosetta forcefield, which allow the target side chains to repack and the scaffold backbone to relax. However, full combinatorial sequence optimization is CPU intensive. To enable efficient screening of millions of alternative backbone placements, we developed a rapid interface pre-screening method using Rosetta to identify promising RIF docks. Restricting to hydrophobic amino acids and considering a smaller number of side-chain rotamers than in standard Rosetta design calculations, together with a more rapidly computable energy function sped up the design time by more than tenfold while retaining a strong correlation with results after full sequence design (next paragraph). This pre-screen (referred to as the ‘Predictor’ below) substantially improved the binding energies and shape complementarity of the final designs, as far more RIF solutions could be processed (Extended Data Fig. 2b).

We observed that application of the standard Rosetta design to the set of filtered docks in some cases resulted in models with buried unsatisfied polar groups and other suboptimal properties. To overcome these limitations, we developed a combinatorial sequence design protocol that maximizes shape and chemical complementarity with the target while avoiding buried polar atoms. Sequence compatibility with the scaffold monomer structure was increased using a structure-based sequence profile^12^, cross-interface interactions were upweighted during the Monte-Carlo-based sequence design stage to maximize the contacts between the binder and the target (ProteinProteinInterfaceUpweighter; Methods) and rotamers that contained buried unsatisfiable polar atoms were eliminated before packing and buried unsatisfied polar atoms penalized by a pair-wise decomposable pseudo-energy term^13^. This protocol yielded amino acid sequences that were more strongly predicted to fold to the designed structure (Extended Data Fig. 2c) and to bind the target (Extended Data Fig. 2d) than standard Rosetta interface design.

In the course of developing the overall binder design pipeline, we noted after inspection that even designs with favourable Rosetta binding free energies, large changes in the solvent-accessible surface area (SASA) after binding and high shape complementarity (SC) often lacked dense packing and interactions that involve several secondary structural elements. We developed a quantitative measure of packing quality in closer accord with visual assessment—the contact molecular surface (Methods)—which balances interface complementarity and size in a manner that explicitly penalizes poor packing. We used this metric to help to select suitable designs at both the rapid Predictor stage and after full sequence optimization (Methods).

The space sampled by the search across the structure and sequence space is enormous: tens of thousands of possible protein backbones × nearly 1 billion possible disembodied side-chain interactions per target × 10^16^ interface sequences per scaffold placement. Sampling of spaces of this size is necessarily incomplete, and many of the designs at this stage contained buried unsatisfied polar atoms (only rotamers that cannot make hydrogen bonds in any context are excluded at the packing stage) and cavities. To generate improved designs, we intensified the search around the best of the designed interfaces. We developed a resampling protocol that first extracts all the secondary structural motifs that make good contacts with the target protein from the first ‘broad search’ designs. Next, it clusters these motifs on the basis of their backbone coordinates and rigid body placements, and then selects the binding motif in each cluster with the best per-position weighted Rosetta binding energy. Using this approach, around 2,000 motifs were selected for each target. These motifs, which in many cases resemble canonical secondary structure packing patterns^14^, are privileged because they contain a much greater density of favourable side-chain interactions with the target than the rest of the designs. The motifs were used to guide another round of docking and design. First, scaffolds from the library were superimposed on the motifs and the favourable-interacting motif residues transferred to the scaffold. The remainder of the scaffold sequence was optimized to make further interactions with the target, allowing backbone flexibility through backbone torsion-angle minimization to increase shape complementarity with the target (Fig. 1a). Design Interface metrics following resampling were considerably improved over those from the broad searching stage (Fig. 1b). The designs with the most favourable protein folding and protein interface metrics from both the broad searching and resampling stages were selected for experimental validation.

## Experimental testing

**Fig. 2:**
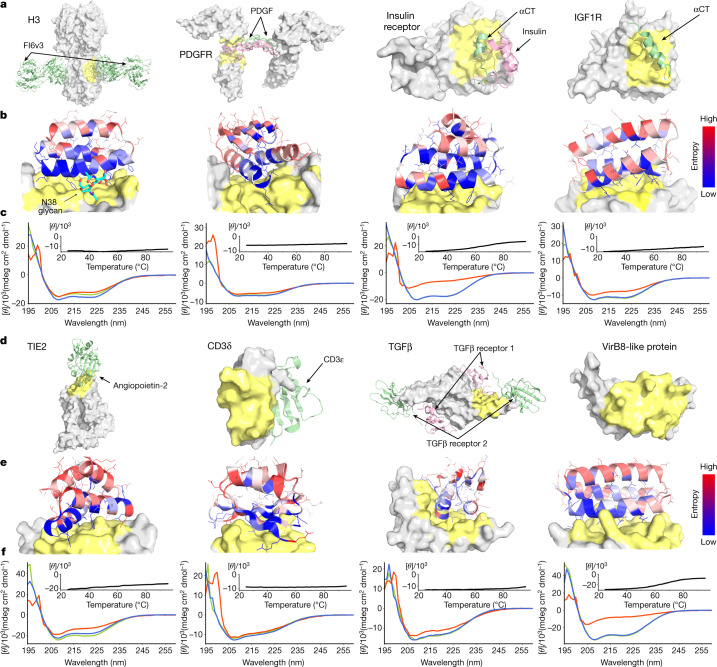
De novo design and characterization of miniprotein binders. **a**, **d**, Naturally occurring target protein structures shown in surface representation, with known interacting partners in cartoons where available. Regions targeted for binder design are coloured in pale yellow or green; the remainder of the target surface is in grey. See Extended Data Fig. 3 for side-by-side comparisons of the native binding partners and the computational design models. The PDB identifiers are 3ZTJ (H3), 3MJG (PDGFR), 4OGA (IR), 5U8R (IGF1R), 2GY7 (TIE2), 1XIW (CD3δ), 3KFD (TGFβ) and 4O3V (VirB8). αCT, α-chain C-terminal helix. **b**, **e**, Computational models of designed complexes coloured by site saturation mutagenesis results. Designed binding proteins (cartoons) are coloured by positional Shannon entropy, with blue indicating positions of low entropy (conserved) and red those of high entropy (not conserved); the target surface is in grey and yellow. The core residues and binding interface residues are more conserved than the non-interface surface positions, consistent with the computational models. Full SSM maps over all positions of all the de novo designs are provided in the Supplementary Information. **c**, **f**, Circular dichroism spectra at different temperatures (green, 25 °C; red, 95 °C; blue, 95 °C followed by 25 °C), and circular dichroism signals at 222-nm wavelength as a function of temperature for the optimized designs (insets). See Extended Data Fig. 4 for the biolayer interferometry characterization results of the optimized designs.

**Fig. 3:**
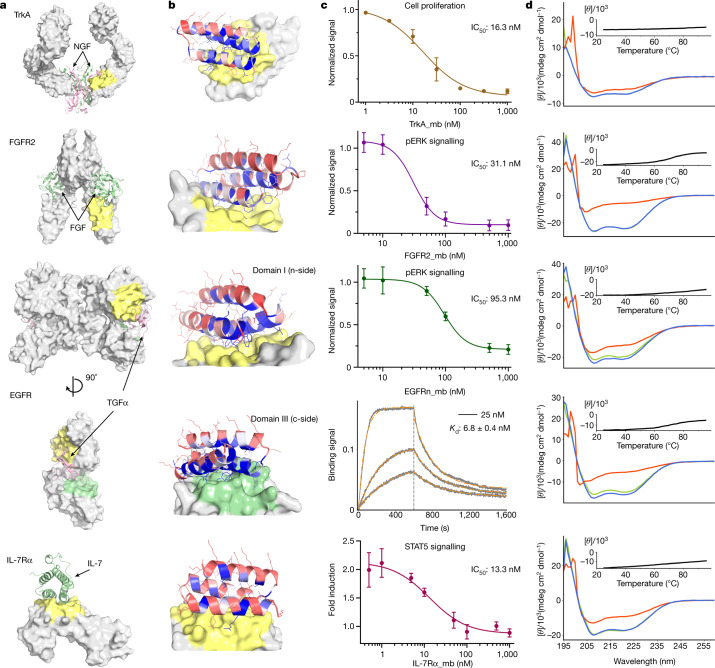
De novo design and inhibition of native signalling pathways by designed miniproteins. See the panel descriptions in Fig. 2 legend for **a**, **b**, **d**. The PDB identifiers are 2IFG (TrkA), 1DJS (FGFR2), 1MOX (EGFR) and 3DI3 (IL-7Rα) for **a**. **c**, For TrkA, the dose-dependent reduction in cell proliferation after 48 h of TF-1 cells with increasing TrkA minibinder (TrkA_mb) concentration is shown. (8.0 ng ml^–1^ human β-NGF was used for competition). Titration curves at different concentrations of NGF and the effects of the miniprotein binders on cell viability are presented in Extended Data Fig. 8. For FGFR2, the dose-dependent reduction pERK signalling elicited by 0.75 nM β-FGF in human umbilical vein endothelial cells (HUVECs) with increasing FGFR2 minibinder (FGFR2_mb) concentration is shown. For the EGFRn-side binder, the dose-dependent reduction in pERK signalling elicited by 1 nM EGF in HUVECs with increasing EGFRn-side minibinder (EGFRn_mb) concentration is shown. See Extended Data Fig. 9 and Methods for experimental details. For the EGFRc-side binder, biolayer interferometry results are shown. See Extended Data Fig. 4 for the biolayer interferometry characterization results of the other optimized designs. For IL-7R, the reduction in STAT5 activity induced by 50 pM of IL-7 in HEK293T cells in the presence of increasing IL-7Rα minibinder (IL-7Rα_mb) concentrations is shown. The mean values were calculated from triplicates for the cell signalling inhibition assays measured in parallel, and error bars represent standard deviations. IC_50_ was calculated using a four-parameter-logistic equation in GraphPad Prism 9 software.

**Extended Data Fig. 3:**
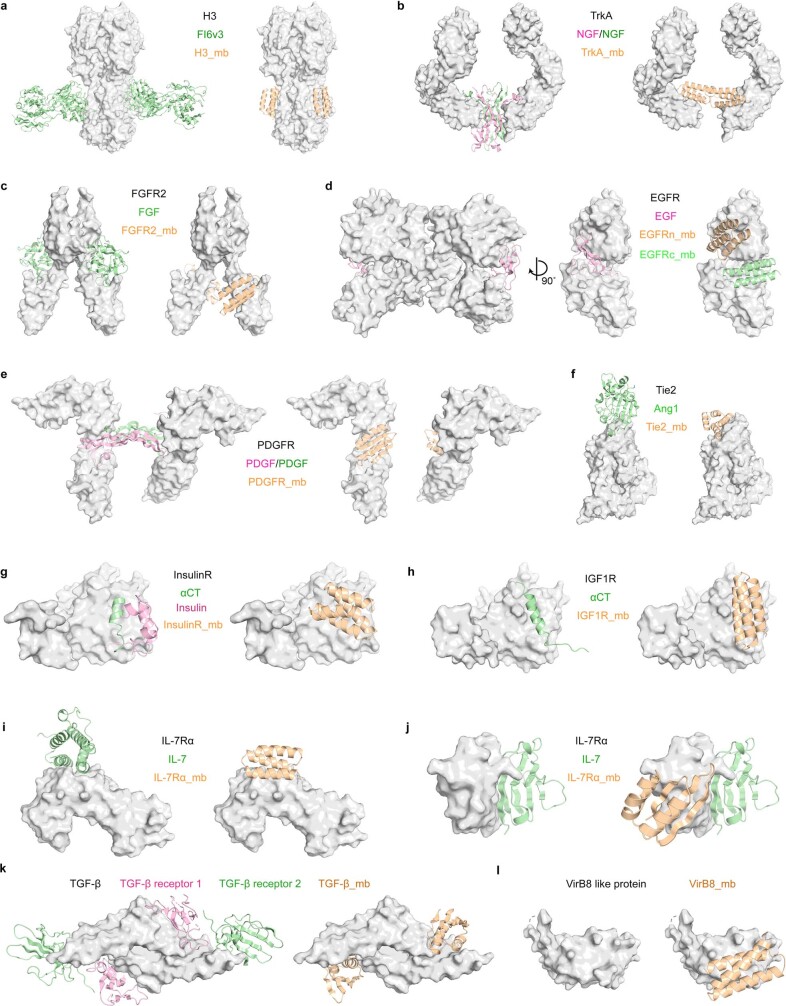
Comparison of the native binding partners and the computational design models. Side-by-side comparison of the native binding partners of the selected targets and the binding configurations of the computational designed models.

Previous approaches used to design protein binders have been tested on only one or two targets, which limits assessment of their generality. To thoroughly test our new binder design pipeline, we selected 13 native proteins of considerable current interest and spanning a wide range of shapes and biological functions. These proteins fall into two classes: (1) human cell surface or extracellular proteins involved in signalling, and (2) pathogen surface proteins. Binders for human cell surface or extracellular proteins could have utility as probes of biological mechanism and potentially as therapeutics, and hence we sought to design binders to tropomyosin receptor kinase A (TrkA; also known as NTRK1)^15^, fibroblast growth factor receptor 2 (FGFR2)^16^, epidermal growth factor receptor (EGFR)^17^, platelet-derived growth factor receptor (PDGFR)^18^, insulin receptor (IR)^19^, insulin-like growth factor 1 receptor (IGF1R)^20^, angiopoietin-1 receptor (TIE2)^21^, interleukin-7 receptor-α (IL-7Rα)^22^, CD3 delta chain (CD3δ)^23^ and transforming growth factor-β (TGFβ)^24^. Binding proteins for pathogen surface proteins could also have therapeutic utility, and so we also designed binders to influenza A H3 haemagglutinin (H3)^25^, VirB8-like protein from *Rickettsia typhi* (VirB8)^26^ and the SARS-CoV-2 coronavirus spike protein (Figs. 2 and 3). For each of these surface proteins, we selected one or two regions for the binders to target to ensure maximal biological utility and for potential downstream therapeutic potential. These regions span a wide range of surface properties, with diverse shape and chemical characteristics (Figs. 2 and 3, and Extended Data Fig. 3). Some of the selected targeting regions overlap with the native interfaces, but no native interface information or native hotspots were used during the binder design process. For some targets (for example, CD3δ and VirB8), no structures of the native complex were available and there were no proteins known to bind at the targeted region.

Using the above protocol, we designed 15,000–100,000 binders for each of the 13 target sites on the 12 native proteins (Methods; we chose two sites for EGFR). Synthetic oligonucleotides (230 base pairs) encoding the 50–65 residue designs were cloned into a yeast surface-expression vector so that the designs were displayed on the surface of yeast. Those that bound their target were enriched by several rounds of fluorescence-activated cell sorting (FACS) using fluorescently labelled target proteins. The starting and enriched populations were deep sequenced, and the frequency of each design in the starting population and after each sort was determined. From multiple sorting rounds at different target protein concentrations, we determined, as a proxy for the binding dissociation constant (*K*_d_) values, the midpoint concentration (SC_50_) in the binding transitions for each design in the library (Extended Data Table 1 and Methods).

To assess whether the top enriched designs for each target fold and bind as in the corresponding computational design models, and to investigate the sequence dependence of folding and binding, we generated high-resolution footprints of the binding surface by sorting site saturation mutagenesis libraries (SSMs) in which every residue was substituted with each of the 20 amino acids one at a time. For the majority of the enriched designs, substitutions at the binding interface and in the protein core were less tolerated than substitutions at non-interface surface positions (Figs. 2 and 3, and Extended Data Fig. 5), and all of the cysteine residues were highly conserved in designs that contained disulfides. The effects of each mutation on both binding energy and monomer stability were estimated using Rosetta design calculations, and a reasonable correlation was found between the predicted and experimentally determined effect of mutations (Extended Data Fig. 6a). In almost all cases, a small number of substitutions increased the apparent binding affinity, and we generated libraries combining 5–15 of these and sorted them for binding under increasingly stringent (lower target concentration) conditions. Many of these affinity-enhancing substitutions were mutations to tyrosine (Extended Data Fig. 6b), which is consistent with the relatively high frequency of tyrosine in natural protein interfaces^27^. The set of affinity-increasing substitutions provide valuable information to improve the binder design approach, as these substitutions ideally would have been identified in the computational sequence design calculations (see ‘Discussion’ for more details).

**Extended Data Fig. 4:**
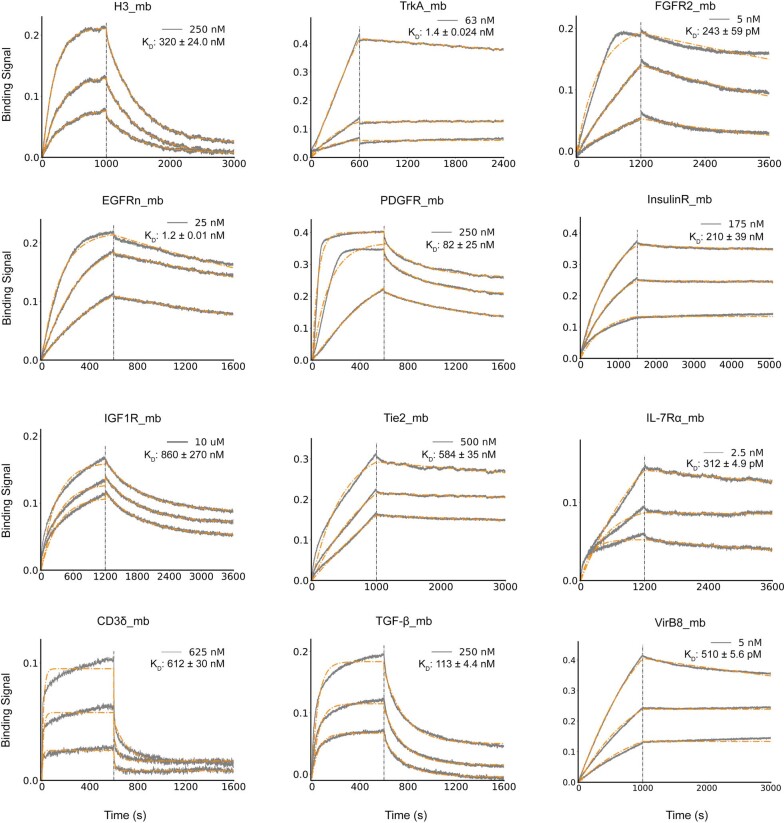
Biolayer interferometry characterization of binding of optimized designs to the corresponding targets. Two-fold serial dilutions were tested for each binder and the highest concentration is labeled. For H3, TrkA, FGFR2, EGFR, PDGFR, IL-7Rα, CD3δ, TGF-β and VirB8, the biotinylated target proteins were loaded onto the Streptavidin (SA) biosensors, and incubated with miniprotein binders in solution to measure association and dissociation. For IGF1R and Tie2, MBP- (maltose binding protein) tagged miniprotein binders were used as the analytes. For InsulinR, the miniprotein binder was immobilized onto the Amine Reactive Second-Generation (AR2G) Biosensors and the insulin receptor was used as the analyte. The gray color represents experimental data and orange color represents fit curves. The fitting curves are poor at high binder concentrations due to the self- association of the binders through the interface hydrophobic residues, so we only kept the traces and fits at low binder concentrations.

**Extended Data Fig. 5:**
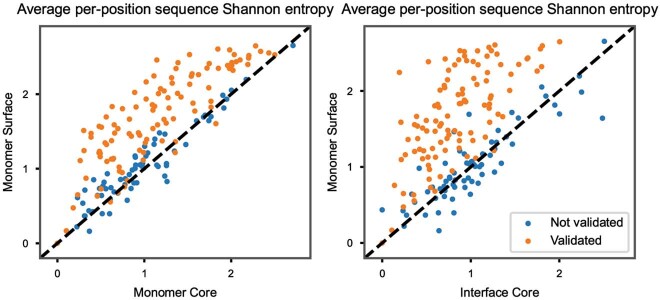
Average SSM sequence entropy for different regions of binders. The sequence entropy of a single position was calculated by looking at the counts from the sort with the concentration closest to 10-fold lower than the estimated parent SC_50_ and performing a simple Shannon entropy calculation on all amino acids observed at that position. Each plotted point is the average entropy of all positions within each of the three zones respectively. Validated vs Not Validated refers to the SSM Validation procedure with a cutoff of 0.005 (see Methods and **Extended Data Figure 15**). Since one would expect the core residues of the monomer and core residues of the interface to be conserved while the surface residues should not matter, the validated binders trend above the line. Points on the line do not show a difference between their surfaces and cores, potentially indicating unfolded or misfolded proteins. Points below the line may be misfolded or binding with alternate residues.

**Extended Data Fig. 6:**
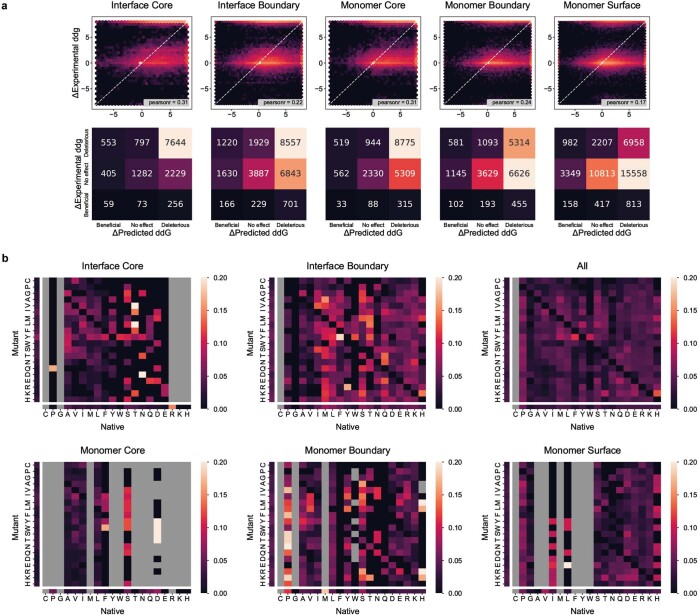
Computational analysis of the experimental SSM results. **a**, Ability of Rosetta to predict mutational effects. This graph shows the observed experimental effect of each mutation versus Rosetta’s expected effect. For each plotted point, the delta refers to the effect versus the parent SSM design; therefore a “Beneficial” mutation is one that would improve affinity relative to the original designed protein the SSM was based on. The ΔExperimental ddg is derived from FACS data using the SC_50_ values (see Methods). Confidence intervals were collapsed to their center point to make this graph and “No effect” refers to mutations with less than a 1 kcal/mol change. Binder region definitions: Interface Core: residue contacts target protein and has no SASA (Solvent Accessible Surface Area) in bound state; Interface Boundary: residue contacts target protein, but does have SASA; Monomer Core: residue has no SASA and does not contact target; Monomer Boundary: residue has intermediate SASA and does not contact target; Monomer Surface: residue has full SASA and does not contact target. see Methods SSM Validation for further explanation. **b**, Mutations observed in SSM experiments that improved affinity bind at least 1kcal/mol graphed by relative frequency. Plotted is the #_times_Native_to_Mutant_improved_affinity / #_times_Native_to_Mutant_tested_in SSMs. A value of 0.10 with x-axis F and y-axis W could therefore represent that for 2 of 20 times W was substituted for Y, the affinity improved. Separated bars on each axis represent pooled data for the entire row/column. Grey boxes indicate mutations that occurred fewer than 5 times. Only SSM designs with a validation score of 0.005 or better were considered. While some cells are clipped, none extended beyond 0.25. Binder region definitions: Interface Core: residue contacts target protein and has no SASA in bound state; Interface Boundary: residue contacts target protein, but does have SASA; Monomer Core: residue has no SASA and does not contact target; Monomer Boundary: residue has intermediate SASA and does not contact target; Monomer Surface: residue has full SASA and does not contact target.

We expressed the highest affinity combinatorially optimized binders for each target in *Escherichia coli* to enable more detailed structural and functional characterization. All of the designs were in the soluble fraction and could be readily purified by Ni^2+^-NTA chromatography. All had circular dichroism spectra consistent with the design model, and most (9 out of 13) were stable at 95 °C (Figs. 2 and 3, and Table 1). The binding affinities for the targets were assessed by biolayer interferometry and values ranged from 300 pM to 900 nM (Fig. 3, Table 1 and Extended Data Fig. 4). The sequence mapping data report on the residues in the design that are crucial for binding, but only weakly on the region of the target bound. We investigated the latter using a combination of binding competition experiments, biological assays and structural characterization of the complexes. For the nine targets for which these were available, this characterization suggested binding modes consistent with the design models, as described in the subsequent paragraphs.

**Table 1:** Physicochemical properties of the optimized de novo miniprotein binders

	H3	TrkA	FGFR2	EGFRn	EGFRc	PDGFR	IR	IGF1R	TIE2	IL-7Rα	CD3δ	TGFβ	VirB8
*K*_d_ (nM)	320 ± 24.0	1.4 ± 0.02	243 ± 59.0	1.2 ± 0.01	6.8 ± 0.3	82 ± 25	210 ± 39	860 ± 270	584 ± 35	0.31 ± 0.004	612 ± 30	113 ± 4.4	0.51 ± 0.005
*T*_M_ (°C)	> 95.0	> 95.0	71.1	> 95.0	71.2	> 95.0	65.0	> 95.0	> 95.0	> 95.0	> 95.0	> 95.0	66.2

The binding affinity and melting temperature (*T*_M_) of the optimized de novo miniprotein binders. See Figs. 2 and 3 for the circular dichroism spectra; the raw biolayer interferometry traces are in Extended Data Fig. 4. Experimental details can be found in the corresponding figure legends and section of the Methods.

## Cell receptors involved in signalling

**Extended Data Fig. 7:**
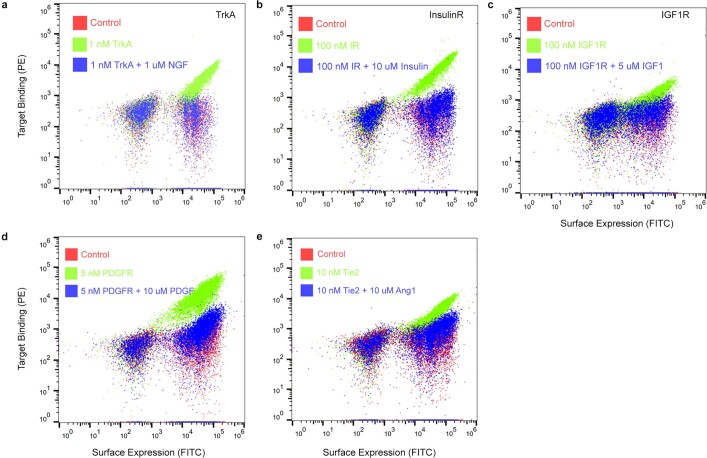
Competition experiments indicated the miniprotein binders bound to the targeted region. Yeast cells displaying the TrkA binder (**a**), InsulinR binder (**b**), IGF1R binder (**c**), PDGFR binder (**d**) and Tie2 binder (**e**) were incubated with the target protein in the presence or absence of the native ligand as the competitor, and target protein binding to cells (y axis) was monitored with flow cytometry.

**Extended Data Fig. 8:**
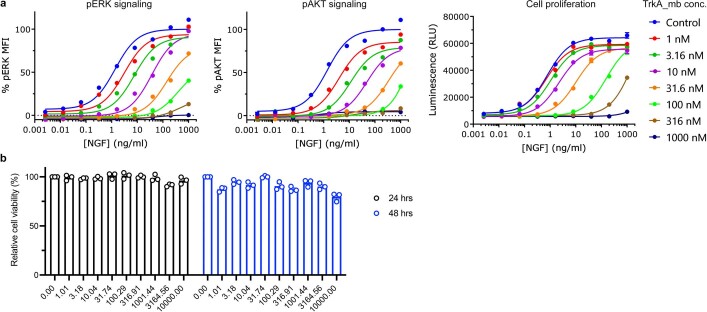
Inhibition of the TrkA miniprotein binder on the native TrkA-NGF signaling pathway. **a**, Titration curves of nerve growth factor (NGF) on TrkA signaling in the presence of different concentrations of the TrkA miniprotein binder. The TrkA miniprotein binder shifted the IC_50_ values of the TrkA response to NGF. **b**, The TrkA miniprotein binder showed no effects on the cell viability. TF-1 cells were treated with different concentrations of the TrkA miniprotein binder and the cell viability was quantified at both 24 and 48 hr. The mean values were calculated from duplicates for the pERK and pAKT signaling data, and triplicates for the cell proliferation and cell toxicity data. The error bars for the cell proliferation and cell cell toxicity data represent standard deviations.

**Extended Data Fig. 9:**
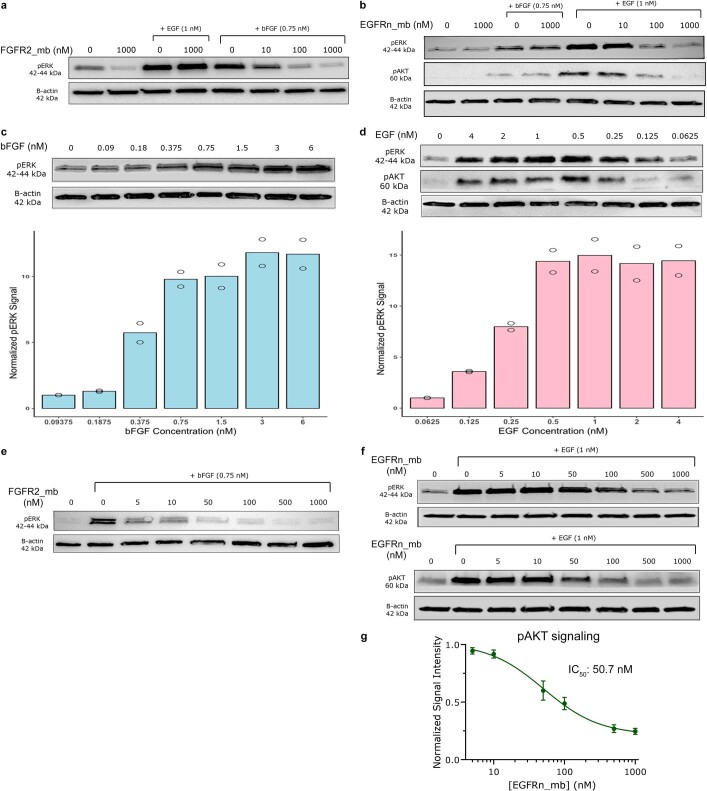
Experimental characterization of the effects of the FGFR2 minibinder and the EGFR n-side minibinder on their native signaling. **a**, FGFR2 mini binder (FGFR2_mb) inhibits FGF-induced ERK phosphorylation. Western Blot analysis showing reduction in FGF signaling (lanes 4-8) with increase in mini binder concentration. Lanes 3-4 show that EGF-induced ERK phosphorylation is unaffected by FGFR2 mini binder, eliminating any cross talk between the two receptors. **b**, EGFR n-side mini binder (EGFRn_mb) inhibits EGF-induced ERK and AKT phosphorylation. Western Blot analysis showing reduction in EGF signaling (lanes 4-8) with increase in mini binder concentration. Lanes 3-4 show that βFGF-induced ERK phosphorylation is unaffected by EGFR mini binder, eliminating any crosstalk between the two receptors. **c**, Titration curve for bFGF mediated pERK signaling. (upper) Western Blot showing dose-dependent increase in FGF signaling with βFGF concentration. (lower) n = 2 biologically independent experimental repeats were performed, and quantification was done using ImageJ analysis software. The selected concentration for competition assays was 0.75 nM. **d**, Titration curve for EGF mediated pERK/pAKT signaling. (upper) Western Blot showing dose-dependent increase in EGF signaling with EGF concentration. (lower) n = 2 biologically independent experimental repeats were performed, and quantification was done using ImageJ analysis software. The selected concentration for competition assays was 1 nM. **e**, Representative Western Blot for inhibition curves – FGFR2 minibinder. Western Blot shows dose-dependent reduction in pERK signaling with mini minder concentration. Quantification was done using ImageJ analysis software. **f**, Representative Western Blot for inhibition curves – EGFR n-side minibinder. Western Blot shows dose-dependent reduction in (upper) pERK signaling and (lower) pAKT signaling with minibinder concentration. Quantification was done using ImageJ analysis software. **g**, Dose-dependent reduction in pAKT signaling elicited by 1 nM EGF in HUVECs with increase in EGFR n-side minibinder concentration. The IC_50_ was calculated using a four-parameter-logistic equation in GraphPad Prism 9 software.

The receptor tyrosine kinases TrkA, FGFR2, PDGFR, EGFR, IR, IGF1R and TIE2 are key regulators of cellular processes and are involved in the development and progression of many types of cancer^28^. We designed binders that targeted the native ligand-binding sites for PDGFR, EGFR (on both domain I and domain III; the binders are referred to as EGFRn_mb and EGFRc_mb, respectively), IR, IGF1R and TIE2, whereas for TrkA and FGFR2, we targeted surface regions proximal to the native ligand-binding sites (Figs. 2 and 3; see Methods for criteria). We obtained binders to all eight target sites, and the binding affinities of the optimized designs ranged from about 1 nM or better for TrkA and FGFR2 to 860 nM for IGF1R (Table 1). Competition experiments with nerve growth factor (NGF), platelet-derived growth factor-BB (PDGF-BB), insulin, insulin growth factor 1 (IGF1) and angiopoietin 1 (ANG1) on yeast indicated that the binders for TrkA, PDGFR, IR, IGF1R and TIE2 bind to the targeted sites (Extended Data Fig. 7), consistent with the computational design models. The receptor tyrosine kinase binders are monomers, and as such are all expected to be antagonists. We tested the effect of the cognate binders on signalling through TrkA, FGFR2 and EGFR in cultured cells. Strong inhibition of signalling by the native agonists was observed in all three cases (Fig. 3c, and Extended Data Figs. 8 and 9).

Binding of IL-7 to the IL-7α receptor subunit leads to recruitment of the γ_c_ receptor, which forms a tripartite cytokine–receptor complex crucial to signalling cascades that lead to the development and homeostasis of T and B cells^29^. We obtained a picomolar affinity binder for IL-7Rα targeting the IL-7 binding site and found that it blocks STAT5 signalling induced by IL-7 (Fig. 3c and Table 1). We also obtained binders to CD3δ, one of the subunits of the T cell receptor, and the signalling molecule TGFβ, which play pivotal parts in immune cell development and activation (Fig. 2 and Table 1).

## Pathogen target proteins

**Extended Data Fig. 10:**
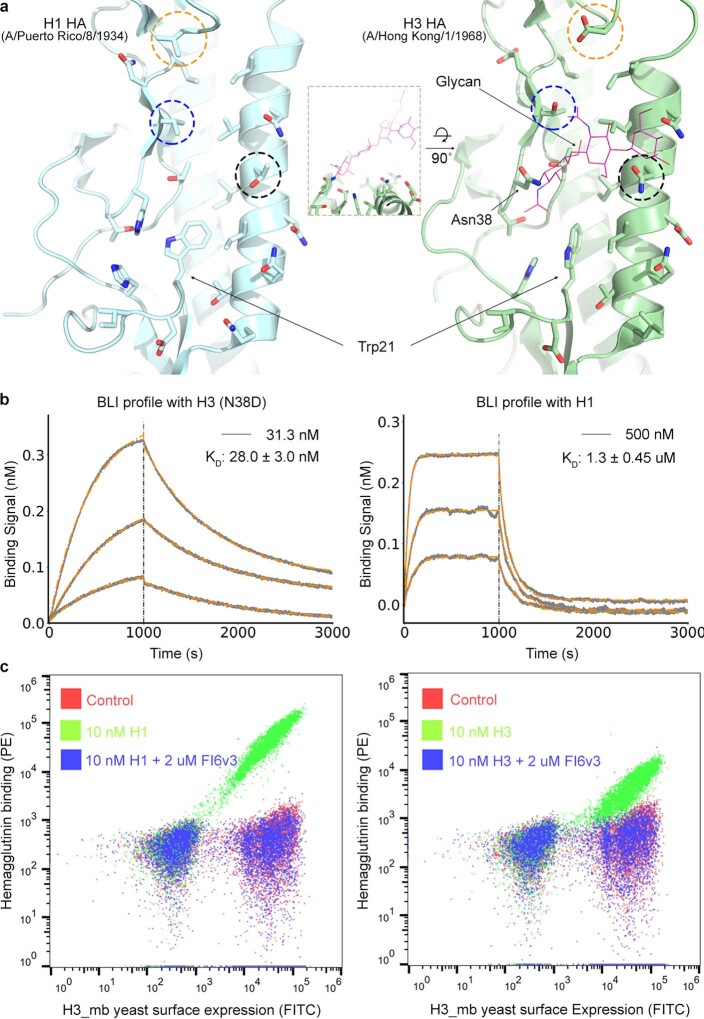
De novo design and experimental characterization of the influenza hemagglutinin (HA) binder. **a**, Structure comparison of the stem region of group 1 HA and group 2 HA. The stem regions of H1 HA (A/Puerto Rico/8/1934) (left, PDB code: 1RU7) and H3 HA (A/Hong Kong/1/1968) (right, PDB code: 4WE4) are shown in cartoon and colored in pale cyan and pale green respectively, the key residues in the stem region are shown as sticks. Three major differences make the H3 HA stem region a more challenging target for designing de novo protein binders: the H3 HA stem region contains more polar residues and is more hydrophilic. Residues in H1 HA that are hydrophobic residues or small polar residues while the corresponding residues are polar or larger polar residues are highlighted in dashed circles; Trp21 adopts different configurations in H1 HA and H3 HA, and the targeting groove in H3 HA is much shallower and less hydrophobic; the H3 HA is glycosylated at Asn38, and the carbohydrate side chains cover the hydrophobic groove and protect the HA stem region from binding by antibodies or designed binders. The insert shows a more extended view of the Asn38 glycosylation site on H3 HA. **b**, Binding of H3 binder to the H3 HA (A/Hong Kong/1/1968) N38D mutant (left) and H1 HA (A/Puerto Rico/8/1934) (right) with BLI. Two-fold serial dilutions were tested for each binder and the highest concentrations and the binder affinities are labeled. The gray color represents experimental data and orange color represents fit curves. **c**, The FI6v3 antibody competes with the binder for binding to the influenza A H1 hemagglutinin (left) and influenza A H3 hemagglutinin (right). Yeast cells displaying the H3 binder were incubated with 10 nM H1 or H3 in the presence or absence of 2 μM FI6v3 antibody, and hemagglutinin binding to cells (y axis) was monitored with flow cytometry.

Influenza haemagglutinin (HA) is the main target for influenza A virus vaccines and drugs, and can be genetically classified into two main subgroups: group 1 and group 2 (refs. ^30,31^). The HA stem region is an attractive therapeutic epitope as it is highly conserved across all influenza A subtypes, and targeting this region can block the low-pH-induced conformational rearrangements associated with membrane fusion, which is essential for virus infection^32,33^. Neutralizing antibodies that target the stem region of group 2 HA have been identified through screens of large B cell libraries after vaccination or infection that neutralize both group 1 and group 2 influenza A viruses^34,35^. Protein^1,5^, peptide^36^ and small-molecule inhibitors^37^ have been designed to bind to the stem region of group 1 HA and neutralize influenza A viruses, but none recognize the group 2 HA. The design of small proteins or peptides that can bind and neutralize both group 1 and group 2 HA has been challenging owing to three main differences between group 1 and group 2 HA. First, the group 2 HA stem region is more hydrophilic, containing more polar residues. Second, in group 2 HA, Trp21 adopts a configuration roughly perpendicular to the surface of the targeting groove, which makes the targeted groove much shallower and less hydrophobic. And third, the group 2 HA is glycosylated at Asn38, with the carbohydrate side chains covering the hydrophobic groove (Extended Data Fig. 10a). We used our interface design method to design binders to H3 HA (A/Hong Kong/1/1968), the main pandemic subtype of group 2 influenza virus, and obtained a binder with an affinity of 320 nM to wild-type H3 (Fig. 2 and Table 1) and 28 nM to the deglycosylated H3 variant (N38D) (Extended Data Fig. 10b). The reduction in affinity is probably due to entropy loss of the glycan following binding and/or steric clash with the glycan. The binder also bound H1 HA (A/Puerto Rico/8/1934), which belongs to the main pandemic subtype of group 1 influenza virus (Extended Data Fig. 10b). The binding to both H1 and H3 HA is competed by the stem region that binds the neutralizing antibody FI6v3 (ref. ^34^) on the yeast surface (Extended Data Fig. 10c), which indicates that the binder attaches the HA at the targeted site. We also designed binders to the prokaryotic pathogen protein VirB8, part of the type IV secretion system of *R. typhi*, the causative agent of murine typhus^26^. We selected the surface region composed of the second and the third helices of VirB8, and obtained binders with 510 pM affinity (Fig. 2 and Table 1).

With the outbreak of the SARS-CoV-2 pandemic, we applied our method to design miniproteins that targeted the receptor-binding domain of the SARS-CoV-2 spike protein near the ACE2 binding site to block receptor engagement. Owing to the pressing need for coronavirus therapeutics, we recently described the results of these efforts^38^ ahead of those described in this manuscript. Similar to FGFR2, IL-7Rα and VirB8, the method yielded picomolar binders, which are among the most potent compounds known to inhibit the virus in cell culture (half-maximal inhibitory concentration (IC_50_) of 0.15 ng ml^–1^). Subsequent animal experiments showed that they provide potent protection against the virus in vivo^39^. The modular nature of the miniprotein binders enables their rapid integration into designed diagnostic biosensors for both influenza and SARS-CoV-2 binders^40^.

**Fig. 4:**
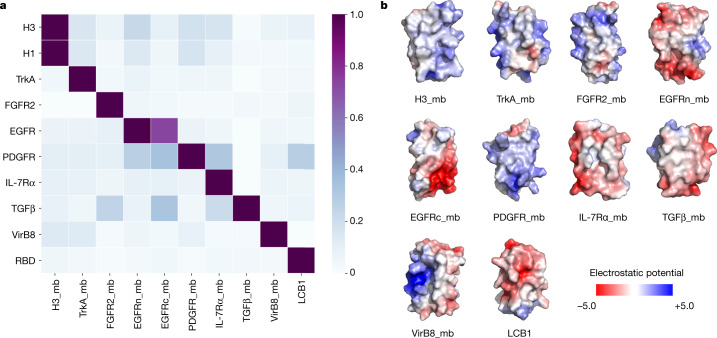
Designed binders have high target specificity. To assess the cross-reactivity of each miniprotein binder (mb) with each target protein, biotinylated target proteins were loaded onto biolayer interferometry streptavidin sensors, allowed to equilibrate and the baseline signal set to zero. The biolayer interferometry tips were then placed into 100 nM binder solution for 5 min, washed with buffer, and dissociation was monitored for an additional 10 min. The heat map shows the maximum response signal for each binder–target pair normalized by the maximum response signal of the cognate designed binder–target pair. The raw biolayer interferometry traces are shown in the Supplementary Data 1. **b**, Surface shape and electrostatic potential (generated with the APBS Electrostatics plugin in PyMOL; red positive, blue, negative) of the designed binding interfaces.

The designed binding proteins are all small proteins (<65 amino acids), and many are triple-helix bundles. To evaluate their target specificity, we tested the highest affinity binder to each target for binding to all other targets. There was little cross-reactivity (Fig. 4a), which is probably due to their diverse surface shapes and electrostatic properties (Fig. 4b). Consistent with previous observations with affibodies^41^, this result indicates that a wide variety of binding specificities can be encoded in simple helical bundles. In our approach, scaffolds are customized for each target, so the specificity arises both from the set of side chains at the binding interface and the overall shape of the interface itself.

## High-resolution structural validation

High-resolution structures are crucial for evaluating the accuracy of computational protein designs. We succeeded in obtaining crystal structures of the unbound miniprotein binders for FGFR2 and IL-7Rα, and co-crystal structures of the miniprotein binders of H3, TrkA, FGFR2, IL-7Rα and VirB8 in complex with their targets (Extended Data Table 2).

**Fig. 5:**
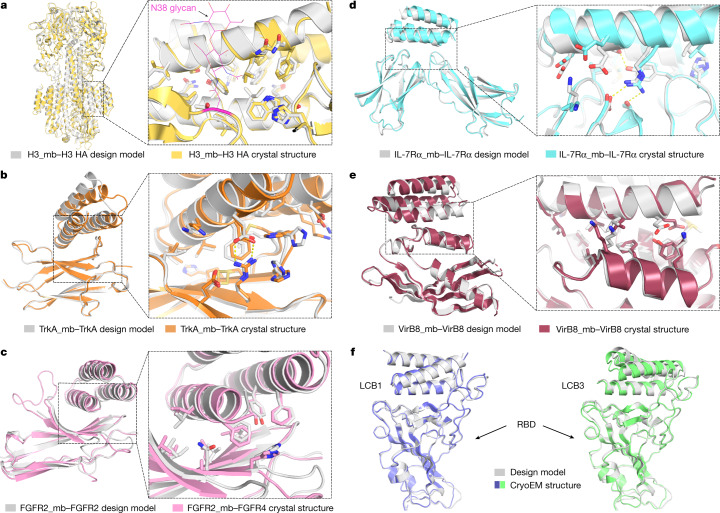
High-resolution structures of miniprotein binders in complex with target proteins closely match the computational design models. **a**–**e**, Left, superimposition of the computational design model (silver) on the experimentally determined crystal structure. Right, zoom-in view of the designed interface, with interacting side chains as sticks. **a**, H3 HA. **b**, TrkA. **c**, FGFR2. **d**, IL-7Rα. **e**, VirB8. **f**, Superimposition of the computational design model and refined cryo-EM structures of LCB1 (left) and LCB3 (right) bound to the receptor-binding domain (RBD) of the SARS-CoV-2 spike protein.

The H3 binder bound to the shallow groove of the stem region of HK68/H3 HA in the crystal structure as designed. The C_α_ root-mean-square deviation (r.m.s.d.) over the entire miniprotein binder was 1.91 Å using HA as the alignment reference (Fig. 5a). The binder makes extensive hydrophobic interactions with HA, and almost all of the designed interface side-chain configurations are recapitulated in the crystal structure (Fig. 5a). There was a clear reorientation of the oligosaccharide at Asn38 compared with the unbound HK68/H3 structure (Fig. 5a and Extended Data Fig. 10a; this has also been observed in HK68/H3 HA structures bound to stem region neutralizing antibodies^34,35^). Consistent with this result, the binder has higher affinity for the N38D variant, which lacks this glycan, than for wild-type H3 HA (A/Hong Kong/1/1968) in biolayer interferometry assays (Table 1 and Extended Data Fig. 10b).

**Extended Data Fig. 11:**
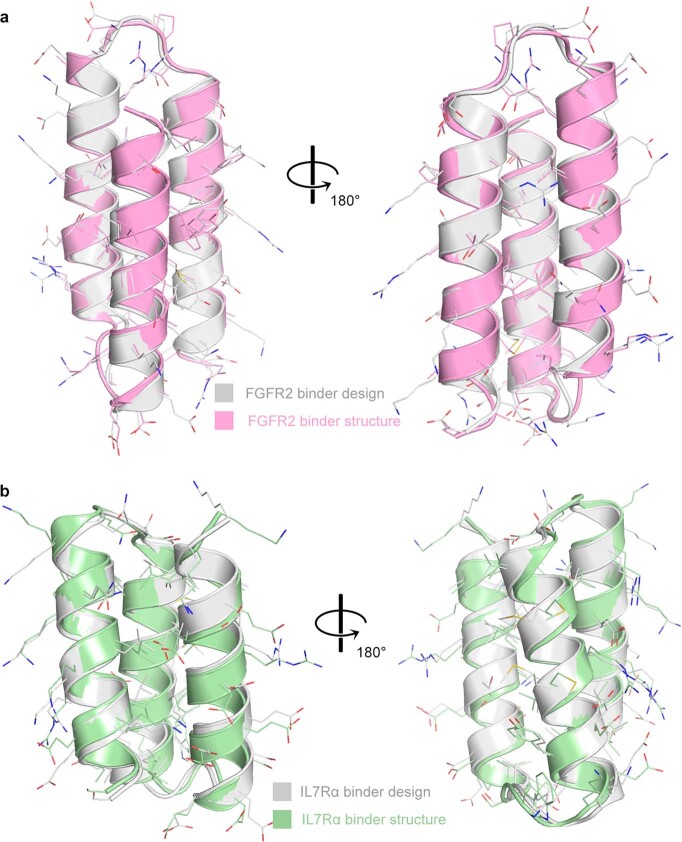
Structure characterization of the miniprotein binders without the target proteins. Superimposition of the computation of the design model (silver) and the crystal structure for the FGFR2 binder (**a**) and IL-7Rα (**b**) binder. The crystal structures of the miniprotein binders were determined without the target protein.

The crystal structure of the TrkA binder in complex with TrkA was close to the design model (Fig. 5b). After aligning the crystal structure and design model on TrkA, the C_α_ r.m.s.d. over the entire miniprotein binder was 2.41 Å, and over the two interfacial binding helices, it was 1.20 Å. The crystal structures of the FGFR2 binder by itself (Extended Data Fig. 11a) and in complex with the third immunoglobulin-like domain of FGFR4 (Fig. 5c) matched the design models with near atomic accuracy, with C_α_ r.m.s.d. values of 0.58 Å for the binder alone and 1.33 Å over the entire complex. The TrkA binder and the FGFR2 binder bound to the curved sheet side of the ligand-binding domain of TrkA and FGFR4, with extensive hydrophobic and polar interactions. Moreover, most of the key hydrophobic interactions as well as the primarily polar interactions in the computational design models were largely recapitulated in the crystal structures (Fig. 5b, c). The binding interfaces partially overlapped with the native ligand-binding sites of NGF and FGF; however, the side-chain interactions were entirely different in the designed and native complexes (Extended Data Fig. 3).

For IL-7Rα, the crystal structure of the monomer was close to that of the design, with a C_α_ r.m.s.d. of 0.63 Å (Extended Data Fig. 11b). The co-crystal structure with IL-7Rα also closely matched that of the design model, with a C_α_ r.m.s.d. of 2.2 Å using IL-7Rα as the reference (Fig. 5d). Both the de novo IL-7Rα binder and the native IL-7 use two helices to bind IL-7Rα, but the binding orientations were different (Extended Data Fig. 3). The VirB8 binder made extensive interactions with the helical regions of VirB8 as designed; no native proteins have been identified to bind to this region. The C_α_ r.m.s.d. over the entire miniprotein binder was 2.54 Å using the VirB8 as the alignment reference, and the side-chain configurations of key interface residues were largely recapitulated (Fig. 5e).

The heavy-atom r.m.s.d. values over the buried side chains at the interface (within 8 Å of the target in the design models) were 0.71 Å (H3), 1.10 Å (TrkA), 1.29 Å (FGFR2), 1.63 Å (IL-7Rα) and 1.52 Å (VirB8), all of which are close to the core side-chain r.m.s.d. values (mean 0.90 Å). Further highlighting the accuracy of the protein interface design method, cryogenic electron microscopy (cryo-EM) structures of the SARS-CoV-2 binders LCB1 and LCB3 in complex with the virus were also nearly identical to the design models, with C_α_ r.m.s.d. value of 1.27 Å and 1.9 Å, respectively^38^ (Fig. 5f).

Although we were not able yet to solve structures for the remainder of the designs, the high-resolution sequence footprinting (Figs. 2 and 3) and competition results (Extended Data Fig. 7) suggest that the interfaces involve both the designed residues and the intended regions on the target. The close agreement between the experimentally determined structures and the original design models indicates that the substitutions required to achieve high affinity play relatively subtle parts in tuning interface energetics: the overall structure of the complex, including the structure of the monomer binders and the detailed target binding mode, are determined by the computational design procedure.

## Determinants of design success

**Extended Data Fig. 12:**
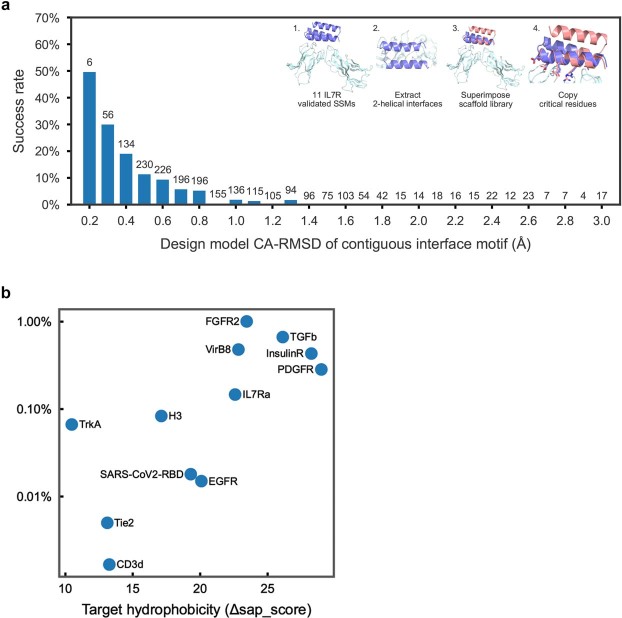
Analysis of the determinants of the success rate of de novo binder design. **a**, Correlation between success rate and root mean square deviation (RMSD) with scaffolds. In this experiment, the accuracy of the scaffold library was examined with an experiment similar to Chevalier et al^1^.. The binding residues from known-good interfaces were copied onto scaffolds that closely resembled the known-good binders. If the scaffold folded properly and displayed these binding residues similarly to the original known-good interface, the hypothesis was that the scaffold would bind. This experiment sought to determine both the required accuracy of displayed sidechains to create a successful binder as well as to probe the accuracy of the scaffold library. If for instance, the scaffold library was perfectly accurate, this graph would indicate that if the Cα RMSD of the displayed sidechains deviates from the known-good conformation by 0.5 Å, that there would be a 15% chance of binding due to the intrinsic accuracy of sidechains required for binding. The scaffold library is likely not perfectly accurate however; as such, the correct interpretation would be: If the Cα RMSD of the displayed sidechains according to the scaffold PDB model (which may not be perfectly correct) deviates by 0.5 Å Cα RMSD, there is a 15% chance of binding. This 15% chance of binding arises in part from the likelihood that the scaffold will fold correctly and in part from the intrinsic required accuracy of sidechain placements for binding. Notably, the RMSD reported in this graph is far lower than the determined crystallographic accuracy of the IL-7Rα binder when aligned by the receptor (the two interfacial helices are 1.5 Å Cα RMSD when aligned by the IL-7Rα receptor); however, if the two interfacial helices are aligned without regard for the receptor (the same calculation performed in this figure (i.e. the helices are superimposed on top of each other)) the Cα RMSD is 0.43 Å. As such, the best explanation for this data is as follows: Although the predicted binding conformation of the complex structure was only accurate to 1.5 Å, the predicted monomer structure was correct to 0.43 Å. The comparison between scaffold and known-good interface was performed at the monomer level, and therefore, these new binders were successful because they assumed the correct monomer structure, which displayed the sidechains the same as the known-good binder, and therefore were able to bind, even though the known-good complex structure was not as accurate. This graph continues to show increased signal below 0.43 Å probably because the scaffolds at very low RMSD ended up being slightly structurally different for the same reason as the known-good binder. (i.e. if we crystallized one of the scaffolds that differed only by 0.2 Å, we would likely find that scaffold model and the scaffold crystal structure deviate by about 0.43 Å and that the scaffold crystal structure and the known-good crystal structure are very similar). **Method**: 11 IL-7Rα SSM-validated interfaces were used as a starting point to create 2-helical grafts. All grafts consisted of 2-helices joined with a loop and the scaffold library was superimposed onto these two helices and the RMSD of the match was assessed. If a good match was found, the sidechains making strong interactions with IL-7Rα were copied onto the scaffold and the remaining positions near the interface were allowed to redesign to avoid clashes. Plotted on the x-axis is the RMSD of the superposition of the 2-helices + loop between the motif and the scaffold. The y-axis represents the fraction of binders with predicted SC_50_s <3 μM with the number on top representing the denominator. **b**, Target success rate versus hydrophobicity. The y-axis shows what percentage of tested binders against the indicated target showed SC_50_ below 4 μM. The x-axis shows the hydrophobicity of the target region in SAP^89^ units. A greater Δsap_score indicates greater hydrophobicity. While this graph is not completely fair as the authors improved the method with time, the trend is striking and can be used to estimate the difficulty of potential future targets. (The Δsap_score can be calculated on the target structure alone by observing the SAP score of all residues a potential binder would cover.).

For our de novo design strategy to be successful, we must encode in the approximately 60-residue designed sequences information on both the folded monomer structures and on the target binding interfaces. Indeed, designs that do not fold into the correct structure or that fold into the intended structures but do not bind to the target will fail. To assess the accuracy with which the monomer structure must be designed, we carried out an additional calculation and experiment for the IL-7Rα target. Large numbers of scaffolds were superimposed onto 11 interface helical binding motifs identified in the first broad design search, and sequence design was carried out as described above. A strong correlation was found between the extent of binding and the root mean square deviations to the binding motif (Extended Data Fig. 12a), which indicates that designed backbones must be relatively accurate to achieve binding.

**Extended Data Fig. 13:**
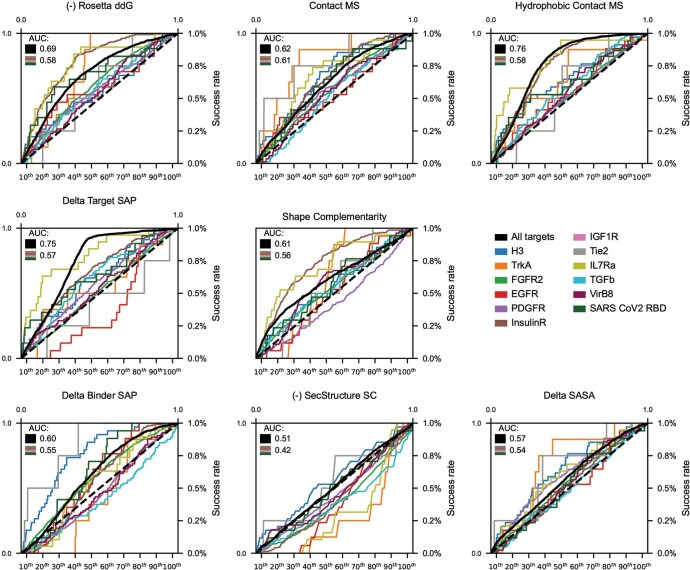
Power of computational metrics to predict binders. On the fully-relaxed binder dataset (see Methods), the ability of several computational metrics to predict which binders would have SC_50_ below 4 μM was assessed. In black and in the bar charts, data for all targets were pooled together. The bar charts show the success rate in each of the 10 percentiles for the metric while the black solid line shows the ROC plot for the metric. Each of the colored lines represents the correlation of this metric on each of the targets individually. The AUC of the overall black line is given in the upper left with the median of the AUC of the colored lines given immediately below.

To assess the determinants of binding of the designed interfaces, assuming that the designs fold into the intended monomer structures, we took advantage of the large dataset (810,000 binder designs and 240,000 single mutants) generated in this study. Design success rates varied considerably between the different targets. For some (FGFR2 and PDGFR), hundreds of binders were generated, whereas for others (TIE2 and CD3δ), fewer than 10 binders were obtained from libraries of 100,000 designs (Extended Data Table 1). Across all targets, there was a strong correlation between success rate and the hydrophobicity of the targeted region (Extended Data Fig. 12b), and designs observed experimentally to bind their targets tended to have stronger predicted binding energy and larger contact molecular surfaces (Extended Data Fig. 13). As found previously for designs of protein stability^10^, iterative design-build-test cycles in which the design method is updated at each iteration to incorporate feedback from the previous design round should lead to systematic improvements in the design methodology and success rate.

## Conclusions

Our success in designing nanomolar affinity binders for 14 target sites demonstrates that binding proteins can be designed de novo using only information on the structure of the target protein, without the need for prior information on binding hotspots or fragments from structures of complexes with binding partners. This success also suggests that our design pipeline provides a general solution to the de novo protein interface design problem that goes far beyond previously described methods. However, there is still considerable room for improvement. Only a small fraction of designs bind, and in almost all cases, the best of these require additional substitutions to achieve high-affinity binding. Furthermore, the design of binders to highly polar target sites remains a considerable challenge: the sites targeted here all contain at least four hydrophobic residues. The datasets generated in this work—both the information on binders versus non binders and the feedback on the effects of individual point mutants on binding—should help to guide the development of methods for designing high-affinity binders directly from the computer with no need for iterative experimental optimization. More generally, the de novo binder design method and the large dataset generated here provide a starting point to investigate the fundamental physical chemistry of protein–protein interactions and to develop and assess computational models of protein–protein interactions.

This work represents a major step forward towards the longer range goal of direct computational design of high-affinity binders starting from structural information alone. We anticipate that the binders created here, and new ones created with the method moving forwards, will find wide utility as signalling pathway antagonists as monomeric proteins and as tuneable agonists when rigidly scaffolded in multimeric formats, and in diagnostics and therapeutics for pathogenic disease. Unlike antibodies, the designed proteins are soluble when expressed in *E. coli* at high levels and are thermostable, and hence could form the basis for a next generation of lower cost protein therapeutics. More generally, the ability to rapidly and robustly design high-affinity binders to arbitrary protein targets could transform the many areas of biotechnology and medicine that rely on affinity reagents.

## Methods

### Broad search stage

The crystal structures of HA (Protein Data Bank (PDB) identifier: 4FNK)^25^, EGFR (PDB: 1MOX, 4UV7)^17,42^, PDGFR (PDB: 3MJG)^18^, IR (PDB: 4ZXB)^19^, IGF1R (PDB: 5U8R)^20^, TIE2 (PDB: 2GY7)^21^, IL-7Rα (PDB: 3DI3)^22^, CD3 (PDB: 1XIW)^23^, TGFβ (PDB: 3KFD)^24^ and VirB8 (PDB: 4O3V)^26^ were refined in the Rosetta energy field constrained by experimental diffraction data. The crystal structures of TrkA (PDB: 1WWW)^15^ and FGFR2 (PDB: 1EV2)^16^ were refined with the Rosetta FastRelax protocol with coordinate constraints. The targeting chain or the selected targeting region were extracted and used as the starting point for docking and design. To run PatchDock^11^, the scaffolds were mutated to poly-valine first, and default parameters were used to generate the raw docks. RifDock was used to generate the RIF by docking billions of individual disembodied amino acids to the selected targeting regions^6^. In detail, hydrophobic side-chain R-groups are docked against the target using a branch-and-bound search to quickly identify favourable interactions with the target, and polar side-chain R-groups are enumeratively sampled around every target hydrogen bond donor or acceptor. To identify backbone placements from which these interactions can be made, side-chain rotamer conformations are grown backwards for all R-group placements, and their backbone coordinates stored in a six-dimensional spatial hash table for rapid look-up. For the hierarchical searching protocol, the miniprotein scaffold library (50–65 residues in length) was docked into the field of the inverse rotamers using a branch-and-bound searching algorithm from low-resolution spatial grids to high-resolution spatial grids. For the PatchDock+RifDock protocols, the PatchDock outputs were used as seeds for the initial positioning of the scaffolds, and the docks were further refined in the finest resolution RIF. These docked conformations were further optimized to generate shape and chemically complementary interfaces using the Rosetta FastDesign protocol, activating between side-chain rotamer optimization and gradient-descent-based energy minimization. Serval improvements were added to the sequence design protocol to generate better sequences for both folding and binding. These included a better repulsive energy ramping strategy^9^, upweighting cross-interface energies, a pseudo-energy term penalizing buried unsatisfied polar atoms^13^ and a sequence profile constraint based on native protein fragments^12^. Computational metrics of the final design models were calculated using Rosetta, which includes dd*G*, shape complementary and interface buried SASA, contact molecular surface, among others, for design selection. All the script and flag files to run the programs are provided in the Supplementary Information.

### Focused search stage

The binding energy and interface metrics for all the continuous secondary structure motifs (helix, strand and loop) were calculated for the designs generated in the broad search stage. The motifs with good interactions (based on binding energy and other interface metrics, such as SASA and contact molecular surface) with the target were extracted and aligned using the target structure as the reference. All the motifs were then clustered based on an energy based-TMalign-like clustering algorithm. In brief, all the motifs were sorted on the basis of the interaction energy with the target, and the lowest energy motif in the unclustered pool was selected as the centre of the first cluster. A similar score between this motif and every motif remaining in the unclustered pool was calculated based on the TMalign algorithm^43^ without any further superimposition. Those motifs within a threshold similar score (default of 0.7) from the current cluster centre were removed from the unclustered pool and added to the new cluster. The lowest energy motif remaining in the unclustered pool was selected as the centre of the next cluster, and the second step was repeated. This process continued for subsequent clusters until no motifs remained in the unclustered pool. The best motif from each cluster was then selected based on the per-position weighted Rosetta binding energy, using the average energy across all the aligned motifs at each position as the weight. Around 2,000 best motifs were selected, and the scaffold library was superimposed onto these motifs using the MotifGraft mover^44^. Interface sequences were future optimized, and computational metrics were computed for the final optimized designs as described in the broad search stage. CPU time requirements to produce 100,000 designed binders to be tested experimentally were typically around 100,000 CPU hours (usually at least ten times as many binders were computationally designed than were ordered).

### Rapid Rosetta packing evaluation (the Predictor)

A severe speed mismatch existed between the docking methods (RifDock and focused search) and the subsequent full sequence design step. Although the docking methods can typically produce outputs every 1–3 s, the full sequence design can take upwards of 4 min. To remedy this situation, a step was designed to take about 20 s that would be more predictive than metrics evaluated on raw docks, but faster than the full sequence design.

A stripped down version of the Rosetta beta_nov16 score function was used to design only with hydrophobic amino acids. Specifically, fa_elec, lk_ball[iso,bridge,bridge_unclp], and the _intra_ terms were disabled as these proved to be the slowest energy methods by profiling. All that remained were Lennard–Jones, implicit solvation and backbone-dependent one-body energies (fa_dun, p_aa_pp, rama_prepro). Additionally, flags were used to limit the number of rotamers built at each position (Supplementary Information).

After the rapid design step, the designs were minimized twice: once with a low-repulsive score function and again with a normal-repulsive score function. Metrics of interest were then evaluated, including like Rosetta dd*G*, contact molecular surface, and contact molecular surface to critical hydrophobic residues.

Based on the observation that these predicted metrics correlated with the values after full sequence design, a maximum likelihood estimator (a functional form similar to logistic regression) was used to give each predicted design a likelihood that it should be selected to move forward. A subset of the docks to be evaluated were subjected to the full sequence design, and their final metric values calculated. With a goal threshold for each filter, each fully designed output can be marked as pass or fail for each metric independently. Then, by binning the fully designed outputs by their values from the rapid trajectory and plotting the fraction of designs that pass the goal threshold, the probability that each predicted design passes each filter can be calculated (sigmoids are fitted to smooth the distribution). From here, the probability of passing each filter may be multiplied together to arrive at the final probability of passing all filters. This final probability can then be used to rank the designs and pick the best designs to move forward to full sequence optimization.

Note that the rapid design protocol here is used merely to rank the designs, not to optimize them; the raw, non-rapid-designed docks are the structures carried forward.

### Contact molecular surface

SASA is a measure of the exposure of amino acids to the solvent and it is typically calculated using methods that involve in silico rolling of a spherical probe, which approximates a water molecule (radius 1.4 Å), around a full-atom protein model. Delta-SASA after protein–protein binding has been widely used to analyse native protein interactions. Unlike the crystal structures of the native protein complexes, design models for the de novo interactions are usually imperfectly packed and contain many holes or cavities. If the sizes of the holes or cavities in the interface are smaller than the rolling probe, SASA cannot capture those holes and cavities and the real contacts are usually overestimated by the delta-SASA metric. The contact molecular surface was developed to mitigate the flaws of the de novo designed interactions. First, the molecule surfaces of the binder and the target were calculated using the triangularization algorithm in the Rosetta shape complementary filter. For each triangle, the distance to the closest triangle on the other side was calculated and used to downweight the area of the triangle by the following equation: *A*′ = *A* × exp(−0.5 × distance^2^). Then all the downweighted areas were summed to obtain the contact molecular surface. In this way, the real contacts between the target and the binder are penalized by the cavities and holes in the interface. The contact molecular surface was implemented as the ContactMolecularSurface filter in the Rosetta macromolecular modelling suite.

### Upweighted protein interface interactions

Rosetta sequence design starts from generating an interaction graph by calculating the energies between all designable rotamer pairs^45^. The best rotamer combinations are searched using a Monte Carlo simulated annealing protocol by optimizing the total energy of the protein (monomer/complex). To obtain more contacts between the binder and the target protein, we can upweight the energies of all the cross-interface rotamer pairs by a defined factor. In this way, the Monte Carlo protocol will be biased to find solutions with better cross-interface interactions. The upweighted protein interface interaction protocol was implemented as the ProteinProteinInterfaceUpweighter task operation in the Rosetta macromolecular modelling suite.

### Comparison of sampling efficiency of PatchDock, RifDock and resampling protocols

The top 30 PatchDock outputs for the 1,000 helical scaffolds tested were designed using the RosettaScripts protocol. RifDock seeded with PatchDock outputs generated 300 outputs per scaffold, which were trimmed to a total of 19,500 docks with the Predictor (Methods) and subsequently designed. The top 150 RifDock outputs per scaffold were trimmed to 9,750, designed, and 300 motifs were extracted. The motifs were grafted into the scaffold set to produce 150,000 docks, which were trimmed to 9,750, designed, and combined with the earlier 9,750.

### DNA library preparation

All protein sequences were padded to 65 amino acids by adding a (GGGS)*n* linker at the carboxy terminus of the designs to avoid the biased amplification of short DNA fragments during PCR reactions. The protein sequences were reversed translated and optimized using DNAworks2.0 (ref. ^46^) with the *Saccharomyces cerevisiae* codon frequency table. Oligonucleotide pools encoding the de novo designs and the point mutant library were purchased from Agilent Technologies. Combinatorial libraries were purchased as Integrated DNA Technologies ultramers, with the final DNA diversity ranging from 1 × 10^6^ to 1 × 10^7^.

All libraries were amplified using Kapa HiFi polymerase (Kapa Biosystems) with a qPCR machine (Bio-Rad, CFX96). In detail, the libraries were first amplified in a 25 μl reaction, and the PCR reaction was terminated when the reaction reached half maximum yield to avoid overamplification. The PCR product was loaded onto a DNA agarose gel. The band with the expected size was cut out, and DNA fragments were extracted using QIAquick kits (Qiagen). Then, the DNA product was re-amplified as before to generate enough DNA for yeast transformation. The final PCR product was cleaned up with a QIAquick Clean up kit (Qiagen). For the yeast transformation step, 2–3 µg of linearized modified pETcon vector (pETcon3) and 6 µg of insert were transformed into the EBY100 yeast strain using a previously described protocol^47^.

DNA libraries for deep sequencing were prepared using the same PCR protocol, except the first step started from yeast plasmid prepared from 5 × 10^7^ to 1 × 10^8^ cells by Zymoprep (Zymo Research). Illumina adapters and 6-bp pool-specific barcodes were added in the second qPCR step. Gel extraction was used to obtain the final DNA product for sequencing. All the different sorting pools were sequenced using Illumina NextSeq sequencing.

### Target protein preparation

The influenza A HA ectodomain was expressed using a baculovirus expression system as previously described^25,48^. In brief, each HA was fused with a gp67 signal peptide at the amino terminus and to a BirA biotinylation site, thrombin cleavage site, trimerization domain and His-tag at the C terminus. Expressed HA was purified using metal affinity chromatography with Ni^2+^-NTA resin. For binding studies, each HA was biotinylated with BirA and purified by gel filtration using a S200 16/90 column on an ÄKTA protein purification system (GE Healthcare). The biotinylation reactions contained 100 mM Tris (pH 8.5), 10 mM magnesium acetate, 10 mM ATP, 50 µM biotin and <50 mM NaCl, and were incubated at 37 °C for 1 h.

For TrkA, the DNA encoding the human TrkA extracellular domain (ECD) (residues 36–382) was cloned into pAcBAP, a derivative of pAcGP67-A modified to include a C-terminal biotin acceptor peptide (BAP) tag (GLNDIFEAQKIEWHE) followed by a 6×His tag for affinity purification. It was then transfected into *Trichoplusia ni* (High Five) cells (Invitrogen) using the BaculoGold baculovirus expression system (BD Biosciences) for secretion and purified from the clarified supernatant through Ni-NTA followed by size-exclusion chromatography (SEC) with a Superdex-200 column in sterile PBS (Gibco, 20012-027). The ectodomains of FGFR2 (residues 147–366, UniProt ID: P21802), EGFR (residues ID 25–525, UniProt ID: P00533), PDGFR (residues 33–314, UniProt ID: P09619), IR (residues ID 28–953, UniProt ID: P06213), IGF1R (residues 31–930, UniProt ID: P08069), TIE2 (residues 23–445, UniProt ID: Q02763), IL-7Rα (residues 37–231, UniProt ID: P16871) were expressed in mammalian cells with a IgK signal peptide (METDTLLLWVLLLWVPGSTG) at the N terminus and a C-terminal tag (GSENLYFQGSHHHHHHGSGLNDIFEAQKIEWHE) that contains a TEV cleavage site, a 6-His tag and an AviTag. VirB8 was expressed in *E. coli* with a C-terminal AviTag as previously described^26^. The proteins were purified by Ni^2+^-NTA, and polished by SEC. The AviTag proteins were then biotinylated with a BirA biotin-protein ligase bulk reaction kit (Avidity) following the manufacturer’s protocol, and the excess biotin was removed through SEC. Biotinylated CD3 protein was purchased from Abcam (ab205994). TGFβ was purchased from Acro Biosystems (TG1-H8217). IGF1 was purchased from Sigma (407251-100 μg). Insulin was purchased from Abcam (ab123768). The caged ANG1-Fc protein was prepared as previously described^49^, and was provided by G. Ueda. The FI6v3 antibody was provided by D. H. Fuller (University of Washington).

### Yeast surface display

*Saccharomyces cerevisiae* EBY100 strain cultures were grown in C-Trp-Ura medium supplemented with 2% (w/v) glucose. For induction of expression, yeast cells were centrifuged at 6,000*g* for 1 min and resuspended in SGCAA medium supplemented with 0.2% (w/v) glucose at the cell density of 1 × 10^7^ cells per ml and induced at 30 °C for 16–24 h. Cells were washed with PBSF (PBS with 1% (w/v) BSA) and labelled with biotinylated targets using two labelling methods: with-avidity and without-avidity labelling. For the with-avidity method, the cells were incubated with biotinylated target, together with anti-c-Myc fluorescein isothiocyanate (FITC, Miltenyi Biotech) and streptavidin–phycoerythrin (SAPE, ThermoFisher). The concentration of SAPE in the with-avidity method was used at one-quarter of the concentration of the biotinylated targets. For the without-avidity method, the cells were first incubated with biotinylated targets, washed and secondarily labelled with SAPE and FITC. All the original libraries of de novo designs were sorted using the with-avidity method for the first few rounds of screening to exclude weak binder candidates, followed by several without-avidity sorts with different concentrations of targets. For SSM libraries, two rounds of without-avidity sorts were applied and in the third round of screening, the libraries were titrated with a series of decreasing concentrations of targets to enrich mutants with beneficial mutations. The combinatorial libraries were sorted to convergence by decreasing the target concentration with each subsequent sort and collecting only the top 0.1% of the binding population. The final sorting pools of the combinatorial libraries were plated on C-trp-ura plates, and the sequences of individual clones were determined by Sanger sequencing. The competition sort was done following the without-avidity protocols with a minor modification. In brief, the biotinylated target proteins (H1, H3, TrkA, IR, IGF1R, PDGFR and TIE2) were first incubated with an excess amount of competitors (FI6v3, FI6v3, NGF, insulin, IGF1, PDGF and caged ANG1-Fc, respectively) for 10 min, and the mixture was used for labelling the cells. The nonspecificity reagent was prepared using the protocol as previously described^50^. For the nonspecificity sort, the cells were first washed with PBSF and incubated with the nonspecificity reagent at a concentration of 100 μg ml^–1^ for 30 min. The cells were then washed and secondarily labelled with SAPE and FITC for cell sorting. The cells were then labelled with RBD using the above-described protocol.

### Miniprotein expression

Genes encoding the designed protein sequences were synthesized and cloned into modified pET-29b(+) *E. coli* plasmid expression vectors (GenScript, N-terminal 8-His tag followed by a TEV cleavage site). For all the designed proteins, the sequence of the N-terminal tag is MSHHHHHHHHSENLYFQSGGG (unless otherwise noted), which is followed immediately by the sequence of the designed protein. For proteins expressed with the maltose binding protein (MBP) tag, the corresponding genes were subcloned into a modified pET-29b(+) *E. coli* plasmid, which has a N-terminal 6-His tag and a MBP tag. Plasmids were transformed into chemically competent *E. coli* Lemo21 cells (NEB). For the designs for TrkA, FGFR2, EGFR, IR, IGF1R, TIE2, IL-7Rα, TGFβ and the MBP-tagged miniproteins, protein expression was performed using Studier autoinduction medium supplemented with antibiotic, and cultures were grown overnight. For the HA, PDGFR and CD3δ designs, the *E. coli* cells were grown in LB medium at 37 °C until the cell density reached 0.6 at OD_600_. Then, IPTG was added to a final concentration of 500 mM and the cells were grown overnight at 22 °C for expression. The cells were collected by spinning at 4,000*g* for 10 min and then resuspended in lysis buffer (300 mM NaCl, 30 mM Tris-HCL (pH 8.0), with 0.25% CHAPS for cell assay samples) with DNase and protease inhibitor tablets. The cells were lysed with a Qsonica Sonicators sonicator for 4 min in total (2 min each time, 10 s on, 10 s off) with an amplitude of 80%. The soluble fraction was clarified by centrifugation at 20,000*g* for 30 min. The soluble fraction was purified by immobilized metal affinity chromatography (Qiagen) followed by FPLC SEC (Superdex 75 10/300 GL, GE Healthcare). All protein samples were characterized by SDS–PAGE, and purity was greater than 95%. Protein concentrations were determined by absorbance at 280 nm measured with a NanoDrop spectrophotometer (Thermo Scientific) using predicted extinction coefficients.

### Circular dichroism

Far-ultraviolet circular dichroism measurements were carried out with a JASCO-1500 instrument equipped with a temperature-controlled multi-cell holder. Wavelength scans were measured from 260 to 190 nm at 25 and 95 °C and again at 25 °C after fast refolding (about 5 min). Temperature melts monitored the dichroism signal at 222 nm in steps of 2 °C min^–1^ with 30 s of equilibration time. Wavelength scans and temperature melts were performed using 0.3 mg ml^–1^ protein in PBS buffer (20 mM NaPO_4_, 150 mM NaCl, pH 7.4) with a 1 mm path-length cuvette. Melting temperatures were determined by fitting the data with a sigmoid curve equation. Nine out of the 13 designs retained more than half of the mean residue ellipticity values, which indicated that the *T*_m_ values are greater than 95 °C. *T*_m_ values of the other designs were determined as the inflection point of the fitted function.

### Biolayer interferometry

Biolayer interferometry binding data were collected on an Octet RED96 (ForteBio) and processed using the instrument’s integrated software. For minibinder binding assays, biotinylated targets were loaded onto streptavidin-coated biosensors (ForteBio) at 50 nM in binding buffer (10 mM HEPES (pH 7.4), 150 mM NaCl, 3 mM EDTA, 0.05% surfactant P20 and 1% BSA) for 6 min. Analyte proteins were diluted from concentrated stocks into the binding buffer. After baseline measurement in the binding buffer alone, the binding kinetics were monitored by dipping the biosensors in wells containing the target protein at the indicated concentration (association step) and then dipping the sensors back into baseline/buffer (dissociation). The binding affinities of TIE2 and IGF1R minibinders were low, and MBP-tagged proteins were used for the binding assay to amplify the binding signal. The binding assay for the IR designs were conducted with Amine Reactive Second-Generation (AR2G ForteBio) Biosensors with the recommended protocol. In brief, the miniproteins were immobilized onto the AR2G tips and the IR sample was used as the analyte with the indicated concentrations. Data were analysed and processed using ForteBio Data Analysis software v.9.0.0.14.

For the cross-reactivity assay, each target protein was loaded onto streptavidin tips at a concentration of 50 nM for 325 s. The tips were dipped into the miniprotein wells for 300 s (association) and then dipped into the blank buffer wells for 600 s (dissociation). The maximum raw biolayer interferometry signal binding was used as the indicator of binding strength. The maximum signal among all the miniprotein binders for a specific target was used to normalize the data for heat-map plotting.

### Crystallization and structure determination of the H3 binder in complex with HK68/H3

To prepare the H3 minibinder (H3_mb)–HK68/H3 HA complex for crystallization, a fivefold molar excess of H3_mb was mixed with about 2 mg ml^–1^ of HK68/H3 HA in 20 mM Tris (pH 8.0), 150 mM NaCl. The mixture was incubated overnight at 4 °C to facilitate formation of the complex. Saturated complexes were then purified from unbound HB_mb by gel filtration. Gel filtration fractions containing the H3_mb–HK68/H3 HA complex were concentrated to approximately 7 mg ml^–1^ in 20 mM Tris (pH 8.0) and 150 mM NaCl. Crystallization screens were set up using the sitting-drop vapour-diffusion method with our automated CrystalMation robotic system (Rigaku) at The Scripps Research Institute. Within 3–7 days, diffraction-quality crystals had grown in 0.2 M sodium thiocyanate and 20% (w/v) PEG 3350 as a precipitant. The resulting crystals were cryoprotected through the addition of 5–15% ethylene glycol, flash cooled and stored in liquid nitrogen until data collection. Diffraction data were collected at 100 K at the Stanford Synchrotron Radiation Lightsource (SSRL) beamline 12-1 and processed with HKL-2000 (ref. ^51^). Initial phases were determined by molecular replacement using Phaser^52,53^ with a HA model from PDB identifier 4FNK (apo HK68/H3 HA). Refinement was carried out in Phenix^54^, alternating with manual rebuilding and adjustment in COOT^55^. Electron-density maps were calculated using Phenix Data collection, and refinement statistics are summarized in Extended Data Table 2. The final coordinates were validated using MolProbity^56^.

### Crystal structure of TrkA in complex with the miniprotein binder

The human TrkA receptor ECD was produced in insect cells using baculovirus and prepared as previously described^57^. Hi5 cells were co-infected in shaking Fernbach flasks with baculoviruses encoding TrkA ECD and endoglycosidase H in the presence of kifunensine. Cultures were allowed to progress for 65 h before the supernatant was recovered by centrifugation. Components from the medium were precipitated by the addition of 50 mM Tris (pH 8.0), 1 mM NiCl_2_ and 5 mM CaCl_2_, and the supernatant was filtered over diatomaceous earth. The filtrate was batch-bound to Ni^2+^-NTA resin, eluted with 200 mM imidazole in HBS (HEPES-buffered saline: 10 mM HEPES (pH 7.3), 150 mM NaCl), and purified by SEC on a Superdex-75 column (Cytiva Life Sciences). To prepare the TrkA–miniprotein complex, an excess amount of miniprotein was mixed with TrkA, digested overnight at 4 °C with 1:100 (w/w) carboxypeptidases A and B, and purified by SEC.

For crystallization, the TrkA–ligand complex was concentrated to 38 mg ml^–1^ in HBS and screened in sitting-drop format using a Mosquito crystallization robot (SPT Labtech). Initial sea urchin-like crystals were obtained from the MCSG1 screen (Anatrace-Microlytic) in 0.17 M ammonium acetate, 0.085 M sodium citrate (pH 5.6), 25.5% PEG 4000 and 15% glycerol. These crystals were crushed and used to microseed the MCSG1 screen again at a ratio of 3:2:1 protein:precipitant:seed stock, resulting in single plate-like crystals grown from 0.2 M ammonium sulfate, 0.1 M bis-Tris (pH 6.5) and 25% PEG 3350. After further optimization to 0.4 M ammonium sulfate, 0.1 M bis-Tris (pH 6.2) and 20% PEG 3350, new seeds were prepared for final seeding into 0.4 M ammonium sulfate, 0.1 M bis-Tris (pH 6.2) and 16% PEG 3350.

Crystals were cryoprotected by the addition of ethylene glycol to 30% (v/v) and flash cooled in liquid nitrogen. Diffraction data to 1.84 Å resolution were collected at 100 K using an X-ray wavelength of 1.033 Å at the SSRL beamline 12-2. Crystals were assigned to space group P21 with unit cell dimensions *a* = 42.20 Å, *b* = 205.70 Å, *c* = 72.57 Å and *β* = 106.42°. Data were indexed, integrated and scaled using XDS^58,59^ and merged using Pointless and Aimless from the CCP4 suite^60–62^.

The structure was solved by molecular replacement in Phaser^52^ using separated domains of TrkA ECD (PDB accession 2IFG) and the predicted model of the ligand as search models to place two copies of the complex in the asymmetric unit. Initial rebuilding was completed with phenix.autobuild^63^ followed by iterative rounds of manual rebuilding in Coot^64^ and refinement in Phenix^65–67^. TLS parameters were chosen using TLSMD^68^, and NCS restraints were used throughout refinement^69^. The final resolution of the data was selected as 1.84 Å by comparing the results of paired refinements at 1.84, 1.90, 1.95, 2.00 and 2.05 Å resolution^70^. The final refined model included 97.26% of residues in the favoured region of the Ramachandran plot with 0.25% outliers as calculated by MolProbity^56^.

Crystallographic software used in this study was configured and installed by SBGrid^71^. Diffraction images have been deposited in the SBGrid Data Bank with the identifier 839, and the final model and reflections have been deposited in the PDB with the identifier 7N3T.

### Crystal structures of FGFR2_mb in complex with FGFR4 domain 3 and FGFR2_mb alone

cDNA of human FGFR4 domain 3 (FGFR4_D3_, amino acids S245–D355) was amplified by PCR and cloned into pET-28a(+) plasmid (Novagen). The plasmid containing FGFR4_D3_ with N-terminal hexa-histidine tag was transformed into BL21(DE3) cells. The transformed cells were grown in LB medium at 37 °C until the OD_600_ reached 0.5, induced with 1.0 mM IPTG, grown for an additional 4 h at 37 °C and collected. The bacterial cells were resuspended and lysed by sonication. FGFR4_D3_ was refolded from insoluble fractions using a previously reported procedure^16,72,73^, and purified to homogeneity using nickel affinity chromatography (Ni^2+^-NTA agarose; Qiagen) followed by SEC (Superdex 200 Increase 10/300 GL, Cytiva) equilibrated with a buffer containing 200 mM NaCl, 25 mM HEPES (pH 8.0) and 5% glycerol. The purified FGFR4_D3_ was mixed with a 1.2-fold molar excess of FGFR2_mb and subjected to another round of SEC to isolate the FGFR4_D3_–FGFR2_mb complex. Fractions containing FGFR4_D3_ bound to FGFR2_mb were pooled and concentrated to 12 mg ml^–1^ and screened for crystallization using commercially available crystallization screening kits with Mosquito Crystal liquid handler (SPT Labtech). Crystals of the FGFR4_D3_–FGFR2_mb complex were obtained with ProPlex screening solution (Molecular Dimensions) containing 0.2 M sodium chloride, 0.1 M MES pH 6.0 and 20% PEG 3,350 at 4 °C. The crystals were cryoprotected using the mother liquor supplemented with 25% glycerol before being flash-cooled in liquid nitrogen.

Crystals of FGFR2_mb were obtained using solution containing alcohols (0.02 M 1,6-hexanediol, 0.02 M 1-butanol, 0.02 M 1,2-propanediol, 0.02 M 2-propanol, 0.02 M 1,4-butanediol, 0.02 M 1,3-propanediol), buffer mixture (0.1 M Tris and BICINE adjusted to pH 8.5) and precipitants (12.5% v/v MPD, 12.5% PEG 1000, 12.5% w/v PEG 3,350) by the hanging-drop vapour-diffusion method at 20 °C, which were directly flash-cooled in liquid nitrogen for X-ray diffraction data collection.

X-ray diffraction data were collected at the NE-CAT 24ID-E beam line of Advanced Photon Source (Argonne National Laboratory) and processed with XDS^74^. The initial structure of FGFR2_mb was obtained by molecular replacement with PHASER^52,75^ using the designed model, which was iteratively refined using PHENIX^67,75^ followed by manual building with COOT^64^. The structure of FGFR4_D3_–FGFR2_mb complex was obtained by molecular replacement with Phaser^52,75^ using the coordinates corresponding to the domain 3 region of FGFR1c^72^ (PDB ID: 1CVS) and the coordinates of FGFR2_mb as the search model, followed by iterative refinements using PHENIX^67,75^ and COOT^64^. The final structures were validated with MolProbity^75,76^. Data collection and refinement statistics are provided in Extended Data Table 2.

### Crystal structure of unbound IL-7Rα minibinder

To facilitate crystallization, the N-terminal His-tag was removed using TEV protease and the protein was concentrated to 40 mg ml^–1^ in 30 mM Tris-HCl (pH 8.0) and 150 mM NaCl. Sparse-matrix crystal screening was performed using kits from Hampton Research (Index-HT, PEGRx-HT and PEG/Ion-HT) at room temperature. A Mosquito nanolitre crystallization robot was used to set up sitting drops consisting of 200 nl of protein and 200 nl of each reservoir solution with 80 μl of reservoir solution in MRC-2 plates. Promising prism-shaped crystals grew from the IndexHT C3 condition, and optimal conditions ranged from 2.4 to 3.0 M sodium malonate (pH 7.0). Protein crystals were cryo-cooled directly into liquid nitrogen. Initial X-ray diffraction experiments were carried out on a home-source system equipped with MicroMax-007 HF rotating anode with a Dectris Eiger R 4M single-photon counting device. X-ray diffraction data on optimized protein crystals were collected at the Advanced Photon Source synchrotron beamline 23ID-D of GM/CA with a Dectris Pilatus3-6M detector. All X-ray data were processed with XDS. Molecular replacement using the de novo designed model was used to solve the crystal structure using Phaser within the Phenix package. Two molecules were located in the asymmetric unit. Structural refinement used Phenix using no NCS restraints. Data collection and refinement statistics are given in Extended Data Table 2.

### Crystal structure of IL-7Rα in complex with the minibinder

The ectodomain of human IL-7Rα was produced and purified as previously described^77^. The anti-IL-7Rα minibinder was prepared as described above. The IL-7Rα–minibinder complex was formed by adding a molar excess of purified minibinder to recombinant IL-7Rα. The IL-7Rα–minibinder complex was purified by SEC using a Superdex-75 column (Cytiva Life Sciences) with HBS buffer (pH 7.4) as the running buffer. Fractions corresponding to the IL-7Rα–minibinder complex were pooled and concentrated by centrifugal ultrafiltration to a concentration of 3.9 mg ml^–1^. Sparse-matrix crystallization screens were carried out using the BCS-Screen (Molecular Dimensions) at 293 K and the sitting-drop method. The vapour-diffusion geometry was used to set up sitting drops consisting of 200 nl of protein and 100 nl of each reservoir solution using a Mosquito nanolitre crystallization robot (TTP Labtech). The IL-7Rα–minibinder complex crystallized in condition A5 (0.1 M phosphate, citrate (pH 5.5) and 25.0% PEG Smear medium). Crystals were cryo-protected with mother liquor supplemented with 25% v/v PEG 400 and cryo-cooled by direct plunging into liquid nitrogen. X-ray diffraction data of protein crystals were collected at beamline ID23-2 of the ESRF (Grenoble) with a Dectris PILATUS3 X 2M detector and were processed with XDS^58^. The structure was determined by maximum-likelihood molecular replacement in Phaser using the crystal structure of IL-7Rα (PDB ID: 3DI2) as a search model^52^. Three copies of the complex were located in the asymmetric unit. Model (re)building was performed in Coot^64^, and coordinate and ADP refinement was performed in PHENIX^65^ and autoBuster^78^. Model and map validation tools in Coot, the PHENIX suite and the PDB_REDO server^79^ were used to validate the quality of crystallographic models. The final model and reflections have been deposited in PDB with the identifier 7OPB. Data collection and refinement statistics are provided in Extended Data Table 2.

### Crystal structure of VirB8-like protein in complex with the minibinder

VirB8-like protein of the type IV secretion system from *R. typhi* (UniProt ID: Q68X84) in complex with 0.75 mM VirB8 miniprotein binder was suspended in a buffer containing 20 mM HEPES pH 7.0, 300 mM NaCl and 5% glycerol. The complex was crystallized using the sitting-drop vapour-diffusion method at 14 °C with drops composed of 0.4 ml of the complex at 9.9 mg ml^–1^ mixed with 0.4 ml crystallant (sparse matrix screen JCSG Top96 (Rigaku Reagents) condition G9: 100 mM sodium acetate/hydrochloric acid (pH 4.6), 25% (w/v) PEG 4000, 200 mM ammonium sulfate) equilibrated against 80 ml crystallant in the reservoir. Crystals were cryoprotected in the crystallant supplemented with 15% (v/v) ethylene glycol. X-ray diffraction data of the VirB8 protein–miniprotein binder complex was collected at the LS-CAT beamline 21-ID-F at the Advanced Photon Source. Data were integrated in XDS and reduced using XSCALE^58^. Data quality was assessed using POINTLESS^80^. Molecular replacement was performed using Phaser^52^ with search models comprising a previously solved crystal structure of *R. typhi* VirB8-like of type IV secretion system (PDB ID: 4O3V) and an Alphafold2 (ref. ^81^) predicted model of the VirB8 miniprotein binder. Iterative manual model building and refinement were carried out using Coot^64^ and Phenix^65^. Structure quality was assessed using Molprobity^56^ before deposition in the PDB^82,83^ (Extended Data Table 2). Diffraction images are available at the Integrated Resource for Reproducibility in Macromolecular Crystallography^84,85^.

### Comparison between the crystal structures and design models

For the structures of the miniprotein binders in complex with the targets, the entire structures were aligned using the target as the references first. The r.m.s.d. over the C_α_ atoms of the entire miniprotein binder was calculated. For the unbound crystal structures of the FGFR2 miniprotein binder and the IL-7Rα miniprotein binder, the r.m.s.d. values were calculated over all the C_α_ atoms after superimposition. For the analysis of the heavy atoms of the interface core residues, the structures were aligned using the target as references first. Interface residues of the binders were selected as long as there is one residue on the target that has a Cβ–Cβ distance of less than 8 Å using the NeighborhoodResidueSelector, and core residues were selected using the LayerSelector in Rosetta with the default burial cut-off value. Then heavy atoms of the interface core residues were used to calculate the r.m.s.d. values. Four, eight, six and six residues were considered as interface core residues for the H3, FGFR2, IL-7Rα and VirB8 complex structures respectively.

### TrkA minibinder antagonist assay

The Phospho-flow signalling assay was used to characterize the antagonistic properties of the TrkA minibinder. TF-1 cells (American Type Culture Collection, CRL-2003) were starved for 4 h in base medium without NGF or other cytokines before signalling assays. Cells were plated in 96-well plates with different concentrations of TrkA binder and stimulated with human beta-NGF (R&D) for 10 min at 37 °C, followed by fixation with 1.6% paraformaldehyde for 10 min at room temperature. Cells were permeabilized by resuspension in ice-cold methanol and stored at −20 °C until flow cytometry analysis. For intracellular staining, the permeabilized cells were washed and incubated with Alexa Fluor-488 conjugated anti-ERK1/2 pT202/pY204 antibody (BD) and Alexa Fluor-647 conjugated anti-Akt pS473 antibody (Cell Signaling Technology) for 1 h at room temperature. After washing with autoMACS running buffer (Miltenyi), the fluorescence intensity of each antibody staining level was acquired using a CytoFlex flow cytometer (Beckman Coulter). Mean fluorescence intensity (MFI) values were background subtracted and normalized to the maximal MFI value in the absence of TrkA binder and plotted in Prism 9 (GraphPad). The dose–response curves were generated using the sigmoidal dose–response analysis method.

For the cell proliferation assay, TF-1 cells were plated in a 96-well plate and cultured in RPMI-1640 medium containing 2% FBS and different concentrations of TrkA binder and NGF for 48 h at 37 °C. The cell proliferation rate was assessed by measuring the cellular ATP level using CellTiter-Glo 2.0 Cell Viability Assay reagent (Promega) according to the manufacturer’s protocol. The luminescent signal was measured using a SpectraMax Paradigm plate reader, and the data were plotted and analysed using Prism 9 (GraphPad). The dose–response curves were generated using the sigmoidal dose-response analysis method.

### FGFR2 and EGFR minibinder antagonist assay

For cell culture, human umbilical vein endothelial cells (HUVECs; Lonza, C2519AS) were grown in EGM2 medium on 35-mm cell culture dishes coated with 0.1% gelatin. In brief, EGM2 is composed of 20% FBS, 1% penicillin–streptomycin, 1% GlutaMAX (Gibco, 35050061), 1% ECGS (endothelial cell growth factor), 1 mM sodium pyruvate, 7.5 mM HEPES, 0.08 mg ml^–1^ heparin and 0.01% amphotericin B in a mixture of 1× RPMI-1640 with and without glucose (final glucose concentration = 5.6 mM). Medium was filtered through a 0.2-µm filter. HUVECs were serially passaged and expanded before cryopreservation.

### FGFR and EGFR antagonist assay

Frozen HUVECs were thawed and cultured in a 35-mm dish in EGM2 medium until confluency was reached. After that, EGM2 medium was aspirated and cells were rinsed twice with 1× PBS. Cells were then serum-starved by adding 2 ml of DMEM serum-free medium (1 g l^–1^ glucose, Gibco) for 16 h, after which the starvation medium was aspirated. The cells were then treated with the FGFR2 minibinder or the EGFR minibinder for 1 h at 37 °C and at concentrations varying between 5 nM and 1 μM of minibinder. This was followed by stimulation with β-FGF (0.75 nM, Fisher Scientific) or EGF (1 nM, Peprotech), respectively, for 15 min at 37 °C. After treatment, the medium was aspirated, and cells were washed once with 1× PBS before collecting the total protein for analysis.

### Total protein isolation

After minibinder treatment, the cells were gently rinsed in 1× PBS before lysis with 130 µl of lysis buffer containing 20 mM Tris-HCL (pH 7.5), 150 mM NaCl, 15% glycerol, 1% Triton, 3% SDS, 25 mM β-glycerophosphate, 50 mM NaF, 10 mM sodium pyrophosphate, 0.5% orthovanadate, 1% PMSF (all obtained from Sigma-Aldrich), benzonase nuclease (EMD Chemicals), protease inhibitor cocktail (Pierce protease inhibitor mini tablets, Thermo Scientific) and phosphatase inhibitor cocktail 2 (P5726). Cell lysate was collected in a fresh Eppendorf tube. A total of 43.33 µl of 4× Laemmli sample buffer (Bio-Rad) (containing 10% β-mercaptoethanol) was added to the cell lysate and then heated at 95 °C for 10 min. The boiled samples were either used for western blot analysis or stored at −80 °C.

### Western blotting

A total of 30 µl of protein lysate was loaded per well and separated on a 4–20% SDS–PAGE gel for 30 min at 250 V. Proteins were then transferred onto a nitrocellulose membrane for 12 min using a semi-dry turbo transfer apparatus (Bio-Rad). The membranes were blocked in 5% BSA for 1 h, after which they were probed overnight with respective primary antibodies on a rocker at 4 °C. The primary antibodies used in this assay were β-actin (1:10,000; Cell Signaling Technologies), p-ERK1/2 p44/42 (1:10,000; Cell Signaling Technologies) and p-AKT S473 (1:2,000; Cell Signaling Technologies). The next day, membranes were washed three times with 1× TBS-T and then incubated with anti-rabbit HRP conjugated secondary antibody (1:10,000; Bio-Rad) for 1 h. For p-AKT S473, following washes, the membrane was blocked in 5% milk at room temperature for 1 h and then incubated in the respective HRP-conjugated secondary antibody (1:2,000) prepared in 5% milk, for 1 h. They were developed using Immobilon Western chemiluminescent substrate (EMD Millipore), followed by quantification using NIH ImageJ analysis software. The raw scans of the western blot results are shown in Supplementary Fig. 5. Quantifications were done by calculating the peak area for each band. Inhibition curve fit and corresponding IC_50_ values were determined using GraphPad Prism 9 software.

### IL-7Rα minibinder antagonist assay

HEK293T cells were cultured in DMEM medium with 10% FBS at 37 °C and 5% CO_2_. Cells were co-transfected with 1,000 ng pcDNA3-γ common, 300 ng pMET7-HA-IL-7Rα, 200 ng pMX-IRES-GFP-hJak3, 300 ng empty pMET7 vector and 200 ng pGL3-b-casein-luci STAT5 reporter plasmid per well of a 6-well plate. One day after transfection, cells were detached with cell dissociation buffer (Life Technologies), re-suspended in DMEM + 10% FCS and 2% of cells were seeded in 96-well plate as previously described^77^ and stimulated overnight with 50 pM human IL-7 (Immunotools) and increasing concentrations of IL-7Rα minibinder. STAT5-dependent luciferase activity was measured on the next day using a GloMax 96 microplate luminometer. The fold-induction of luciferase activity was calculated by the ratio of the luminescence signal from cells stimulated with IL-7 to the signal from the unstimulated cells. The data were plotted and fitted to a log inhibitor versus response curve in GraphPad Prism. The pcDNA3-gamma common was a gift from J. C. Renauld (Faculty of Medicine and Dentistry, UC Louvain, Belgium) and the pMX-IRES-GFP-hJak3 vector^86^ was provided by S. N. Constantinescu (Ludwig Institute for Cancer Research, Belgium). The pMET7-HA-IL-7Rα, empty pMET7 and pGL3-β-casein-luci vectors were provided by F. Peelman (UGent, Belgium).

### Apparent SC_50_ estimation from FACS and next-generation sequencing

The Pear program^87^ was used to assemble the fastq files from the next-generation sequencing (NGS) runs. Translated, assembled reads were matched against the ordered designs to determine the number of counts for each design in each pool.

The critical assumption to the fitting here is to assume that the yeast cells displaying a particular design will follow a modified version of the standard *K*_d_ equation relating fraction bound to concentration:

1 $${\rm{F}}{\rm{r}}{\rm{a}}{\rm{c}}{\rm{t}}{\rm{i}}{\rm{o}}{\rm{n}}{\rm{\_}}{\rm{c}}{\rm{o}}{\rm{l}}{\rm{l}}{\rm{e}}{\rm{c}}{\rm{t}}{\rm{e}}{{\rm{d}}}_{i}=\frac{{\rm{c}}{\rm{o}}{\rm{n}}{\rm{c}}{\rm{e}}{\rm{n}}{\rm{t}}{\rm{r}}{\rm{a}}{\rm{t}}{\rm{i}}{\rm{o}}{\rm{n}}}{({\rm{c}}{\rm{o}}{\rm{n}}{\rm{c}}{\rm{e}}{\rm{n}}{\rm{t}}{\rm{r}}{\rm{a}}{\rm{t}}{\rm{i}}{\rm{o}}{\rm{n}}+{\rm{S}}{{\rm{C}}}_{50,i})}$$

where fraction_collected_*i*_ is the fraction of the yeast cells displaying design *i* that were collected, concentration is the target concentration for sorting, and SC_50,*i*_ is the apparent SC_50_ of the design (the concentration where 50% of the cells would be collected).

The next assumption is that all designs have the same expression level on the yeast surface and that 100% of yeast cells express sufficiently well to be collected in the ‘expression’ gate (that is, the right population in Supplementary Fig. 7).

These two assumptions, although probably false, enable fitting of the data with only one free parameter per design and no global free parameters. The correct version of equation (1) for this experiment probably has a different shape and slope from a perfect sigmoid; the net effect of correcting this would be that all SC_50_ values are scaled by a constant factor (which would not affect the relative comparisons made here). It can be shown by analysing the data that different designs result in different expression levels on yeast (one can examine the fraction collected_*i*_ for strong binders at concentrations for which binding should be saturated). The net result is that experimentally, equation (1) is multiplied by a constant between 0 and 1 for each design. This constant seems to range from 0.2 to 0.7. As such, when fitting the data, fraction collected_*i*_ values above 0.2 are considered saturating. However, because the 0.2 mark may represent 90% collection for poorly expressing designs and 30% collection for strongly expressing designs, the resulting SC_50_ fits may vary by up to fivefold. The alternative is to try to estimate an expression level; however, this becomes increasingly difficult with weaker binders that never saturate the experiment.

### Apparent SC_50_ estimation from FACS and NGS: point estimates

The following equation may be used to determine the fraction collected_*i*_ for a single design in a single sort:

2 $$\begin{array}{c}{\rm{Fraction}}{\rm{\_}}{{\rm{collected}}}_{i}=\frac{{\rm{proportion}}{\rm{\_}}{\rm{child}}{\rm{\_}}{{\rm{pool}}}_{i}}{{\rm{proportion}}{\rm{\_}}{\rm{parent}}{\rm{\_}}{{\rm{pool}}}_{i}}\\ \,\,\,\times {\rm{FACS}}{\rm{\_}}{\rm{collection}}{\rm{\_}}{\rm{fraction}}\end{array}$$

where fraction_collected_*i*_ is the proportion of cells carrying design *i* that were collected during the sort, proportion_child_pool_*i*_ is the proportion of the total NGS counts for design *i* from the pool that was collected, proportion_parent_pool_*i*_ is the proportion of the total NGS counts for design *i* from the pool that was the input for the sorter, and FACS collection fraction was the fraction of the yeast cells collected during the specific sort (a number extracted from the FACS machine itself).

This point-estimate method is best suited for asking which designs have SC_50_  < SC_50,0_ by determining the expected fraction_collected_*i*_ for a given sorting concentration and SC_50,0_. The sorting concentration and SC_50,0_ should be selected such that equation (1) results in an expected fraction_collected_*i*_ less than 0.2 to circumvent the expression issues mentioned above. Then, any designs with fraction_collected_*i*_ greater than the cut-off may say that their SC_50_ is less than SC_50,0_. Designs with low numbers of counts are suspect, see the ‘Doubly transformed yeast cells’ section below. For this analysis, any designs with fewer than max possible passenger cells were eliminated.

This method may be applied to avidity sorts; however, the resulting SC_50_ would be the SC_50_ during avidity experiments. It is unclear what the precise mathematical effect of avidity is, and as such we do not compare avidity SC_50_ values with non-avidity SC_50_ values.

### Apparent SC_50_ estimation from FACS and NGS: doubly transformed yeast cells

Doubly transformed yeast cells represent a major source of error in these experiments. Although rare, a yeast cell that contains two plasmids, one of a strong binder and one of a non-binder, will carry the non-binder plasmid through the sorting process. The net result is that the non-binder will end up with counts that track the strong binder; however, at a greatly reduced absolute number. Note that rare is a relative term here. Although the odds of any two specific plasmids being in one cell is low, in the entire pool of yeast, doubly transformed cells seem to be common.

We chose to address this issue by making the following assumption: non-binders that take advantage of a doubly transformed yeast cell do so from precisely one double-transformation event. In other words, we assumed that the same non-binding plasmid did not get doubly transformed into two separate strong-binding yeast. This assumption allows us to estimate the largest number of cells we would expect to see from a doubly transformed plasmid:

3 $$\begin{array}{c}{\rm{M}}{\rm{a}}{\rm{x}}{\rm{\_}}{\rm{p}}{\rm{o}}{\rm{s}}{\rm{s}}{\rm{i}}{\rm{b}}{\rm{l}}{\rm{e}}{\rm{\_}}{\rm{p}}{\rm{a}}{\rm{s}}{\rm{s}}{\rm{e}}{\rm{n}}{\rm{g}}{\rm{e}}{\rm{r}}{\rm{\_}}{\rm{c}}{\rm{e}}{\rm{l}}{\rm{l}}{\rm{s}}=\frac{{{\rm{c}}{\rm{e}}{\rm{l}}{\rm{l}}{\rm{s}}{\rm{\_}}{\rm{c}}{\rm{o}}{\rm{l}}{\rm{l}}{\rm{e}}{\rm{c}}{\rm{t}}{\rm{e}}{\rm{d}}}_{i{\rm{\_}}max}}{{{\rm{c}}{\rm{e}}{\rm{l}}{\rm{l}}{\rm{s}}{\rm{\_}}{\rm{s}}{\rm{o}}{\rm{r}}{\rm{t}}{\rm{e}}{\rm{d}}{\rm{\_}}{\rm{R}}1}_{i{\rm{\_}}max}}\\ \times {\rm{c}}{\rm{e}}{\rm{l}}{\rm{l}}{\rm{\_}}{\rm{c}}{\rm{o}}{\rm{p}}{\rm{i}}{\rm{e}}{\rm{s}}{\rm{\_}}{\rm{b}}{\rm{e}}{\rm{f}}{\rm{o}}{\rm{r}}{\rm{e}}{\rm{\_}}{\rm{f}}{\rm{i}}{\rm{r}}{\rm{s}}{\rm{t}}{\rm{\_}}{\rm{s}}{\rm{o}}{\rm{r}}{\rm{t}}\end{array}$$

where max_possible_passenger_cells is the highest number of cells that we would expect a non-binding plasmid to occupy, cells_collected_*i*_max_ is the number of cells collected in this round for the design with the greatest number of cells collected, cells_sorted_R1_*i*_max_ is the number of cells sorted for design *i*_max (the same design from cells_collected_*i*_max_), and cell copies before first sort is the number of copies of each cell that occurred before the first sort (2^no. of cell divisions^). The number of cells_collected_*i*_ may be approximated by multiplying the number of cells the FACS machine collected by the proportion of the pool that design *i* represents. The number of cells_sorted_*i*_ may be estimated by either dividing the cells_collected_*i*_ by the FACS_collection_fraction or by multiplying the number of cells fed to the FACS machine by the proportion of design *i* in that pool.

With this number in hand, one can set a floor for the number of cells that one would expect to see. Any design with fewer than this number of cells cannot be considered for calculations because it is unclear whether or not that cell is part of a doubly transformed yeast cell. On the whole, this method reduces false-positive binders but also removes true-positive binders that did not transform well. It is wise to simply drop designs from the downstream calculations that did not transform well.

### Apparent SC_50_ estimation from FACS and NGS: full estimate

Estimation of an upper and lower bound on the SC_50_ from the data may be performed by looking at an arbitrary number of sorting experiments. Taking a *P*(SC_50_ == SC_50,0_ | data) and performing Bayesian analysis, one arrives at a confidence interval for the actual SC_50_ value. This analysis may be performed at every sort and the resulting distributions combined to produce a robust estimate.

Each sort may be modelled as a binomial distribution where *P* = fraction_collected from equation (1) using concentration = sorting_concentration and SC_50_ = SC_50,0_; *n* = cells_sorted_*i*_; and *x* = cells_collected_*i*_. By performing this analysis at a range of SC_50,0_ values and examining the probability this could happen by the binomial distribution, one arrives at *P*(SC_50_ == SC_50,0_ | data). Specifically for this analysis, the cumulative distribution function (CDF) of the binomial was used with the null hypothesis that SC_50_ == SC_50,0_.

Care should be taken for the valid range of *P*. As stated previously, it is wise to cap the expected value of *P* to 0.2 to account for expression levels and to floor the value such that *n* × *P* does not fall below max possible passenger cells. In our implementation, if *x* falls into a range that has been clipped, a probability of 1 is returned.

The code to perform this entire analysis is available in the Supplementary Information.

### SSM validation: relax protocol

To remove artefacts from designs and to discover the best orientation for each SSM mutation, all binders were relaxed using the Rosetta beta_nov16 score function before calculations began (30 replicates using 5 repeats of cartesian FastRelax taking the best scoring model). Relaxation of point mutants then used the standard cartesian FastRelax procedure and allowed all residues within 10 Å of the mutation to relax. The backbone coordinates of those residues on the binder were allowed to relax while the target was held constant. The best of three (as evaluated by Rosetta energy) was chosen as the representative model. An xml is provided in the Supplementary Information to perform this relaxation.

### SSM validation: entropy score

To validate that the designed binder was folded into the correct shape and was using its designed interface to bind to the target, the entropy of the interface, monomer core and monomer surface were examined. For each position on the binder, the sequence entropy (Shannon entropy) of each position was calculated using the observed frequencies of each amino acid in the NGS. The specific pool that was chosen for this analysis was the pool with concentration closest to tenfold lower than the calculated SC_50_ of the parent.

After the per-position sequence entropy was calculated, the average per-position entropy of the SASA-hidden positions contacting the target (interface core), the SASA-hidden positions not contacting the target (monomer core) and the fully exposed positions not contacting the target (monomer surface) were calculated. A simple subtraction was performed according to equation (4):

4 $${\rm{I}}{\rm{n}}{\rm{t}}{\rm{e}}{\rm{r}}{\rm{m}}{\rm{e}}{\rm{d}}{\rm{i}}{\rm{a}}{\rm{t}}{\rm{e}}\,{\rm{e}}{\rm{n}}{\rm{t}}{\rm{r}}{\rm{o}}{\rm{p}}{\rm{y}}\,{\rm{s}}{\rm{c}}{\rm{o}}{\rm{r}}{\rm{e}}={S}_{{\rm{m}}{\rm{o}}{\rm{n}}{\rm{o}}{\rm{m}}{\rm{e}}{\rm{r}}{\rm{\_}}{\rm{c}}{\rm{o}}{\rm{r}}{\rm{e}}}+{S}_{{\rm{i}}{\rm{n}}{\rm{t}}{\rm{e}}{\rm{r}}{\rm{f}}{\rm{a}}{\rm{c}}{\rm{e}}{\rm{\_}}{\rm{c}}{\rm{o}}{\rm{r}}{\rm{e}}}-{S}_{{\rm{m}}{\rm{o}}{\rm{n}}{\rm{o}}{\rm{m}}{\rm{e}}{\rm{r}}{\rm{\_}}{\rm{s}}{\rm{u}}{\rm{r}}{\rm{f}}{\rm{a}}{\rm{c}}{\rm{e}}}$$

where *S*_region_ is the average entropy of that region.

Finally, the probability that the score could have come from totally random data was computed by performing the above calculation on the actual data, and then performing the same calculation 100 times, but randomly mismatching the observed counts among all SSM point mutations. In this way, the experimental noise is kept constant among the 100 decoy datasets. The final step to arrive at a *P* value was to calculate the mean and standard deviation of the 100 decoy intermediate entropy scores and to find the *P* value with the Normal CDF function of the binder’s intermediate entropy score.

### SSM validation: Rosetta accuracy score

To further assess the accuracy of the design model, the correlation between the predicted effect on binding by Rosetta was compared with the experimental data. The effect from Rosetta can be broken into two components: monomer stabilization/destabilization and interface stabilization/destabilization. The effect on the monomer energy will affect the fraction of the proteins that are folded in solution. This fraction of folded proteins will then worsen the affinity because only the folded proteins are able to bind. The effect on the monomer stability was estimated by taking the difference in Rosetta energy between the native relaxed dock and the mutant relaxed dock and looking only at the change in Rosetta score of the docked protein (excluding energies arising from cross-interface edges). The effect on the target energy was calculated the same and was considered to directly affect the binding energy. The binding energy was calculated by taking the difference in Rosetta score between the docked and undocked conformations (but with no repacking or minimization in the unbound form). An xml exists in the Supplementary Information to perform this calculation.

The effect on the *P*(fold monomer) was estimated by first determining the predicted Δ*G*_fold_ of the native protein.

5 $$P({\rm{fold\; monomer}})={\rm{\exp }}(\frac{\Delta {G}_{{\rm{fold}}}+\Delta {G}_{{{\rm{mutant}}}_{{\rm{effect}}}}}{{kT}})$$

6 $$\Delta {\rm{dd}}{G}_{{\rm{monomer\; effect}}}={kT}{\rm{ln}}(\frac{P({\rm{fold\; monomer}}{)}_{{\rm{native}}}}{P({\rm{fold\; monomer}}{)}_{{\rm{mutant}}}})$$

Where *k* is the Boltzmann constant and *T* is temperature, which was set to 300 K for this calculation.

Using equations (5) and (6), the predicted Δ*G*_fold_ for the native design was estimated by performing a least-squares fit of all mutations that did not occur in residues at the interface. A rudimentary confidence interval was created by allowing all Δ*G*_fold_ values that resulted in a root mean squared error of within 0.25 kcal mol^–1^ of the best Δ*G*_fold_ value. Typical confidence intervals spanned 3 kcal mol^–1^.

7 $$\Delta {\rm{dd}}{G}_{{\rm{R}}{\rm{osetta}}}=\Delta {\rm{dd}}{G}_{{\rm{monomer\; effect}}}+\Delta {\rm{dd}}{G}_{{\rm{i}}{\rm{nterface\; effect}}}+\Delta {\rm{dd}}{G}_{{\rm{target\; effect}}}$$

With the Δ*G*_fold_ in hand, the predicted effect on the binding energy could be computed according to equation (7). The values of Δ*G*_fold_ inside the confidence range for Δ*G*_fold_ that produced the largest and smallest Δdd*G*_Rosetta_ were used to produce a confidence interval for Δdd*G*_Rosetta_.

The per-position accuracy was assessed by determining whether the confidence interval for Δdd*G*_Rosetta_ was compatible with the confidence interval for the SC_50_ from the experimental data. A buffer of 1 kcal mol^–1^ was allowed.

With the per-position accuracies in hand, the overall percentage of mutations that Rosetta was able to explain in the monomer core and interface core was assessed. This produced an overall Rosetta accuracy score.

In the same way as the entropy score, 100 decoys with randomly shuffled SC_50_ values were subjected to the same procedure. The mean and standard deviation of the decoys was determined and the *P* value for the Rosetta score was determined using the Normal CDF function.

### Reporting summary

Further information on research design is available in the Nature Research Reporting Summary linked to this paper.

## Online content

Any methods, additional references, Nature Research reporting summaries, source data, extended data, supplementary information, acknowledgements, peer review information; details of author contributions and competing interests; and statements of data and code availability are available at 10.1038/s41586-022-04654-9.

### Supplementary information

Supplementary Information Supplementary Figs. 1–8, Supplementary Table 1 and information for downloading the raw design models and design scripts.

Reporting Summary

Supplementary Code **Design scripts and main pdb files**. This compressed file is the main supplement and contains the following files: => cao_2021_protocol/ => design_models_final_combo_optimized/ => design_models_sequence/ => design_models_ssm_natives/ => ngs_analysis_scripts/

Supplementary Data 1 **Experimental data and analysis**. This file contains all the experimental results and the analysis protocols.

Supplementary Data 2 **Computational protocol for data analysis**. This file contains all the computational analysis we did for Fig. 1 and the Supplementary Figs. There are no experimental data here.

## Figures and Tables

**Extended Data Table 1:**
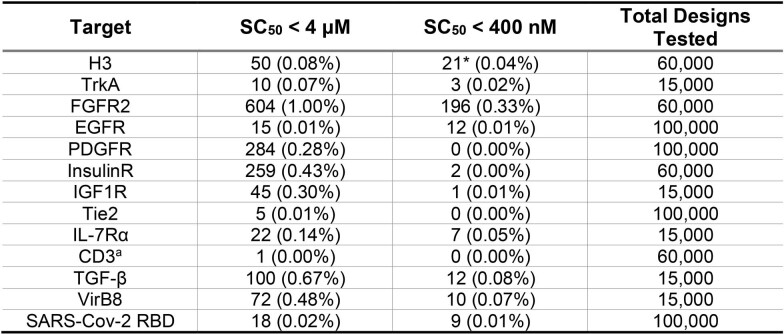
Number of binders against the 12 targets as estimated from FACS sorting

SC_50_ (Sorting Concentration_50_) refers to the target concentration where 50% of expressing yeast cells for a given design are collected. The “SC_50_ < 4 μM” column was produced by looking for binders that saw > 20% collection frequency during a 1 μM w/o avidity sort (see Method). When a 1 μM sort was not performed, 500 nM and 11% were used instead. A similar procedure was used to estimate the 400 nM column. Some binders saturate their binding signal at 20% collection frequency (likely expression problems), for this reason, the H3 data were estimated at 800nM to avoid needing a threshold higher than 20%. Additionally, binders with very low counts were discarded to guard against doubly-transformed yeast (see Methods).

^*^Number of binders with SC_50_ < 800 nM estimated from 200nM sort.

^a^ SSM sorts used to estimate the number of binders.

**Extended Data Table 2:**
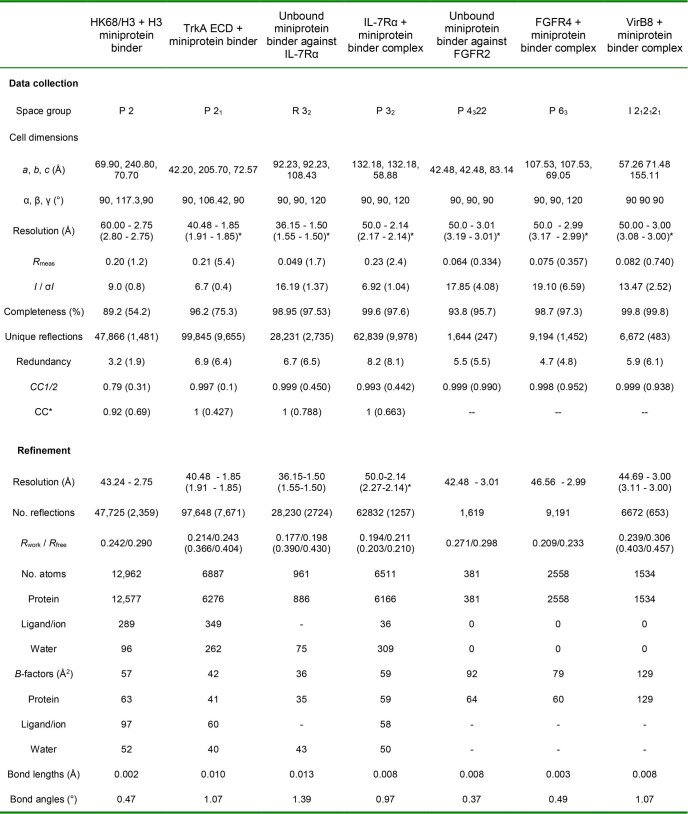
Crystallographic data collection and refinement statistics

*Data collected from a single crystal. *Values in parentheses are for the highest-resolution shell.

## Data Availability

The atomic coordinates and experimental data of H3_mb in complex with H3 HA, TrkA_mb in complex with TrkA, unbound FGFR2_mb, FGFR2_mb in complex with FGFR4, unbound IL-7Rα_mb, IL-7Rα_mb in complex with IL-7Rα and VirB8_mb in complex with VirB8 have been deposited in the RCSB PDB with the accession numbers 7RDH, 7N3T, 7N1K, 7N1J, 7S5B, 7OPB and 7SH3, respectively. Diffraction images for the TrkA–minibinder complex have been deposited in the SBGrid Data Bank with the identifier 838. The Rosetta macromolecular modelling suite (https://www.rosettacommons.org) is freely available to academic and non-commercial users. Commercial licences for the suite are available through the University of Washington Technology Transfer Office.

## Code availability

The Rosetta macromolecular modelling suite (https://www.rosettacommons.org) is freely available to academic and non-commercial users. Commercial licences for the suite are available through the University of Washington Technology Transfer Office. The design scripts and main PDB models, computational protocol for data analysis, experimental data and analysis scripts, the entire miniprotein scaffold library, all the design models and NGS results used in this paper can be downloaded from file servers hosted by the Institute for Protein Design: https://files.ipd.uw.edu/pub/robust_de_novo_design_minibinders_2021/supplemental_files/scripts_and_main_pdbs.tar.gz, https://files.ipd.uw.edu/pub/robust_de_novo_design_minibinders_2021/supplemental_files/computational_protocol_analysis.tar.gz, https://files.ipd.uw.edu/pub/robust_de_novo_design_minibinders_2021/supplemental_files/experimental_data_and_analysis.tar.gz, https://files.ipd.uw.edu/pub/robust_de_novo_design_minibinders_2021/supplemental_files/scaffolds.tar.gz, https://files.ipd.uw.edu/pub/robust_de_novo_design_minibinders_2021/supplemental_files/design_models_pdb.tar.gz and https://files.ipd.uw.edu/pub/robust_de_novo_design_minibinders_2021/supplemental_files/design_models_silent.tar.gz. All the files are stored in compressed gzip format. Once the files have been downloaded and decompressed, there is a detailed description of the binder design pipeline and the whole process can be reproduced based on those files. The source code for RIF docking implementation is freely available at https://github.com/rifdock/rifdock.
