# Supplementary material for: Design of protein-binding proteins from the target structure alone
Source: Nature. 2022 Mar 24;605(7910):551–60. doi: 10.1038/s41586-022-04654-9 (PMC9117152; doi:10.1038/s41586-022-04654-9)
Supplement: Supplementary file 1 — Supplementary Figs. 1–8, Supplementary Table 1 and information for downloading the raw design models and design scripts. [file 41586_2022_4654_MOESM1_ESM.pdf]

nature portfolio

https://doi.org/10.1038/s41586-022-04654-9

Supplementary information

Design of protein-binding proteins from the target structure alone

In the format provided by the authors and unedited

Supplementary information for “Robust de novo design of protein binding proteins from target structural information alone”

Longxing Cao[1,2,#], Brian Coventry[1,2,3,#], Inna Goreshnik[1,2], Buwei Huang[1,2,4], Joon Sung Park[5], Kevin M. Jude[6,7,8], Iva Marković[9,10], Rameshwar U. Kadam[11], Koen H.G. Verschueren[9,10], Kenneth Verstraete[9,10], Scott Thomas Russell Walsh[12,13], Nathaniel Bennett[1,2,3], Ashish Phal[1,4,20], Aerin Yang[6,7,8], Lisa Kozodoy[1,2], Michelle DeWitt[1,2], Lora Picton[6,7,8], Lauren Miller[1,2], Eva-Maria Strauch[14], Nicholas D. DeBouver[15,16], Allison Pires[16,17], Asim K Bera[1,2], Samer Halabiya[18], Bradley Hammerson[16], Wei Yang[1,2], Steffen Bernard[11], Lance Stewart[1,2], Ian A. Wilson[11,19], Hannele Ruohola-Baker[1,20], Joseph Schlessinger[5], Sangwon Lee[5], Savvas N. Savvides[9,10], K. Christopher Garcia[6,7,8], David Baker[1,2,21*]

1. Department of Biochemistry, University of Washington, Seattle, WA 98195, USA.
2. Institute for Protein Design, University of Washington, Seattle, WA 98195, USA.
3. Molecular Engineering Graduate Program, University of Washington, Seattle, WA 98195, USA.
4. Department of Bioengineering, University of Washington, Seattle, WA, 98195, USA.
5. Department of Pharmacology, Yale University School of Medicine, New Haven, CT 06520, USA.
6. Howard Hughes Medical Institute, Stanford University School of Medicine, Stanford, CA 94305, USA.
7. Department of Structural Biology, Stanford University School of Medicine, Stanford, CA 94305, USA.
8. Department of Molecular and Cellular Physiology, Stanford University School of Medicine, Stanford, CA 94305, USA.
9. VIB-UGent Center for Inflammation Research, 9052 Ghent, Belgium
10. Unit for Structural Biology, Department of Biochemistry and Microbiology, Ghent University, 9052 Ghent, Belgium.
11. Department of Integrative Structural and Computational Biology, The Scripps Research Institute, La Jolla, CA 92037, USA.
12. National Cancer Institute, National Institutes of Health, Chemical Biology Laboratory, 1050 Boyles Street, Building 376, Frederick, MD 21702
13. Present address: J.A.M.E.S. Farm, 13615 Highland Road, Clarksville, MD 21029, USA
14. Dept. of Pharmaceutical and Biomedical Sciences, University of Georgia, Athens, GA 30602, USA
15. UCB Pharma., 7869 NE Day Road West, Bainbridge Island, WA 98110, USA
16. Seattle Structural Genomics Center for Infectious Disease (SSGCID), Seattle, WA, USA
17. Seattle Children's Center for Global Infectious Disease Research, Seattle, WA, USA
18. Department of Electrical and Computer Engineering, University of Washington, Seattle, WA 98195, United States.
19. The Skaggs Institute for Chemical Biology, The Scripps Research Institute, La Jolla, CA 92037, USA.
20. Institute for Stem Cell and Regenerative Medicine, University of Washington, Seattle, WA 98109, USA
21. Howard Hughes Medical Institute, University of Washington, Seattle, WA 98195, USA

[#]These authors contributed equally
***Corresponding author. E-mail: dabaker@uw.edu**

The supplementary pdf
Supplementary Figs 1-8, Supplementary Table 1 and Information for downloading the raw design models and design scripts

Design scripts and main pdb files
Size: 61 MB
http://files.ipd.uw.edu/pub/robust_de_novo_design_minibinders_2021/supplemental_files/scripts_and_main_pdbs.tar.gz
This file is the main supplement. This compressed file contains the following files:
=> cao_2021_protocol/
=> design_models_final_combo_optimized/
=> design_models_sequence/
=> design_models_ssm_natives/
=> ngs_analysis_scripts/

Experimental data and analysis
Size: 234 MB
http://files.ipd.uw.edu/pub/robust_de_novo_design_minibinders_2021/supplemental_files/experimental_data_and_analysis.tar.gz
This file contains all the experimental results and the analysis protocols.

Computational protocol for data analysis
Size: 69 MB
http://files.ipd.uw.edu/pub/robust_de_novo_design_minibinders_2021/supplemental_files/computational_protocol_analysis.tar.gz
This file contains all the computational analysis we did for Fig1 and SFigs. There is no experimental data here.

Miniprotein scaffolds
Size: 1.3 GB
http://files.ipd.uw.edu/pub/robust_de_novo_design_minibinders_2021/supplemental_files/scaffolds.tar.gz
This file contains all the scaffolds we used in this work.

All the design models in pdb.gz format
Size: 64GB
http://files.ipd.uw.edu/pub/robust_de_novo_design_minibinders_2021/supplemental_files/design_models_pdb.tar.gz
This file contains all the design models ordered in pdb.gz format and there are more in 1 million files in total. If you are on an academic network, you may substitute files.ipd with research-files.ipd . This uses the academic internet to give you faster download speeds.

All the design models in silent format
Size: 46GB
http://files.ipd.uw.edu/pub/robust_de_novo_design_minibinders_2021/supplemental_files/design_models_silent.tar.gz
This file contains all ordered designs in Rosetta binary silent format. If you're using Rosetta, it's worth your time to figure out how to load these. These will load 10x faster than the pdb files. (And the .tar.gz only contains 30 files). See also https://github.com/bcov77/silent_tools for how to deal with silent files.

Figures

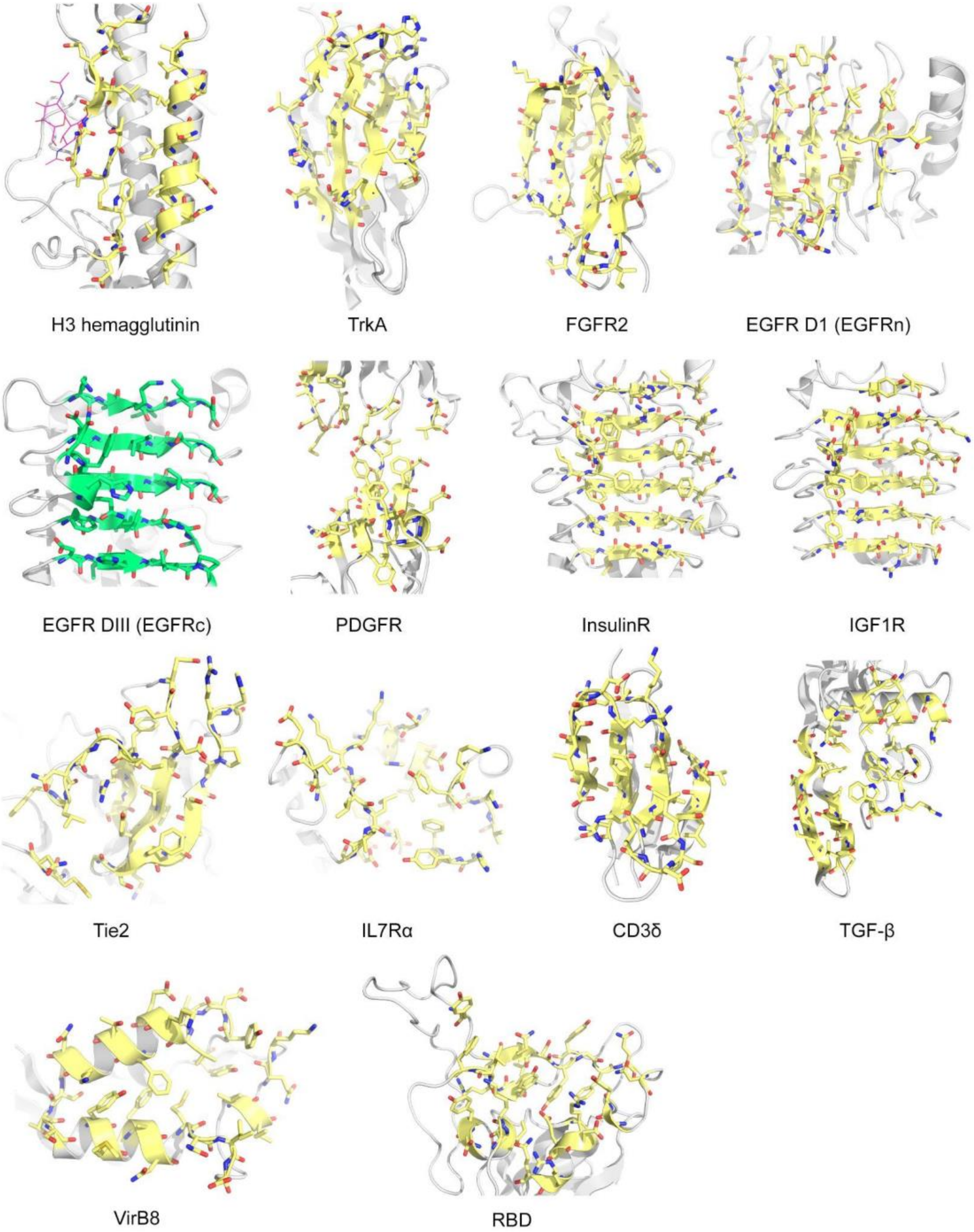

Figure 1. Zoomed in view of all of the selected targeting regions for each target. These targeting regions (pale yellow or pale green) span a wide range of surface properties, with diverse shape and chemical characteristics. We picked two different binding sites for EGFR, one colored in pale yellow and the other colored in pale green.

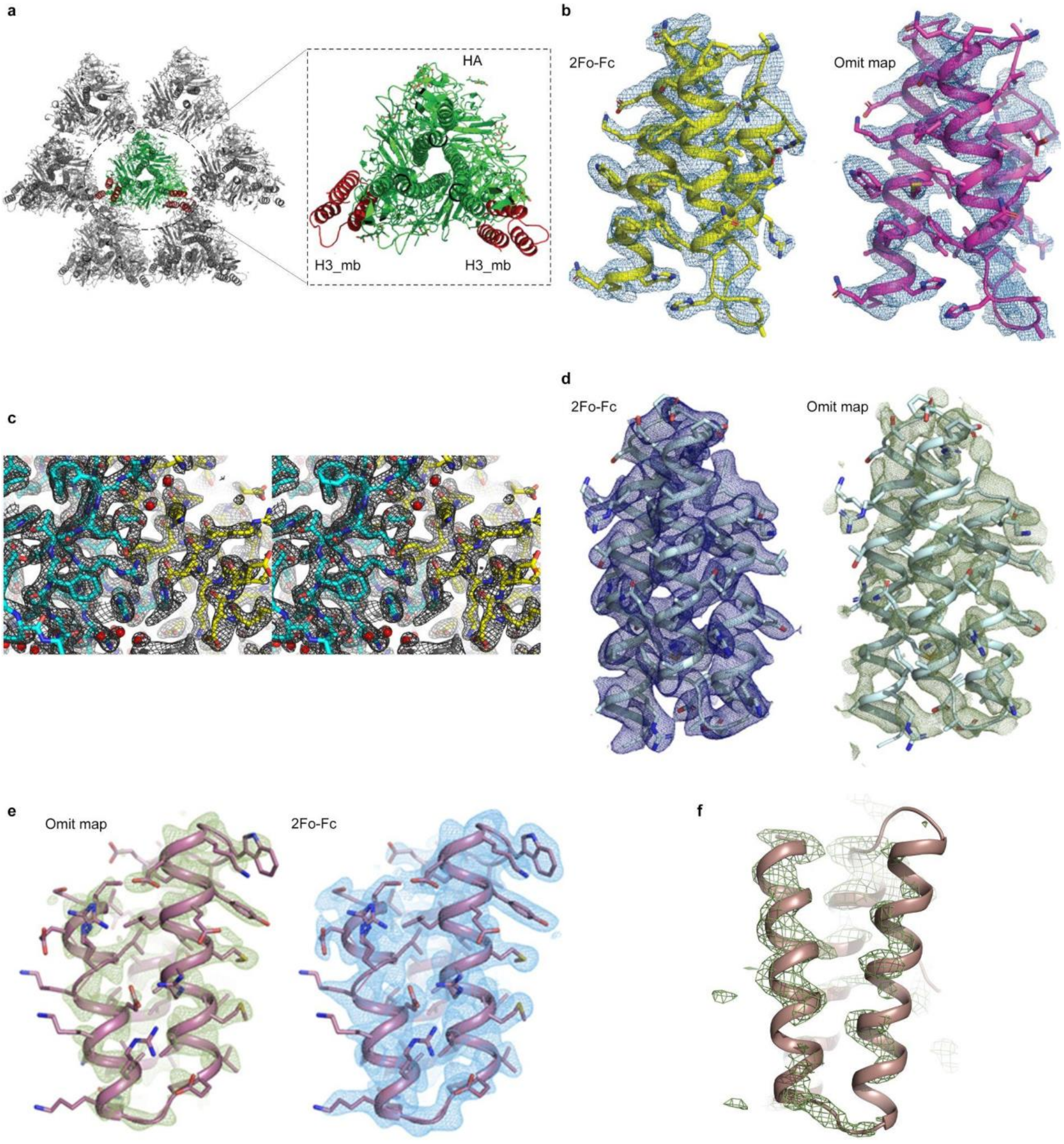

Figure 2. Crystal structure characterization of the de novo miniprotein binders in complex with the corresponding targets. **a**, Structural analysis of H3_mb/H3 HA complex revealed that the asymmetric unit contained an H3 HA trimer bound to two copies of mini protein H3_mb. The HA trimer is shown in green and gray and H3_mb in red and gray cartoon representations. **b**, Electron density maps for H3_mb in the crystal structure with H3 HA. 2Fo-Fc and simulated annealing omit maps for one of the H3_mb mini-proteins bound to H3HA are contoured at 1σ. For the 2Fo-Fc map, C/O/N/S are

represented in yellow/red/blue/dark yellow sticks and for the omit map, C/O/N/S are represented in magenta/red/blue/yellow sticks, respectively. Electron density maps are represented in a skyblue mesh. The other H3_mb in the complex has corresponding electron density. **c**, Walleye stereo view of TrkA (cyan) interface with minibinder (yellow). Simulated annealing composite omit map is contoured at 1.5 s. Symmetry-related proteins are colored gray. **d**, Structure validation of the FGFR4 in complex with the binder. 2mFo-DFc map (left) and mFo-DFc simulated-annealing omit map (right) for the miniprotein binder in complex with FGFR4 (PDB ID: 7N1J). The maps are contoured at 1σ. **e**, Structure validation of the IL-7Ra in complex with the binder. mFo-DFc simulated-annealing omit map (left) and the corresponding 2mFo-DFc (right) difference electron density map following refinement for the designed IL-7Ra binder in pdb 7OPB (chain E). The omit difference map is contoured at an r.m.s.d.-value of +3 (carve radius = 5 Å) and the 2mFo-DFc map is contoured at an r.m.s.d.-value of 1.0 (carve radius = 2 Å). **f**, mFo-DFc simulated-annealing omit map of the VirB8 binder in the complex structure.

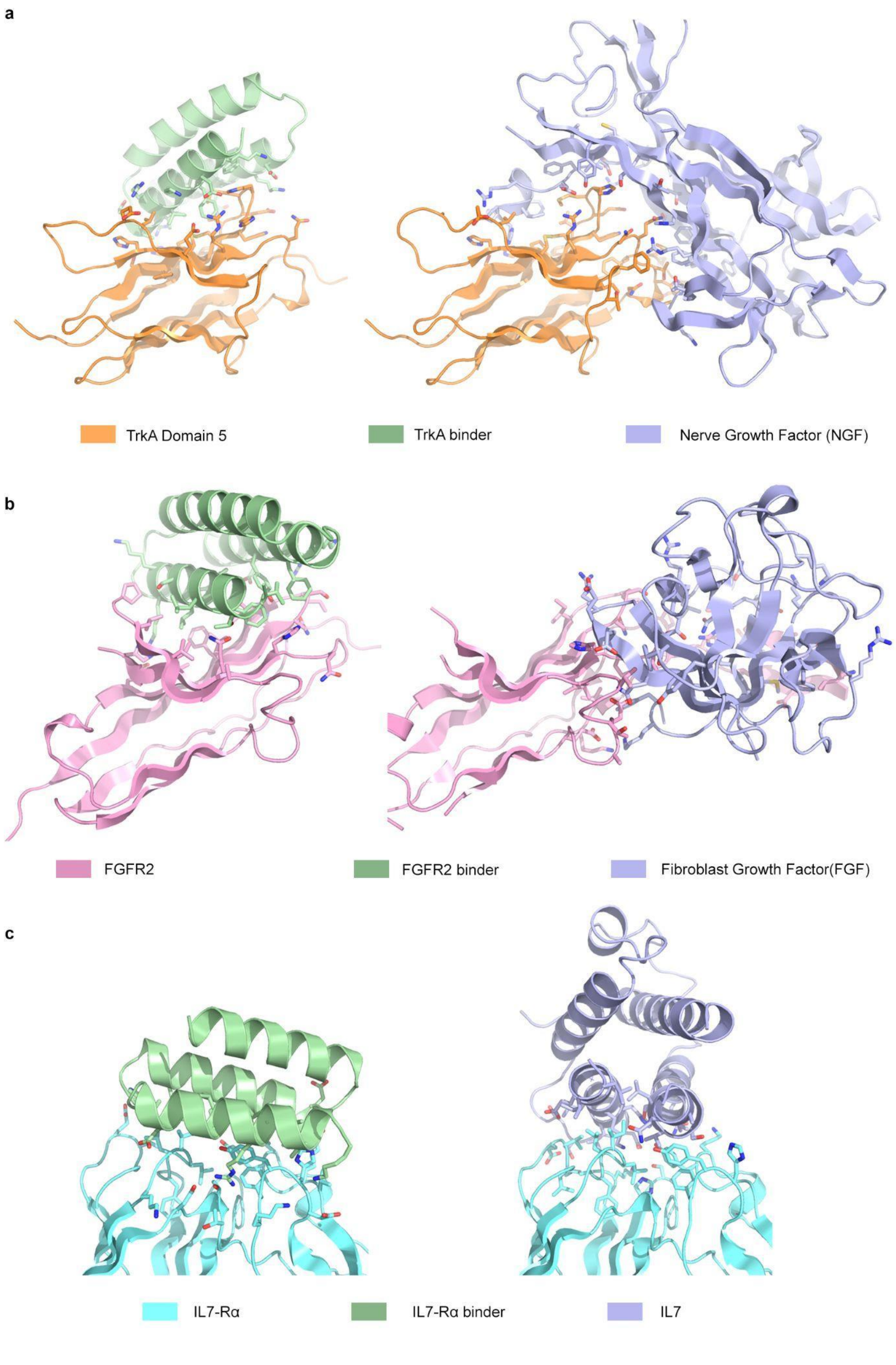

Figure 3. Structure comparison of the binding modes of the miniprotein binders and native ligands with the target proteins. Left, the crystal structure of the de novo

miniprotein binders in complex with TrkA(**a**), FGFR2(**b**) and IL-7Rα(**c**). Right, the crystal structure of the native ligands in complex with the corresponding targets. PDB codes for the native complexes are 1WWW for TrkA, 1EV2 for FGFR2 and 3DI3 for IL-7Rα.

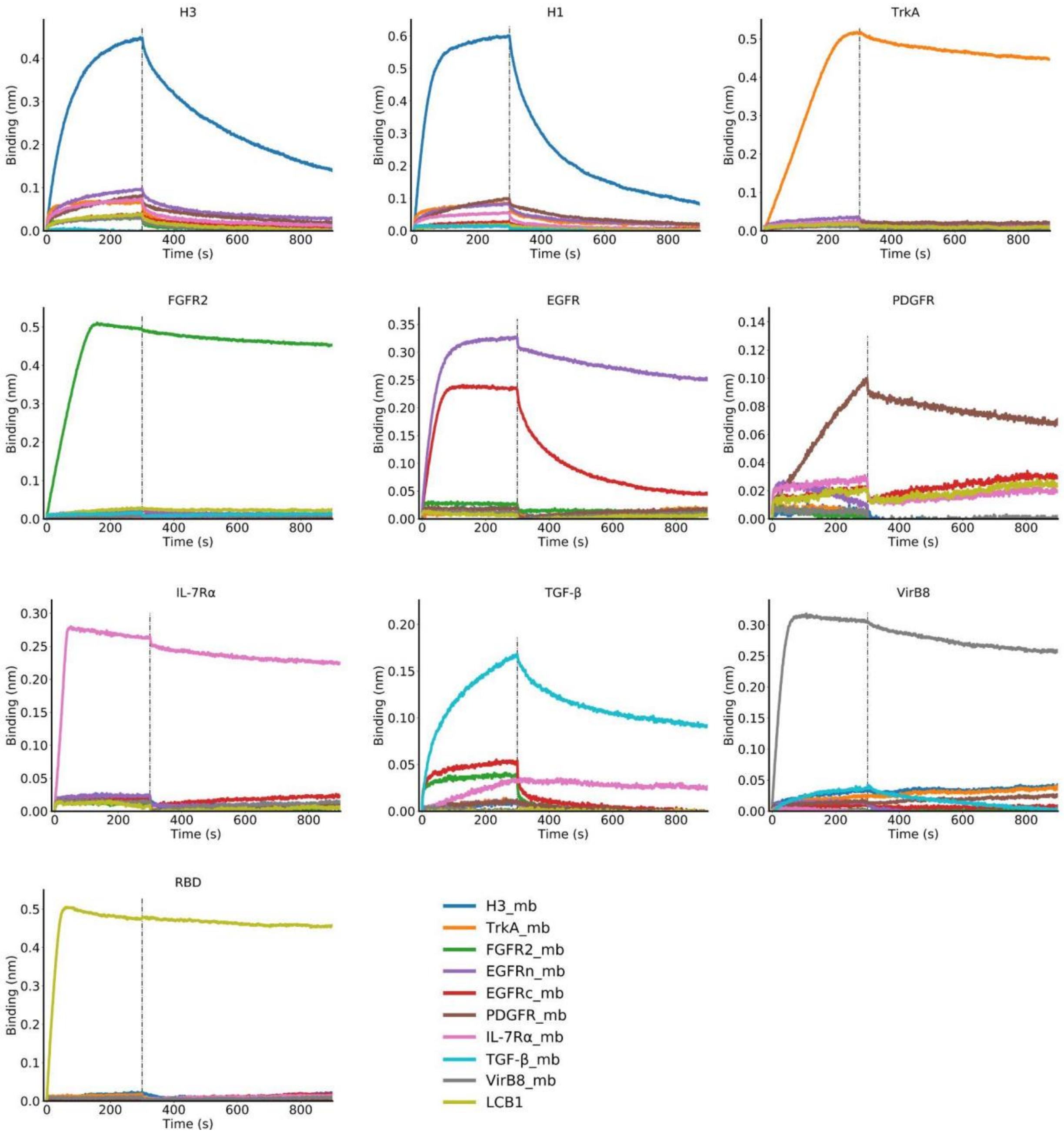

Figure 4. Designed binders have high target specificity. To assess the cross reactivity of each miniprotein binder with each target protein, The biotinylated target proteins were loaded onto biolayer interferometry SA sensors, allowed to equilibrate, and baseline signal set to zero. The BLI tips were then placed into 100 nM binder solution for 300 seconds, washed with buffer, and dissociation was monitored for an additional 600 seconds. The raw traces were used to create the cross reactivity matrix. Each heat map value corresponds to the maximum response value of a single trace per pair, and the value was normalized dividing by the maximum response for the cognate pair. In such a way, the cognate pair always has a value 1, while the values of the non-cognate pairs

indicate the non-specificity of that binder. Heatmap shows the maximum response signal for each binder-target pair normalized by the maximum response signal of the cognate designed binder-target pair.

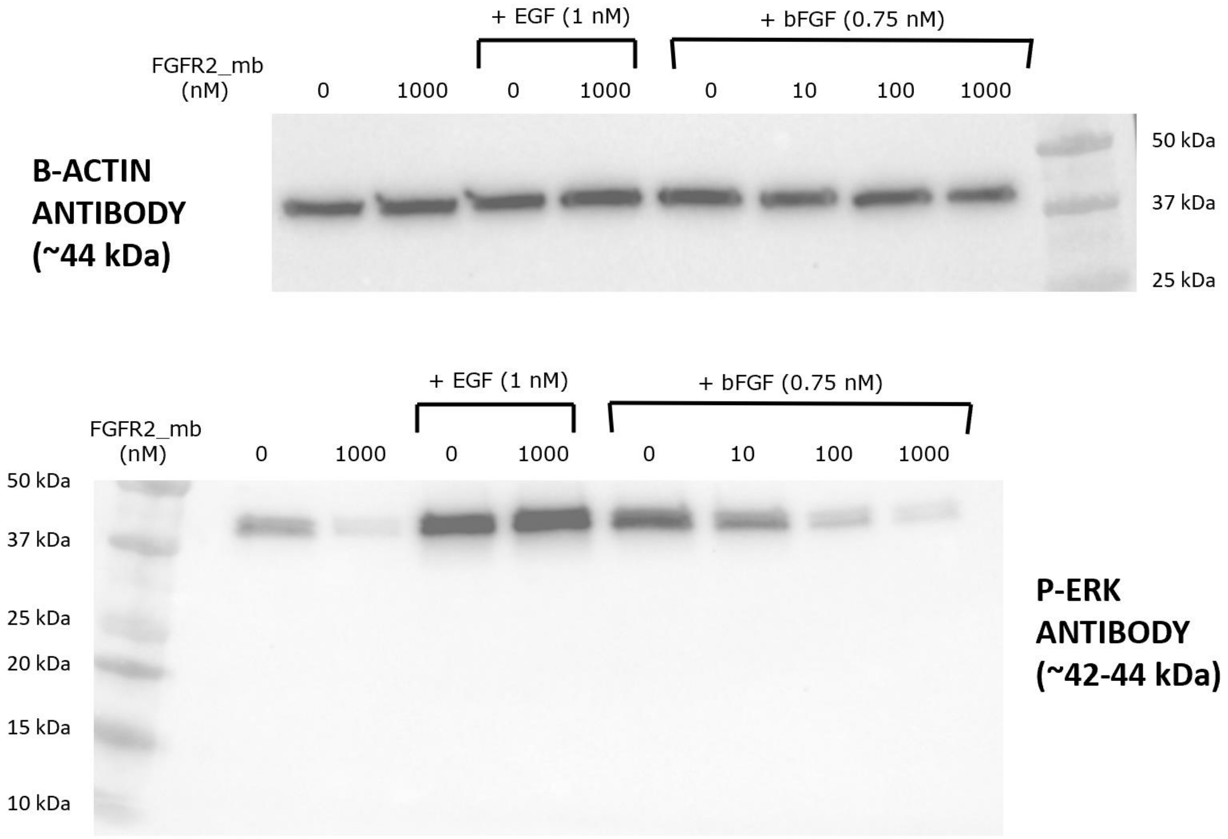

Figure 5a. Display of original blots for B-actin and pERK Western blot analysis (Extended Data Figure 9a). Displayed as composite image (chemiluminescent blot with molecular markers overlaid)

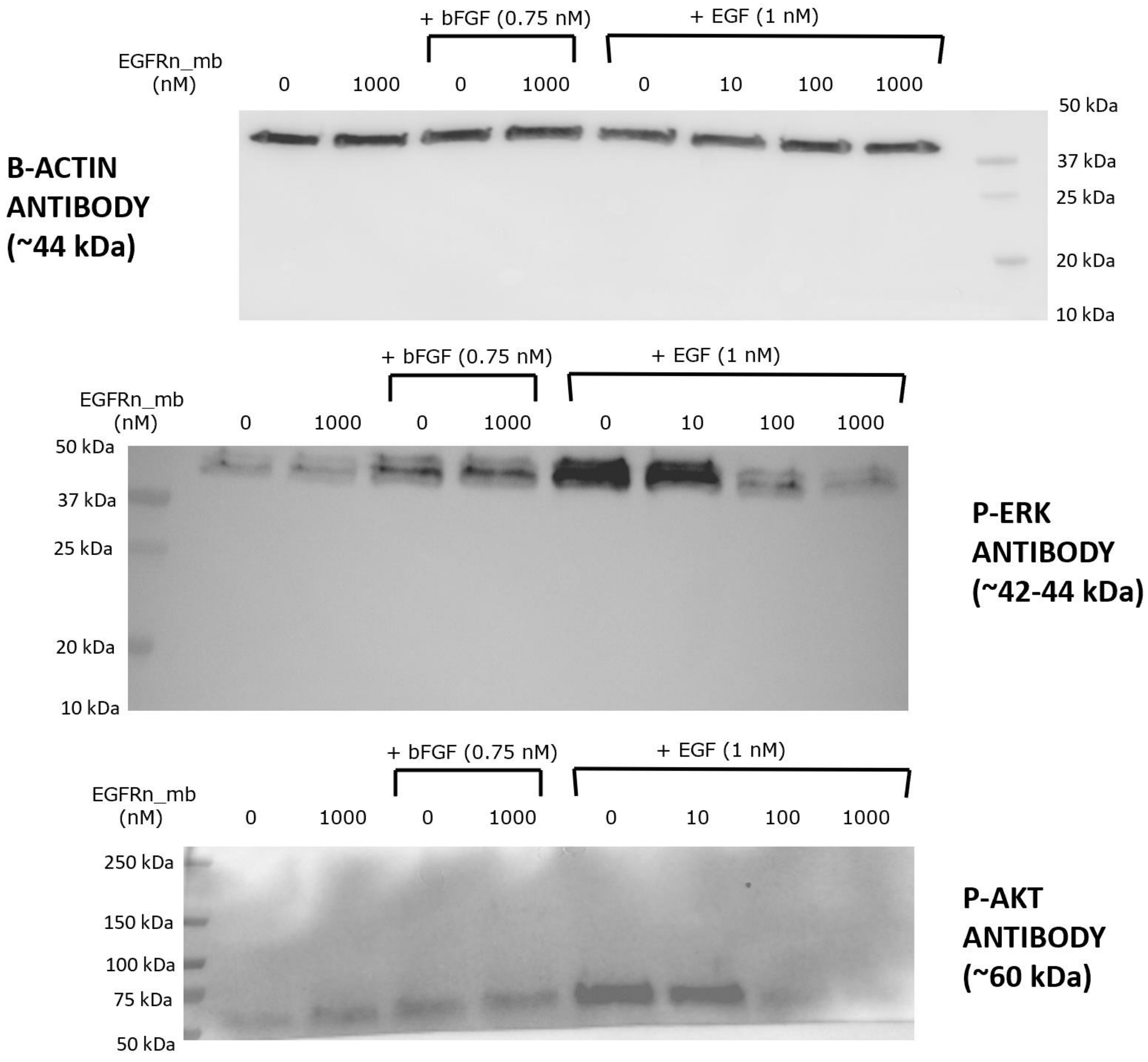

Figure 5b. Display of original blots for B-actin, pERK and pAKT Western blot analysis (Extended Data Figure 12b). Displayed as composite image (chemiluminescent blot with molecular markers overlaid)

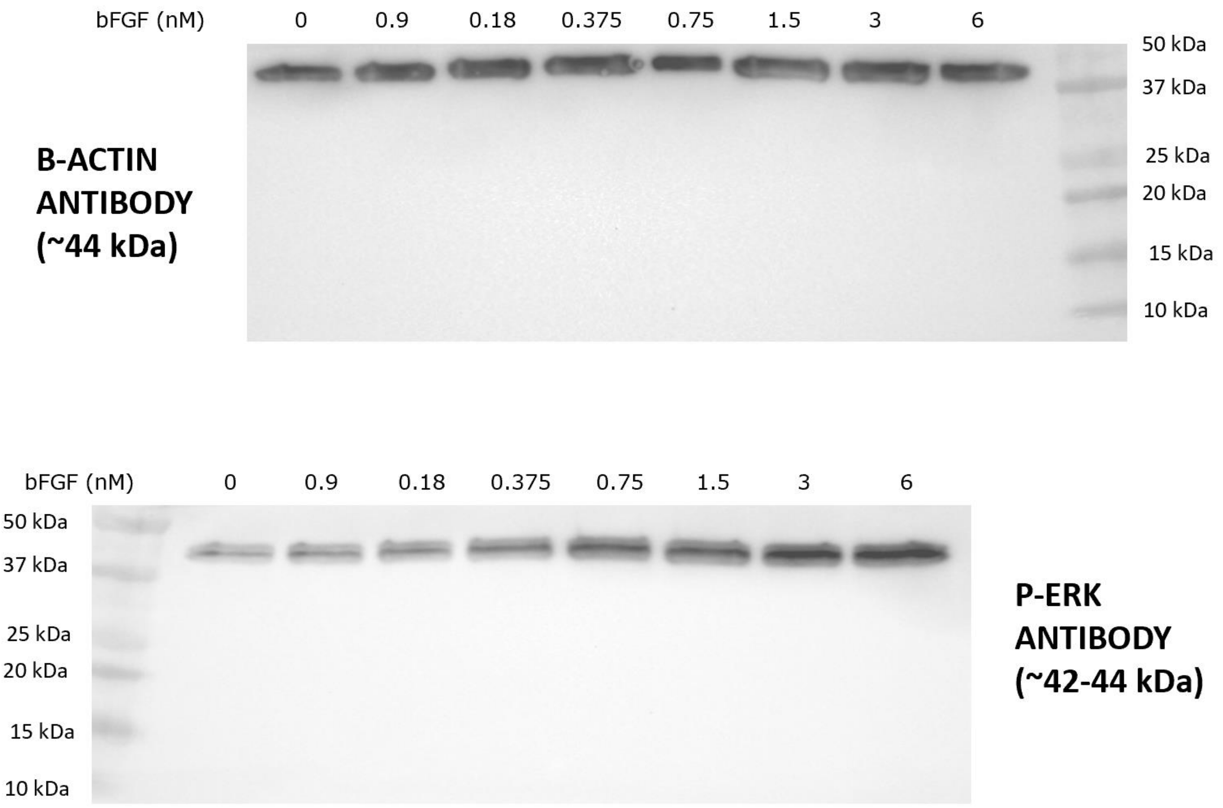

Figure 5c. Display of original blots for B-actin and pERK Western blot analysis (Extended Data Figure 9c). Displayed as composite image (chemiluminescent blot with molecular markers overlaid)

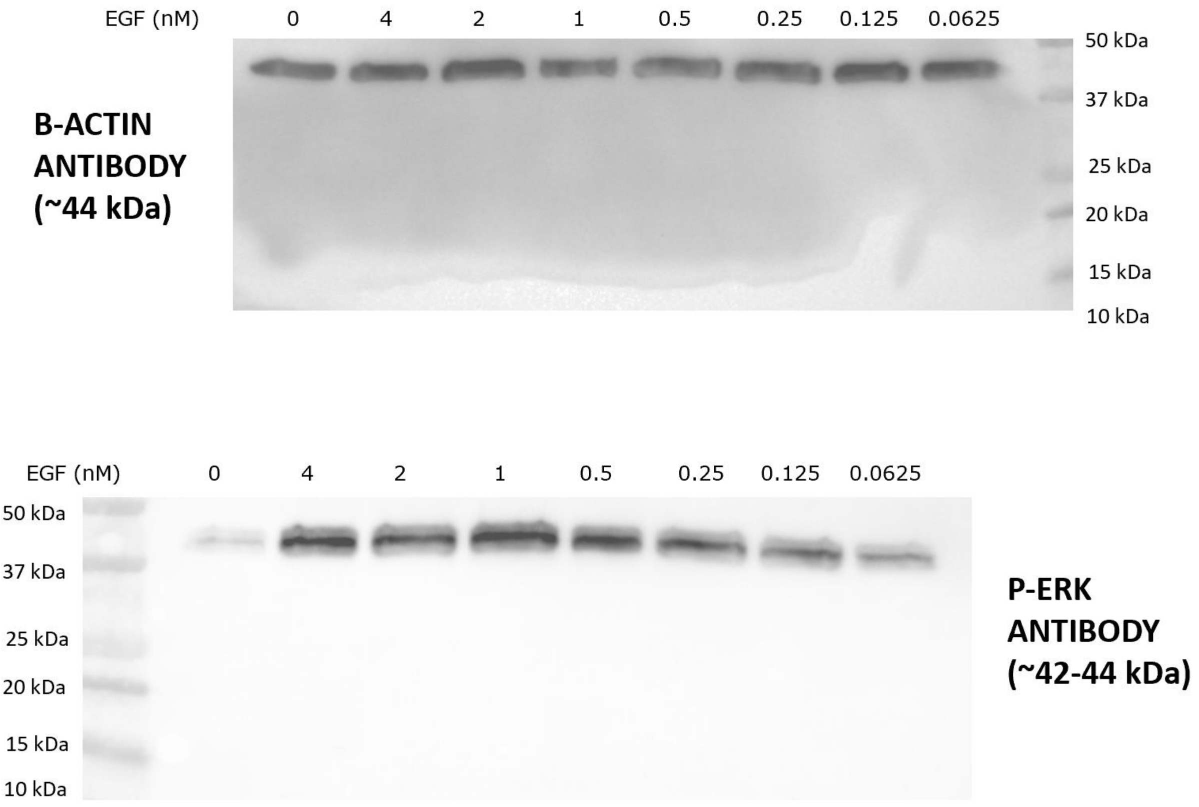

Figure 5d. Display of original blots for B-actin and pERK Western blot analysis (Extended Data Figure 9d). Displayed as composite image (chemiluminescent blot with molecular markers overlaid)

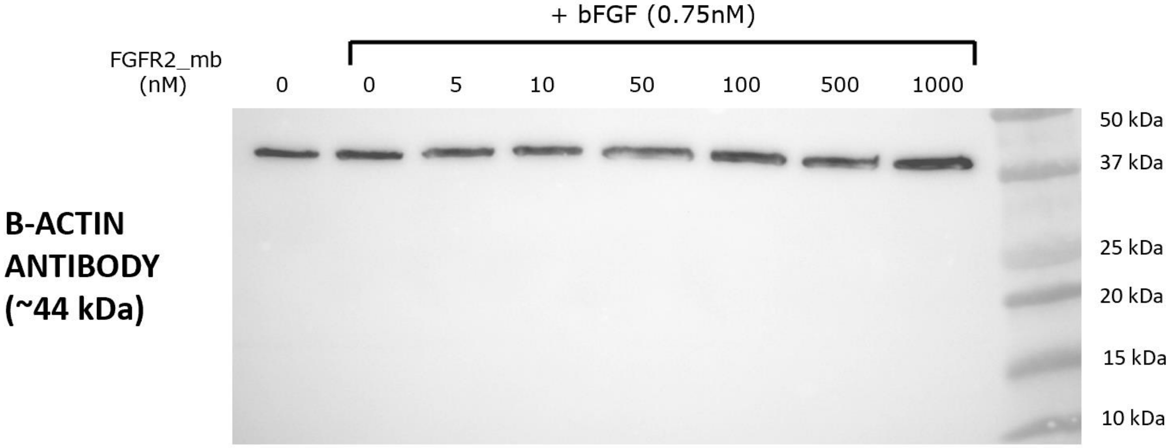

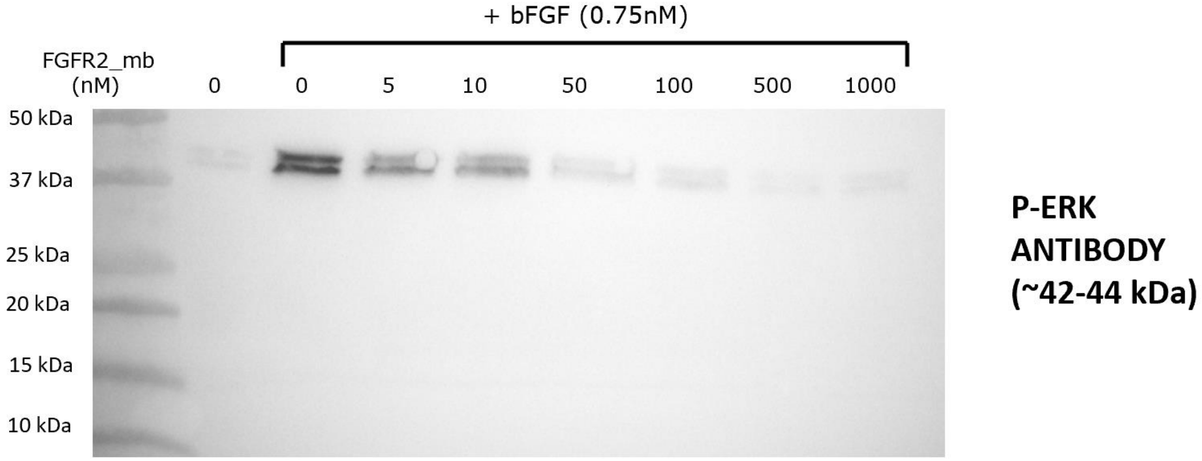

Figure 5e. Display of original blots for B-actin and pERK Western blot analysis (Extended Data Figure 9e). Displayed as composite image (chemiluminescent blot with molecular markers overlaid)

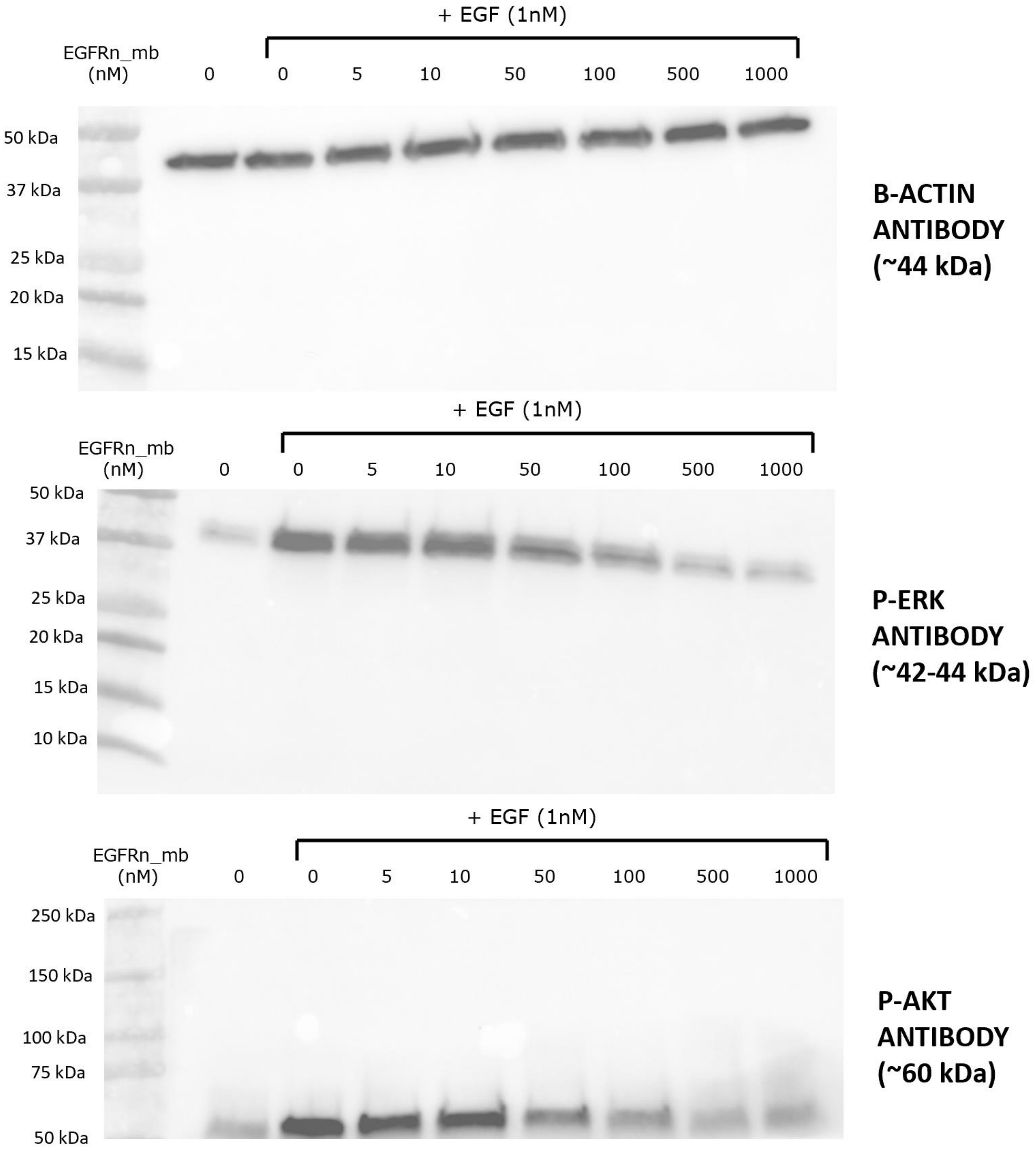

Figure 5f. Display of original blots for B-actin, pERK and pAKT Western blot analysis (Extended Data Figure 9f). Displayed as composite image (chemiluminescent blot with molecular markers overlaid)

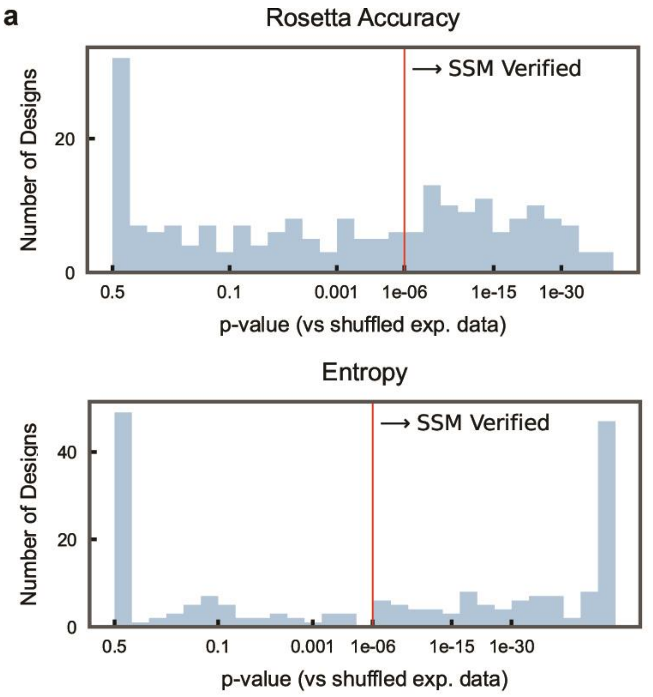

b SSM Fingerprint Scores

Binder	P-Rosetta	P-Entropy
H3_mb	1e-16	5e-62
TrkA_mb	6e-08	6e-14
FGFR2_mb	2e-08	5e-05
EGFRn_mb	4e-17	1e-31
EGFRc_mb	4e-12	3e-08
PDGFR_mb	5e-10	3e-04
InsulinR_mb	9e-07	7e-13
IGF1R_mb	7e-01*	6e-22
Tie2_mb	3e-03	4e-11
IL-7Rα_mb	4e-16	2e-10
CD3δ_mb	1e-01*	5e-02*
TGF-β_mb	2e-04	6e-05
VirB8_mb	1e-24	2e-36
LCB1	2e-03	5e-03
LCB3	5e-04	3e-21

*Values below the threshold p-value of 0.005

Figure 6. SSM Fingerprint Validation. a, SSM Fingerprint validation distributions. As described in the methods, the p-value that the experimental data could be randomly shuffled given the design model is assessed. Lower p-values give greater confidence that the design model matches the experimental data, while p-values near 1 indicate that the design model has little predictive power in predicting the data. A design must score better (lower) than 0.005 on both metrics to be considered verified. This value was chosen because LCB1 received this score in its Entropy category, and LCB1 was confirmed via Electron Microscopy (LCB1's P-entropy was the worst score of any structurally verified binder in this manuscript). Extended Data Table 4 gives the calculated values for the 12 characterized binders with the rest available in the Supplemental Information. **b**, Table of the SSM fingerprint scores**.** The SSM fingerprint scores for the 12 characterized binders are shown as well as the two Cryo-EM verified SARS-CoV-2 binders. Using LCB1's P-Entropy column as the reference for verification, all but CD3δ_mb and IGF1R_mb pass this validation metric in both columns. For the values that are below the threshold p-value of 0.005, possible explanations for the failures are that the IGF1R design model was lost (user error) and had to be reconstructed via prediction. The CD3δ binder is weak and the target protein is sticky. K/O mutations may still be able to bind via alternate binding configurations.

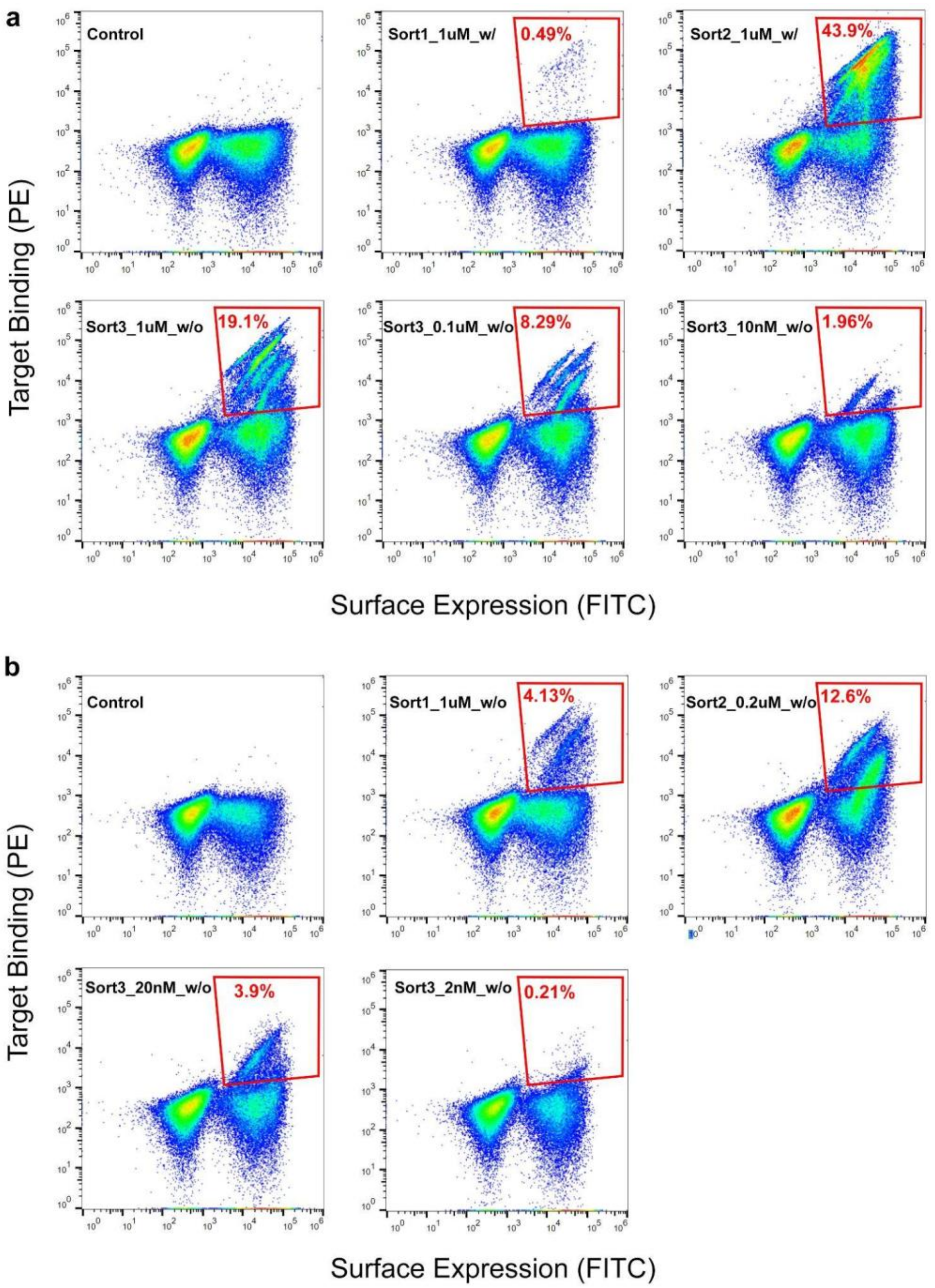
a
Control
Sort1_1uM_w/
0.49%
Sort2_1uM_w/
43.9%
Sort3_1uM_w/o
19.1%
Sort3_0.1uM_w/o
8.29%
Sort3_10nM_w/o
1.96%
Target Binding (PE)
Surface Expression (FITC)
b
Control
Sort1_1uM_w/o
4.13%
Sort2_0.2uM_w/o
12.6%
Sort3_20nM_w/o
3.9%
Sort3_2nM_w/o
0.21%
Target Binding (PE)
Surface Expression (FITC)

c

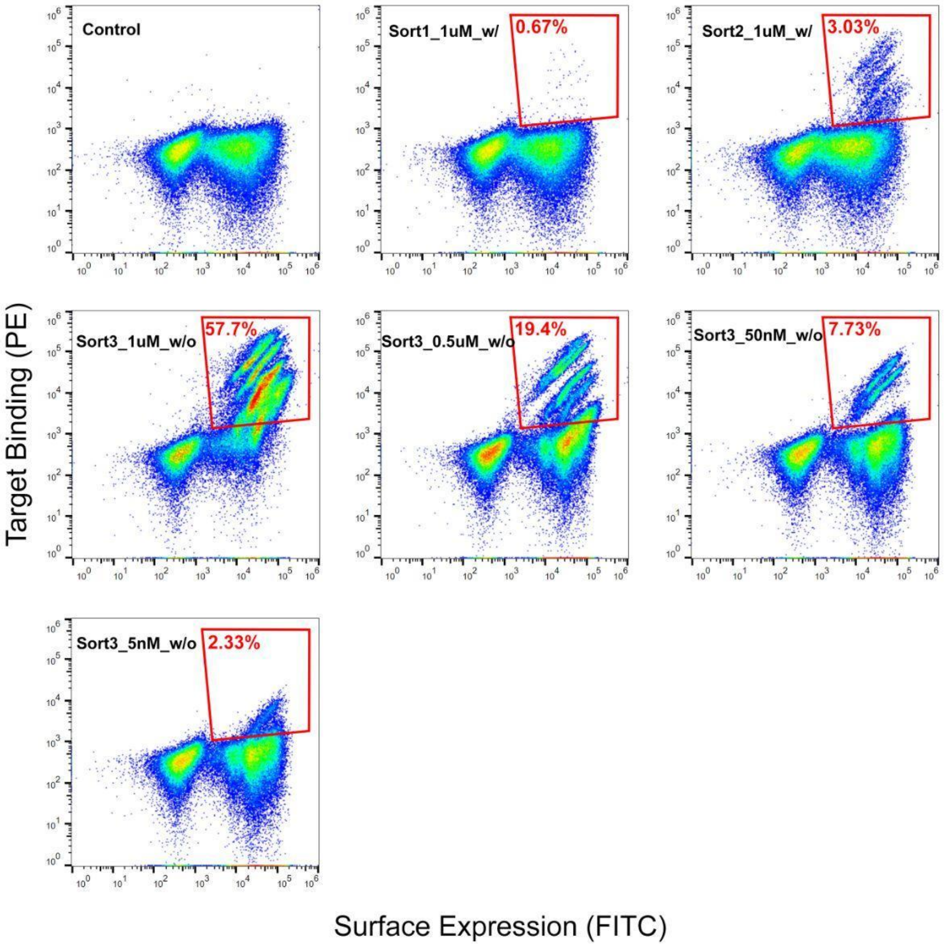
Control
Sort1_1uM_w/
0.67%
Sort2_1uM_w/
3.03%
Sort3_1uM_w/o
57.7%
Sort3_0.5uM_w/o
19.4%
Sort3_50nM_w/o
7.73%
Sort3_5nM_w/o
2.33%
Target Binding (PE)
Surface Expression (FITC)

d

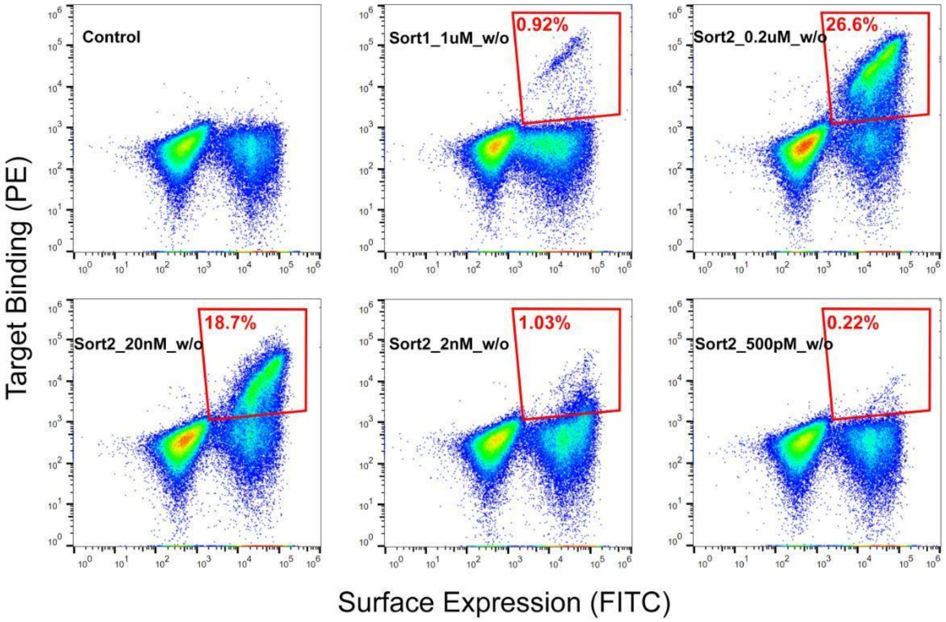
Control
Sort1_1uM_w/o
0.92%
Sort2_0.2uM_w/o
26.6%
Sort2_20nM_w/o
18.7%
Sort2_2nM_w/o
1.03%
Sort2_500pM_w/o
0.22%
Target Binding (PE)
Surface Expression (FITC)

Figure 7. Representative flow cytometry graphs of the large scale experimental testing results. Yeast surface display screening of the original design library are shown in (**a**) for IL-7Rα and (**b**) for TrkA, whereas the results for the site saturation library screening are shown in (**c**) for IL-7Rα and (**d**) for TrkA. The cells labeled without the target protein were used as the negative control and gates were set based on the fluorescent signal of the control. The gates that were used to collect the cells for the next round screening and/or next-gen sequencing are shown as red squares. w/ represents cell labeling using the with avidity condition, and w/o indicates cell labeling using the without avidity condition (see methods for more details).

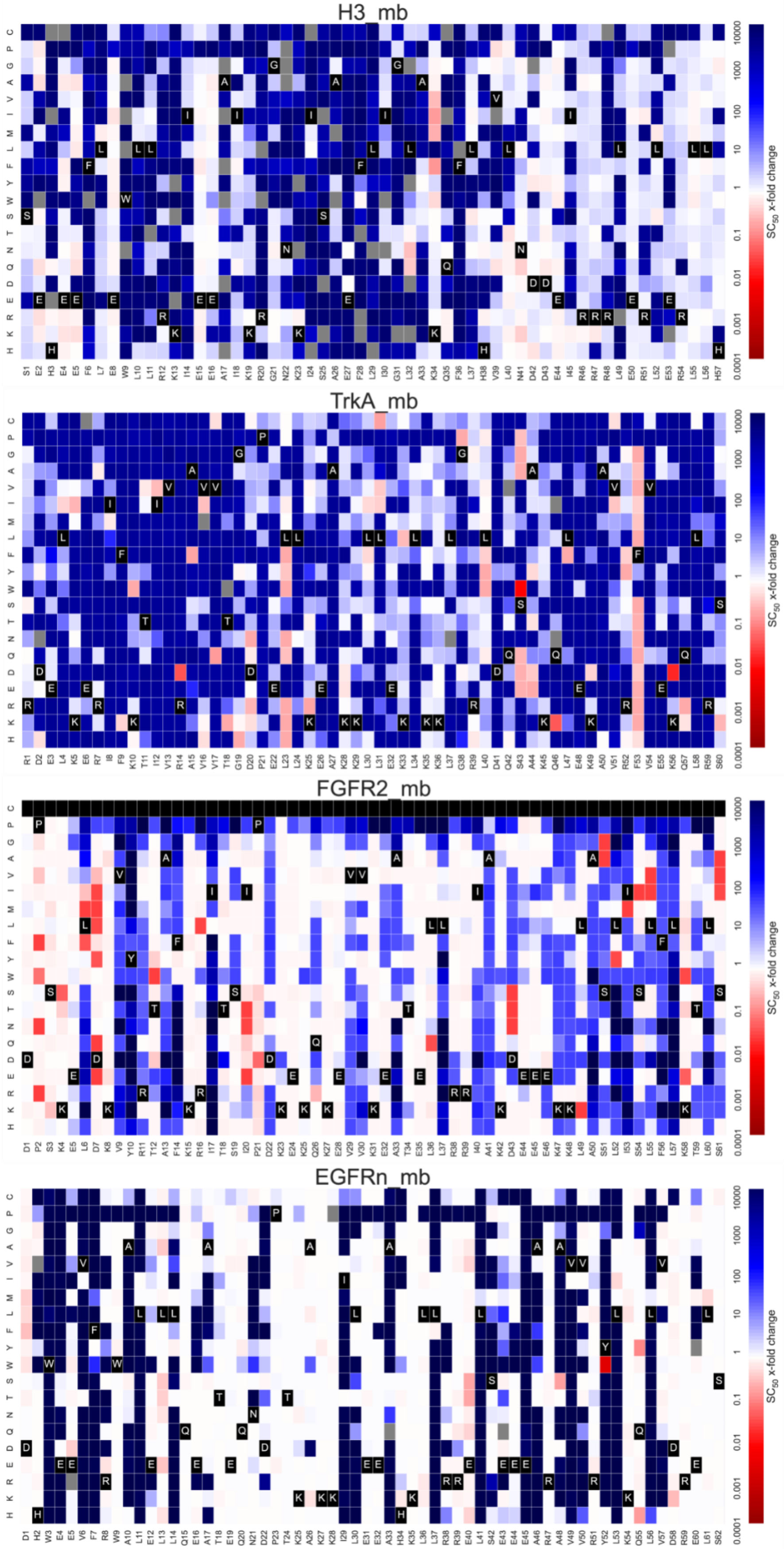

H3_mb
TrkA_mb
FGFR2_mb
EGFRn_mb
SC50 x-fold change

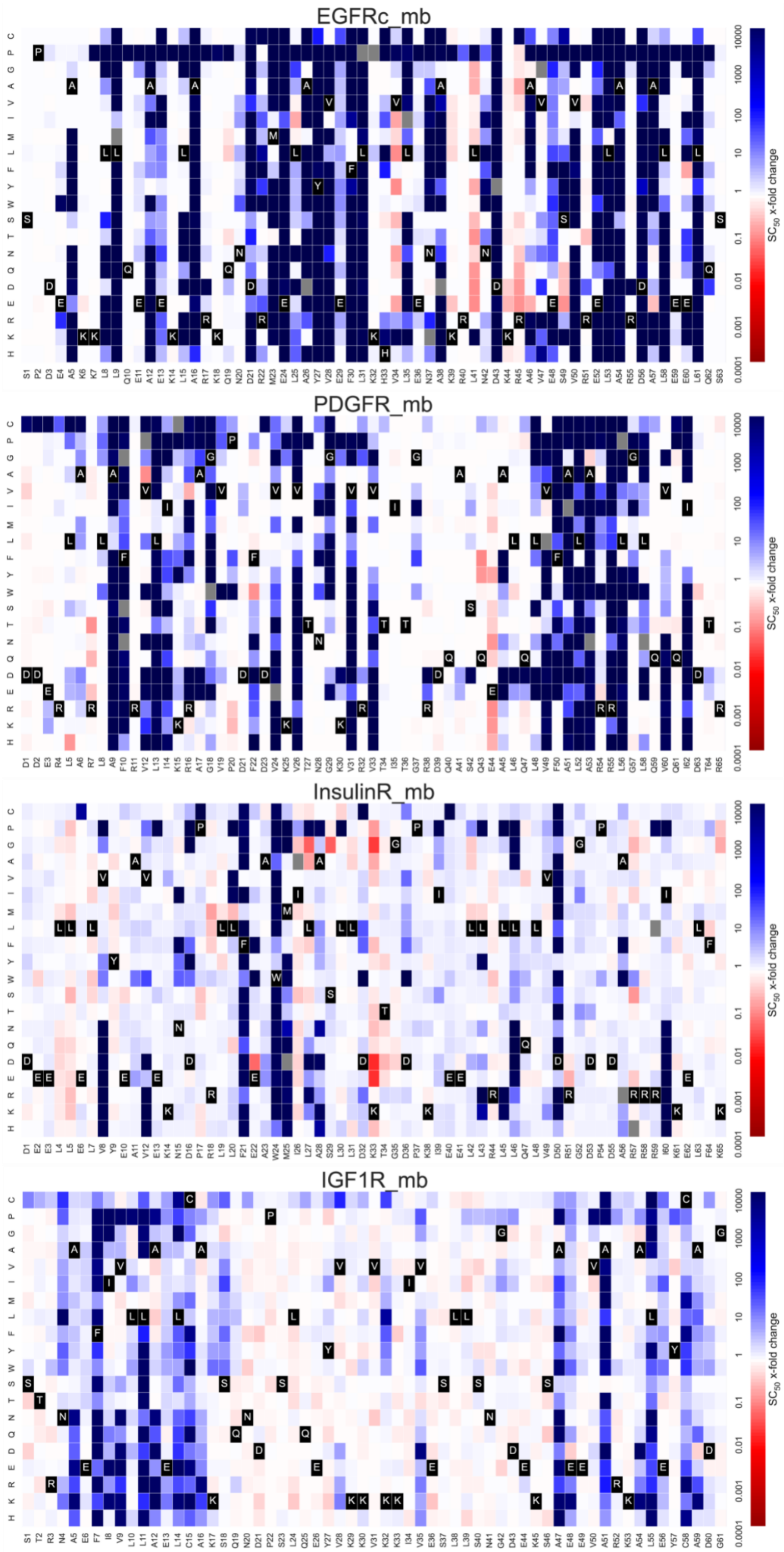
EGFRc_mb
PDGFR_mb
InsulinR_mb
IGF1R_mb
SC50 x-fold change

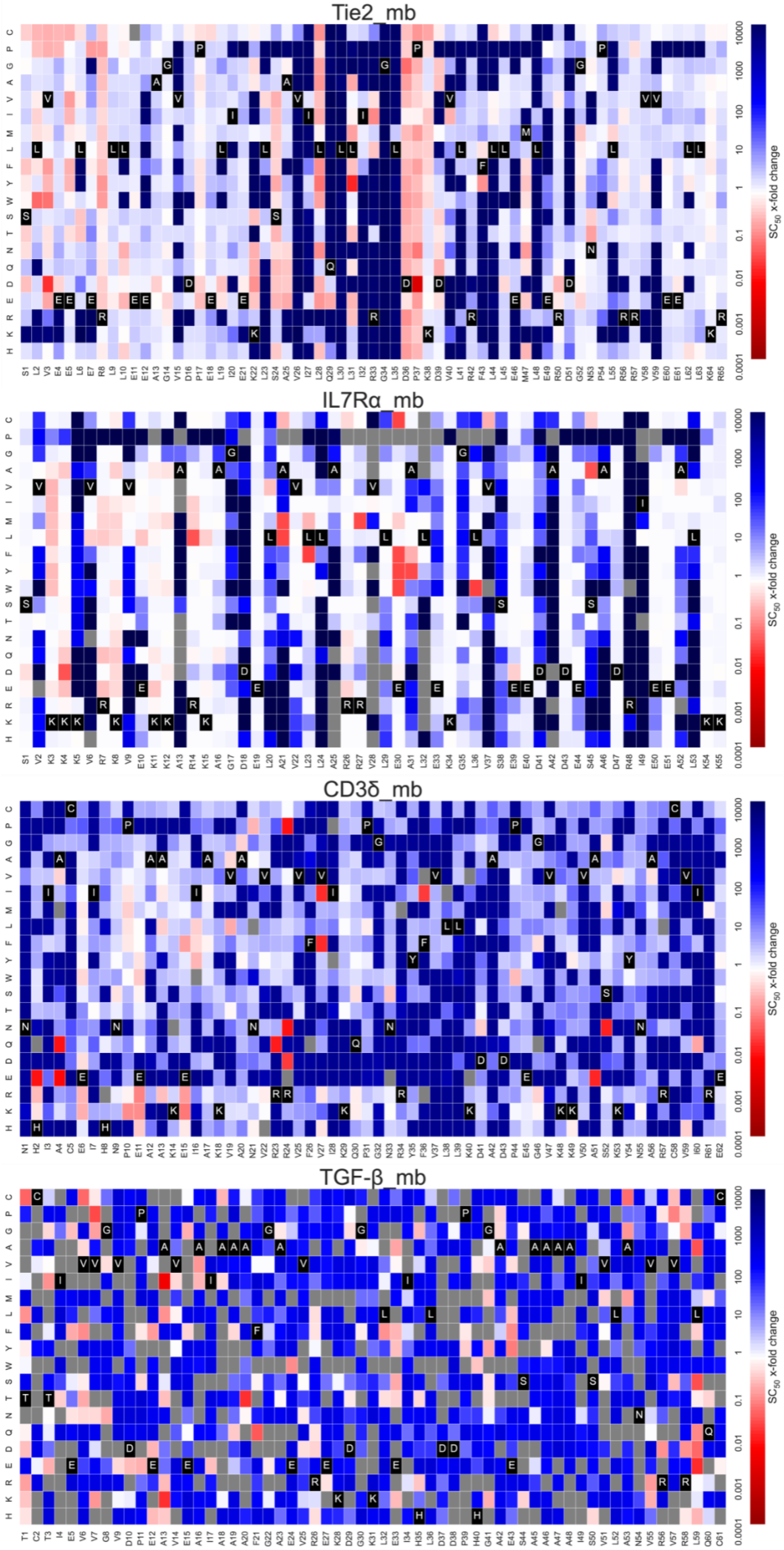
Tie2_mb
IL7Rα_mb
CD3δ_mb
TGF-β_mb
SC50 x-fold change
10000
1000
100
10
1
0.1
0.01
0.001
0.0001
H K R E D Q N T S W Y F L M I V A G P C

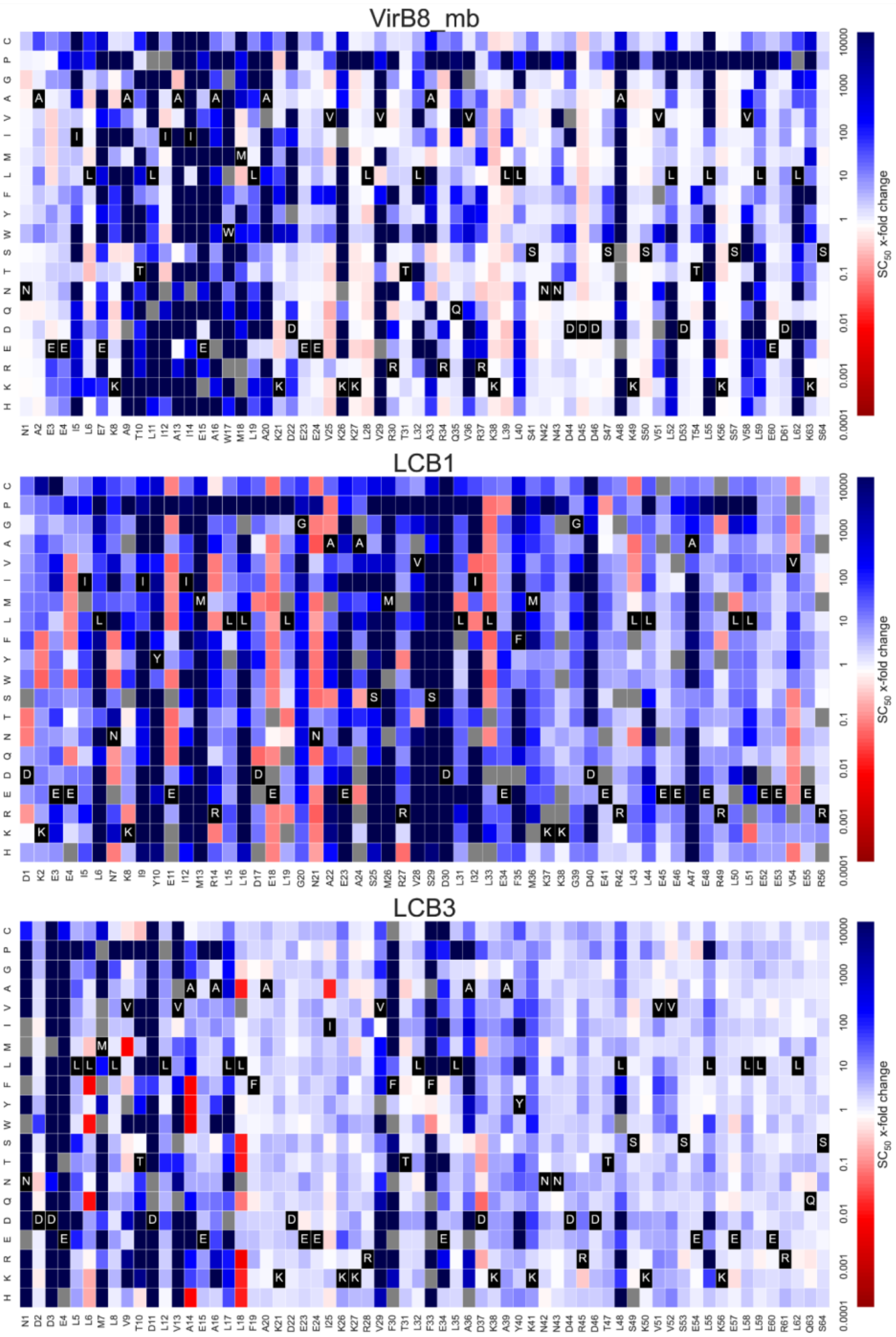

Figure 8. SSM mutational effects. The experimentally observed effect on binding for each mutation is plotted in heatmap style. Red squares improved binding, blue squares made binding worse, and white remained the same. Gray squares had inconsistent data and an SC_{50} could not be reliably determined. Examining this data can be the first step towards qualitative validation by looking for vertical blue bars (positions resistant to

mutation) which hopefully align with the monomer core and interface core positions. While historically this type of data is plotted with enrichments, by using the SC_{50} fitting procedure, data from all sorting pools may be combined giving a higher dynamic range. The SSM graphs for the remaining tested binders are in the Supplemental Information.

Table 1. Topologies, initial amino acid sequences, final optimized amino acid sequences and physicochemical properties of the de novo miniprotein binders for all 12 targets.

Target	Binder	Topology	Original sequence	SC_{50} original sequence (Yeast kD)	Optimized sequence	Num of mutations
H3	H3_mb	HHH	SEHEEFLEWLLRKIEEAIKRGNKISAEFLIGLAKQFLHVLNDDEIRRRLERLERLLH	80 nM*	SQHEKFLEWMLRKIEEAIKRGNKISAEFLINLAKNFIHVLGDDEIRRRLERLERQLH	8
TrkA	TrkA_mb	HHH	RDELKERIFKTIVRAVVTGDPELLKEAKKLLEKLKKLGRLDQSAKQLEKAVRFVEKQLRS	3μM	RDEIKERIFKAVVRAIVTGNPEQLKEAKKLLEKLKKLGRLDQDAKKFEKAIRQVEKRLRS	12
FGFR2	FGFR2_mb	HHH	DPSKELDKVYRTAFKRITSIPDKEKQKEVVKEATELLRRIAKDEEEKKLASLISLFLKTLS	600 pM	DRRKEMDKVYRTAFKRITSTPDKEKRKEVVKEATEQLRRIAKDEEEKKKAAYMILFLKTLG	12
EGFR	EGFRn_mb	HHH	DHWEEVFRWALELLQEATEQNDPTKAKKILEEAHKLLRRELSEEEARAVVRYLKQLVDRELS	300 nM	DHWEEVFRWALEHLQEATQQNDPQKAKKILEEAHKWLRRELSEEEARAVVRWLKQLVDRELS	5
EGFR	EGFRc_mb	HHH	SPDEAKKLLQEAEKLARKQNDRMELAYVEFLKHVLENAKRLNDKRAVESVRELARDALEELQS	200 nM	SLDEAKKLLQEAEKLARKLNDRMELAYVEFLKHILETAKKQNDKRTIESVRDMARDALEELQS	10
PDGFR	PDGFR_mb	HEEHE	DDERLARLAFRVLIKRAGVPDFDVKVTNGKVRVTITGRDQASQEALQLVFALARRLGLQVQIDTR	600 nM	DDERLATLAFRALIKRAGVKNLDVKVTNGKVRVTITGRDQASFKALQLVFALARRLGLQVQIDTR	7
InsulinR	InsulinR_mb	HHHH	DEELLELVYEAVEKNDPRLLFEAWMILASLLDKTGDPKIEELLRLLQLVDRGDPDARRRIKELFK	800 nM	DEELMELVYEAVEKNDPELLFEAWMELASLLDETGDPKIEEALGLLQQVDGGNPDAGRRIKELFK	10
IGF1R	IGF1R_mb	HHH	STRNAEFIVLLAELCAKSQNDPSLQEYVKKVKKIVESLLSNGDEKSAEEVARKALEYCADG	10μM*	STRNAEFIMLLLELCVKSKNDPQVQEYVKKVKKQVERLVGNGDEKKAEEVARKALEYCADG	11
Tie2	Tie2_mb	HHHH	SLVEELERLLEEAGVDPELIEKLSAVILQLLIRGLDPKDVLRFLLEMLERDGNPLRRVVEELLKR	600 nM	SIDEELERLLEEAGVDPELIDDLYAVIYQLYIRGLSDKDVLRFLLENLERDGTPLRRVVEELLKR	11
IL-7Rα	IL-7Rα_mb	HHH	SVKKKVRKVEKKARKAGDELAVLLARRVLEALEKGLVSEEDADESADRIEEALKK	3 nM	SVIEKLRKLEKQARKQGDEVLVMLARMVLEYLEKGWVSEEDADESADRIEEVLKK	13
CD3δ	CD3δ_mb	EHEEHE	NHIACEIHNPEAAKEIAKVANVRRVFVIKQPGNRYFVLLKDADPEGVKKVASKYNARCVIRE	31 μM*	NHIACEIHNPEAAKEIAKVANVRRVYFIKQPGNRYFVLLKNADPEGVKKVRSKYNVRCVIRE	5
TGF-β	TGF-β_mb	EHEEHE	TCTIEVVGVDPEAVEAIAAAFGAEVREKDGKLEIHLDDPHGAESAAAAISVLANVRVRLQC	30 nM	HCTIEVVGVDPEKVEAIAAAYGAEVCEKDGKFEIHLDDPHSAESAAVAISVLTNRPVRLQC	10
VirB8	VirB8_mb	HHH	NAEEILEKATLIAIEAWMLAKDEEVKKLVRTLARQVRKLLSNNDDDSAKSVLDTLKSVLEDLKS	100 nM	NAEEITEKATLVGIEAWLLAKDEEQKKKVRTLNRQVKKLLQQNDLDQAKRVLDQLKSVLEDLKS	14

SC_{50} values from initial library unless * (* from SSM library).

Information for downloading the raw design models and design scripts

```
# The supplement for Cao 2021  De novo design of protein binding proteins
from target structure alone
#  has been divided across several files in order to aid with downloading.
#
# Some of these files are very large...

# The main supplement. This is what you want if you aren't here to analyze
our data.
#   Contains these files:
#      cao_2021_protocol/
#      design_models_final_combo_optimized/
#      design_models_sequence/
#      design_models_ssm_natives/
#      ngs_analysis_scripts/

# 61 MB
files.ipd.uw.edu/pub/robust_de_novo_design_minibinders_2021/supplemental_file
s/scripts_and_main_pdbs.tar.gz


# In this file, you will find all of our experimental data and data derived
from that data.
#   Contains these files:
#      ngs_analysis/
#      sorting_ngs_data/

# 234 MB
files.ipd.uw.edu/pub/robust_de_novo_design_minibinders_2021/supplemental_file
s/experimental_data_and_analysis.tar.gz


# In this file, you will find all of the scaffolds we used in this work.
#   Contains these files:
#      scaffolds/

# 1.3 GB
files.ipd.uw.edu/pub/robust_de_novo_design_minibinders_2021/supplemental_file
s/scaffolds.tar.gz


# In this file, you will find all of the computational analysis we did for
Fig1 and SFigs. There is no experimental data here!
#   Contains these files:
#       computational_protocol_analysis/

# 69 MB
files.ipd.uw.edu/pub/robust_de_novo_design_minibinders_2021/supplemental_file
s/computational_protocol_analysis.tar.gz


##############################################################################
##################################################
#
```

```
#                                                 LAND OF THE BIG FILES
#
#
############################################################################
################################################

# These files are big! Think carefully here about whether you actually need
every single design we ordered, or if
#   the design_models_ssm_natives/ above will satisfy your needs.

# If you are on an academic network, you may substitute files.ipd with
research-files.ipd . This uses the academic internet
#  to give you faster download speeds.


# All ordered proteins in .pdb.gz format: (There are 1M+ files here)
#   Contains these files:
#      design_models_pdb/

# 64 GB
files.ipd.uw.edu/pub/robust_de_novo_design_minibinders_2021/supplemental_file
s/design_models_pdb.tar.gz



# All ordered proteins in Rosetta binary silent format. If you're using
Rosetta, it's worth your time to figure out how to load these.
#  These will load 10x faster than the pdb files. (And the .tar.gz only
contains 30 files). See also github.com/bcov77/silent_tools
#   Contains these files:
#      design_models_silent/

# 46 GB
files.ipd.uw.edu/pub/robust_de_novo_design_minibinders_2021/supplemental_file
s/design_models_silent.tar.gz
```
