# Supplementary material for: Design of protein-binding proteins from the target structure alone
Source: Nature. 2022 Mar 24;605(7910):551–60. doi: 10.1038/s41586-022-04654-9 (PMC9117152; doi:10.1038/s41586-022-04654-9)
Supplement: Supplementary file 2 — Reporting Summary [file 41586_2022_4654_MOESM2_ESM.pdf]

nature portfolio

Corresponding author(s): David Baker

Last updated by author(s): Dec 7, 2021

Reporting Summary

Nature Portfolio wishes to improve the reproducibility of the work that we publish. This form provides structure for consistency and transparency in reporting. For further information on Nature Portfolio policies, see our Editorial Policies and the Editorial Policy Checklist.

Statistics

For all statistical analyses, confirm that the following items are present in the figure legend, table legend, main text, or Methods section.

n/a	Confirmed	
☐	☒	The exact sample size (*n*) for each experimental group/condition, given as a discrete number and unit of measurement
☐	☒	A statement on whether measurements were taken from distinct samples or whether the same sample was measured repeatedly
☒	☐	The statistical test(s) used AND whether they are one- or two-sided<br>*Only common tests should be described solely by name; describe more complex techniques in the Methods section.*
☐	☒	A description of all covariates tested
☐	☒	A description of any assumptions or corrections, such as tests of normality and adjustment for multiple comparisons
☐	☒	A full description of the statistical parameters including central tendency (e.g. means) or other basic estimates (e.g. regression coefficient) AND variation (e.g. standard deviation) or associated estimates of uncertainty (e.g. confidence intervals)
☐	☒	For null hypothesis testing, the test statistic (e.g. *F*, *t*, *r*) with confidence intervals, effect sizes, degrees of freedom and *P* value noted<br>*Give P values as exact values whenever suitable.*
☒	☐	For Bayesian analysis, information on the choice of priors and Markov chain Monte Carlo settings
☒	☐	For hierarchical and complex designs, identification of the appropriate level for tests and full reporting of outcomes
☒	☐	Estimates of effect sizes (e.g. Cohen's *d*, Pearson's *r*), indicating how they were calculated

Our web collection on statistics for biologists contains articles on many of the points above.

Software and code

Policy information about availability of computer code

Data collection	Rosetta Macromolecular Modeling Suit; RifDock (https://github.com/rifdock/rifdock);
Data analysis	Python 3.8; ForteBio Data Analysis Software Version 9.0.0.14; FlowJo v10.6.2; Coot-0.8.9.0; Phenix-1.19.2; DNAWorks2.0;

For manuscripts utilizing custom algorithms or software that are central to the research but not yet described in published literature, software must be made available to editors and reviewers. We strongly encourage code deposition in a community repository (e.g. GitHub). See the Nature Portfolio guidelines for submitting code & software for further information.

Data

Policy information about availability of data

All manuscripts must include a data availability statement. This statement should provide the following information, where applicable:

- Accession codes, unique identifiers, or web links for publicly available datasets
- A description of any restrictions on data availability
- For clinical datasets or third party data, please ensure that the statement adheres to our policy

The atomic coordinates and experimental data of H3_mb in complex with H3 HA, TrkA_mb in complex with TrkA, unbound FGFR2_mb, FGFR2_mb in complex with FGFR4, unbound IL-7Rα_mb, IL-7Rα_mb in complex with IL-7Rα and VirB8_mb in complex with VirB8 have been deposited in the RCSB Protein Database with the accession numbers of 7RDH, 7N3T, 7N1K, 7N1J, 7S5B, 7OPB and 7SH3 respectively. Diffraction images for the TrkA minibinder complex have been deposited in the SBGrid Data Bank with ID 838.

Field-specific reporting

Please select the one below that is the best fit for your research. If you are not sure, read the appropriate sections before making your selection.

☒ Life sciences ☐ Behavioural & social sciences ☐ Ecological, evolutionary & environmental sciences

For a reference copy of the document with all sections, see nature.com/documents/nr-reporting-summary-flat.pdf

Life sciences study design

All studies must disclose on these points even when the disclosure is negative.

Sample size	15,000 to 100,000 designs were ordered for each targeting site and this depends on the Angilent Oligo library size. No statistical method was used to determine the total number of designs to be experimentally tested. The numbers are chosen because the size of an Angilent Oligo Pool is 15,000 or 60,000.
Data exclusions	There is no data exclusion in this study.
Replication	Experimental finders were statistically significant and no attempt at reproduction was performed.
Randomization	For the cell signaling assay, the cells were randomly separated into group and then treated with different concentrations of miniprotein binders.
Blinding	For the cell signaling assay, researchers were not blinded to different cell groups.

Reporting for specific materials, systems and methods

We require information from authors about some types of materials, experimental systems and methods used in many studies. Here, indicate whether each material, system or method listed is relevant to your study. If you are not sure if a list item applies to your research, read the appropriate section before selecting a response.

Materials & experimental systems

n/a	Involved in the study	
☐	☒	Antibodies
☐	☒	Eukaryotic cell lines
☒	☐	Palaeontology and archaeology
☒	☐	Animals and other organisms
☒	☐	Human research participants
☒	☐	Clinical data
☒	☐	Dual use research of concern

Methods

n/a	Involved in the study	
☒	☐	ChIP-seq
☐	☒	Flow cytometry
☒	☐	MRI-based neuroimaging

Antibodies

Antibodies used	FI6v3 antibody was kindly provided by Deborah H. Fuller at University of Washington; Alexa Fluor 488 conjugated anti-ERK1/2 pT202/pY204 antibody for BD Bioscience; Alexa Fluor 647 conjugated anti-Akt pS473 antibody from Cell Signaling Technology; Anti-rabbit HRP conjugated secondary antibody from Bio-Rad Laboratories; HRP-conjugated secondary antibody from Bio-Rad Laboratories.
Validation	Corti, D. et al. A neutralizing antibody selected from plasma cells that binds to group 1 and group 2 influenza A hemagglutinins. Science333, 850-856, doi:10.1126/science.1205669(2011). For the commercially available antibodies, the researchers didn't do any additional validation.

Eukaryotic cell lines

Policy information about cell lines

Cell line source(s)	TF-1(ATCC CRL-2003); HEK293T (ATCC), Mark Hall, Department of Biochemistry, University of Birmingham, UK; Human Umbilical Vein Endothelial Cells, LONZA, Cat #2519A. Hi5 cells (ATCC)
Authentication	Authenticated by vendors and we didn't do any additional authentication.
Mycoplasma contamination	TF-1, confirmed negative for mycoplasma; HET293T, negative, confirmed by Plasmo Test; Human Umbilical Vein Endothelial Cells, confirmed negative for mycoplasma. Hi5 cells, confirmed negative for mycoplasma.

Commonly misidentified lines (See ICLAC register) | No commonly misidentified cell lines were used in this study.

Flow Cytometry

Plots

Confirm that:

☒ The axis labels state the marker and fluorochrome used (e.g. CD4-FITC).

☒ The axis scales are clearly visible. Include numbers along axes only for bottom left plot of group (a 'group' is an analysis of identical markers).

☒ All plots are contour plots with outliers or pseudocolor plots.

☒ A numerical value for number of cells or percentage (with statistics) is provided.

Methodology

Sample preparation	Yeast Cell are incubated with the target protein and then labeled with anti-Myc Antibody conjugated with FITC and Strepavidin congated with PE. The cells were washed with PBSF. See Methods for experimental details.
Instrument	Sony SH800
Software	FlowJo10
Cell population abundance	Yes
Gating strategy	Cells labeled without the target protein were used as negative control and all the cells showed binding signal were collected.

☒ Tick this box to confirm that a figure exemplifying the gating strategy is provided in the Supplementary Information.
