# Supplementary material for: Design of protein-binding proteins from the target structure alone
Source: Nature. 2022 Mar 24;605(7910):551–60. doi: 10.1038/s41586-022-04654-9 (PMC9117152; doi:10.1038/s41586-022-04654-9)
Supplement: Supplementary file 3 — Supplementary Code Design scripts and main pdb files. This compressed file is the main supplement and contains the following files: => cao_2021_protocol/ => design_models_final_combo_optimized/ => design_models_sequence/ => design_models_ssm_natives/ => ngs_analysis_scripts/ [file 41586_2022_4654_MOESM3_ESM.gz › supplemental_files/dna_production_scripts/combo_creation/swiftlib_javascript.tar.gz!/swiftlib_javascript/score_library.html]

# SwiftLib

## A web-based tool for rapid optimization of degenerate codons

### Details

Purpose

This program optimizes a degenerate codon library to cover the desired
set of amino acids at several positions while staying within a diversity
limit for the library. It is a fast way to generate small libraries.

The typical case for which we imagine SwiftLib to be useful is this: imagine
that you have constructed 100 redesigned models of a particular
protein where you allowed 15 residues to mutate. For position 82 (one of
the residues you allowed to mutate), alanine appeared 10 times, arginine
35 times, lysine 25 times, leucine 20 times, asparagine 5 times, tryptophan
3 times, and valine twice and similar distributions were seen at the other
14 positions. (You would enter all of these counts in SwiftLib's table below.)
Let's say you're aiming to create a library for yeast display
and want to ensure that you don't exceed a (DNA) diversity of 1e7.
In this case, you would rather have a library that excluded tryptophan at
position 82 than one that excluded arginine. Trypotophan's exclusion represents
*error*: you wanted something, but you couldn't get it. The goal of
this program is to find the assignment of degenerate codons within the
given diversity limit that yields the minimal error over all positions being
randomized.

Additionally, it is possible to ask the algorithm to allow multiple degenerate
codons at one or more positions. You simply indicate the primer boundaries and
how many oligos you are willing to buy. The algorithm will choose which positions
to use multiple degenerate codons at to get the best coverage. The number of oligos
that must be purchased to cover the randomized residues
that lie inside the same primer boundaries is the product of the number of degenerate
codons used at each of those positions. E.g. if there are three residues that are part
of the same stretch using, 2, 3, and 4 degenerate codons, then to cover all combinations,
2\*3\*4 = 24 oligos would have to be purchased. One of the more expensive parts of
considering multiple degenerate codons is enumerating all combinations of degenerate
codons; allowing *i* degenerate codons at a single position requires looking at
*(15^3^)^i^* combinations. For this reason we do not recommend
using more than 4 degenerate codons at any one position.

Algorithm

The program works by dynamic programming. If all of the errors are integers,
then it is possible to ask, for each position, what degenerate codon has
the smallest diversity given that it produces a given error. The smallest
library for positions [1..*i*] given a particular error can be readily
computed using a simple recurrence. The best library is the one with the smallest
error with a diversity below the desired cap. The running time is *O(n^2^m^2^)* for
*n* positions and *m* error gradations. In the case above, n=15, and
m=100 (the maximum error would be 100 given by a codon that doesn't contain any
of the desired amino acids; the larger the maximum error the longer the running time).
Analysis for the multiple-degenerate-codon algorithm is slightly more complicated
and will be published shortly.

Privacy? This program is implemented in JavaScript and therefore runs inside your browser.
It does not send any data anywhere. You do not need to worry about anyone decoding the
library you're creating.

Input

The input for SwiftLib is a table of positions for which you would like
to vary in your library, and a numeric preference for each amino acid at
these positions that you would like to favor, or disfavor. So, given the
above example, the input would be a table with 15 columns (1 for each residue),
and the numeric preference for each amino acid would be the number of occurances
of that amino acid at that position. Aside from using positive integers to favor
an amino acid, one can also use negative integers to disfavor an amino acid
at a given positions. Furthermore one can use the '\*' and '!' wildcards to
specify that the amino acid is required ('\*') or forbidden ('!') at the given position.
Empty fields in the table will be treated as if they contain a '0'
For convenience, SwiftLib allows the creation of this table manually, through a CSV
format, or through a collections of FASTA formatted sequences.

Citation

If you use this program in your work, please cite:  
Leaver-Fay, Jacobs, Yumerefendi, & Kuhlman (2014) *in preparation*

Please direct questions to: leaverfa at email dot unc dot edu.

  

- Manual input
- CSV input
- FASTA input
- MSF input

|  | Drag |
| --- | --- |
| Residue Number: |  |
| Primer Boundary |  |
| Max DCs |  || A (Alanine) |  |
| C (Cysteine) |  |
| D (Apsartic Acid) |  |
| E (Glutamic Acid) |  |
| F (Phenylalanine) |  |
| G (Glycine) |  |
| H (Histidine) |  |
| I (Isoleucine) |  |
| K (Lysine) |  |
| L (Leucine) |  |
| M (Methionine) |  |
| N (Asparagine) |  |
| P (Proline) |  |
| Q (Glutamine) |  |
| R (Arginine) |  |
| S (Serine) |  |
| T (Threonine) |  |
| V (Valine) |  |
| W (Tryptophan) |  |
| Y (Tyrosine) |  |
| STOP |  |
| Solution |  |

Manual input instructions

- Each column in the table below represents one position, and each cell in the column (except for the first) represents one amino acid at that position.- The "Add Position" button will add a new column to the right.- The "Delete Position" button will delete the right-most column. All data in the column will be lost when you hit this button, so be careful.- Columns may be dragged left or right by dragging on the word "Drag" at the top of the column.- You must provide a label for each column in the "Res" row. This is for output purposes and any label will do (e.g. 208).- Positive integers should be given in the table for amino acids that you want to appear.- Negative integers can be given for amino acids you want to penalize (but not forbid).- A star (\*) can be given for amino acids that you want to require.- An explanation point (!) can be given for amino acids that you want to forbid.- Empty cells will be treated as if they contain a "0" (except for the "Res" row).- Any ill-formatted cell on this page will be highlighted in pink.- An upper bound on the library size must be given. This can be given in scientific notation (e.g. 1e7).

Multiple degenerate codon instructions

- Activate the multiple-degenerate-codon algorithm by clicking on the "Allow Mult. Deg. Codons" button below.
  This will add two rows to the table.- The user must define primer boundaries in the "Primer Boundary" row.- Indicate the first residue in a primer with the "|" (pipe) symbol.- Indicate that a residue belongs in the same primer as the previous residue with the "-" (dash) symbol.- The maximum number of degenerate codons ("Max DCs") to consider at each position should an integer;
          enumerating the combinations of degenerate codons when more than 2 are requested at any position can
          be very time consuming. 5 is the recommended maximum.- Allowing multiple degenerate codons means purchasing extra oligos (primers). Indicate the number of
            oligos you are willing to purchase total in the "Maximum Primers Total" box below the table. This number
            should be more than the number of stretches you have, and will be increased to the number of stretches
            you have if a smaller value is given. If you have 5 stretches and say you are willing to purchase 6 oligos,
            then the algorithm will only be able to use multiple degenerate codons at a single position.

Saving Data

To save the data you have entered in the table below for future sessions, switch to the "CSV Input" tab.
This will display your data in a "comma separated value" (CSV) format. Copy your data
out of the text box that appears and put it in a document for later use. You may load data into the table
by pasting the data from a previously-saved table and clicking on the "Update Table" button (which is
only visible in the "CSV Input" tab). If you get an error message saying that it found 46 columns instead of
23, make sure you clear out the text window before you paste your data in it.

Generate Report!

Update Table

CSV input instructions

To use the "comma separated value" (CSV) format simply pasted your comma-delimited data in the textbox.
The input text **must** take the same form as the manual input table (22 rows, the first
being the residue positions, the next 20 being the amino acids in alphabetical order by one-letter
code, the last being the stop penalty for that position). Once completed, click the 'Update Table'
button to populate the table with your inputs.

Once populated, one must still set the diversity bounds and the universal stop codon penalty (if desired)

Update Table

FASTA input instructions

To use the FASTA input, paste FASTA formatted sequences in the textbox. All sequences
must be the same length. Once completed, clicking the 'Update Table' button with populate
the input table with the frequency of each amino acid at each position in the sequence.
Positions which never vary are excluded from the table.

Once populated, one must still set the diversity bounds and the universal stop codon penalty (if desired)

Update Table

MSF input instructions

To use the MSF input, paste a MSF formatted sequence alignment in the textbox.

Once populated, one must still set the diversity bounds and the universal stop codon penalty (if desired)
