# Supplementary material for: Design of protein-binding proteins from the target structure alone
Source: Nature. 2022 Mar 24;605(7910):551–60. doi: 10.1038/s41586-022-04654-9 (PMC9117152; doi:10.1038/s41586-022-04654-9)
Supplement: Supplementary file 3 — Supplementary Code Design scripts and main pdb files. This compressed file is the main supplement and contains the following files: => cao_2021_protocol/ => design_models_final_combo_optimized/ => design_models_sequence/ => design_models_ssm_natives/ => ngs_analysis_scripts/ [file 41586_2022_4654_MOESM3_ESM.gz › supplemental_files/dna_production_scripts/combo_creation/swiftlib_javascript.tar.gz!/swiftlib_javascript/test.html]

Testing

This is a paragraph

 aa/pos ,268,269,270,271,272,276,330,331,332
primer,|,-,-,-,-,-,|,-,-
max dcs,2,2,2,2,2,2,2,2,2
A,3,8,1,81,4,105,10,2,1
C,0,0,0,0,0,0,0,0,0
D,8,5,0,0,0,0,29,7,9
E,23,7,0,0,0,0,21,23,17
F,0,0,0,0,0,0,0,0,0
G,0,0,0,1,0,59,4,1,5
H,1,0,0,0,0,0,0,1,9
I,10,2,98,0,0,0,0,0,1
K,22,0,0,0,0,0,5,4,9
L,17,8,5,0,0,0,0,14,0
M,13,9,5,0,2,0,3,7,4
N,6,4,0,0,0,0,8,19,15
P,0,0,0,0,0,0,0,0,0
Q,42,2,0,0,1,0,6,29,18
R,35,4,0,0,0,0,27,12,21
S,7,44,8,113,43,36,45,34,29
T,6,58,23,4,30,0,42,46,57
V,7,49,60,1,120,0,0,0,5
W,0,0,0,0,0,0,0,1,0
Y,0,0,0,0,0,0,0,0,0
 go!
