# Supplementary material for: Design of protein-binding proteins from the target structure alone
Source: Nature. 2022 Mar 24;605(7910):551–60. doi: 10.1038/s41586-022-04654-9 (PMC9117152; doi:10.1038/s41586-022-04654-9)
Supplement: Supplementary file 3 — Supplementary Code Design scripts and main pdb files. This compressed file is the main supplement and contains the following files: => cao_2021_protocol/ => design_models_final_combo_optimized/ => design_models_sequence/ => design_models_ssm_natives/ => ngs_analysis_scripts/ [file 41586_2022_4654_MOESM3_ESM.gz › supplemental_files/dna_production_scripts/combo_creation/swiftlib_javascript.tar.gz!/swiftlib_javascript/deg_codon_converter.html]

Degenerate codon  GO!
